# Prevalence of Lyme Disease and Relapsing Fever *Borrelia* spp. in Vectors, Animals, and Humans within a One Health Approach in Mediterranean Countries

**DOI:** 10.3390/pathogens13060512

**Published:** 2024-06-17

**Authors:** Myrto Koutantou, Michel Drancourt, Emmanouil Angelakis

**Affiliations:** 1 Diagnostic Department and Public Health Laboratories, Hellenic Pasteur Institute, 11521 Athens, Greece; 2 MEPHI, Aix-Marseille University, IRD, 13005 Marseille, France

**Keywords:** One Health, Lyme Disease, relapsing fever, *Borreliella*, *Borrelia*, bacteria, tick-borne, louse-borne, human-animal-environment interaction, zoonoses

## Abstract

The genus *Borrelia* has been divided into *Borreliella* spp., which can cause Lyme Disease (LD), and *Borrelia* spp., which can cause Relapsing Fever (RF). The distribution of genus *Borrelia* has broadened due to factors such as climate change, alterations in land use, and enhanced human and animal mobility. Consequently, there is an increasing necessity for a One Health strategy to identify the key components in the *Borrelia* transmission cycle by monitoring the human-animal-environment interactions. The aim of this study is to summarize all accessible data to increase our understanding and provide a comprehensive overview of *Borrelia* distribution in the Mediterranean region. Databases including PubMed, Google Scholar, and Google were searched to determine the presence of *Borreliella* and *Borrelia* spp. in vectors, animals, and humans in countries around the Mediterranean Sea. A total of 3026 were identified and screened and after exclusion of papers that did not fulfill the including criteria, 429 were used. After examination of the available literature, it was revealed that various species associated with LD and RF are prevalent in vectors, animals, and humans in Mediterranean countries and should be monitored in order to effectively manage and prevent potential infections.

## 1. Introduction

One Health approach is a holistic perspective that deals with complicated issues involving human, animal, and environmental health [1]. Such approaches have become increasingly important in recent years due to critical changes that have altered the interactions among humans, animals, and their environment [2]. Human population growth as well as their expansion into new geographical areas results in closer proximity between people and animals, facilitating the transmission of diseases among them [3,4]. Additionally, climate change and alterations in land use, along with increased movement of people, animals, and animal products through international travel and trade, further exacerbates the spread of endemic diseases [5,6].

*Borrelia* spp. are microaerophilic, slow-growing spirochetal bacteria that have been associated with human diseases [7]. Depending on the infecting species, it can cause Lyme Disease (LD) [8], also known as Lyme borreliosis, or Relapsing Fever (RF) [9].

LD is the most prevalent vector-borne disease in moderate climates of the northern hemisphere with an increasing incidence [10]. It is estimated that approximately 476,000 cases are diagnosed and treated annually in the United States [11], and over 200,000 cases per year in Europe [12].

RF can be either louse-borne (LBRF) or tick-borne (TBRF) [13] and is predominantly found in Africa, Spain, Saudi Arabia, Asia, and western United States, with epidemics still occurring in developing countries due to poor living conditions, famine, and war [14]. Currently, LBRF is mostly observed in the Horn of Africa and among immigrants originating from endemic areas [15]. TBRF has recently been associated with travel-related disease after travelling in endemic areas [16].

*Borrelia* spp. can be transmitted by hard and soft ticks, as well as human body lice. The distribution of these arthropod vectors is closely linked to the presence of the bacteria in both animals and humans [17]. Animals serve as hosts for both ticks and bacteria, playing a crucial role in the circulation of *Borrelia* spp. [18]. Therefore, studying the distribution of ticks in relation to the surrounding environment and the prevalence of suitable hosts for ticks and *Borrelia* spp. is essential for better understanding the factors contributing to human diseases [19].

The aim of this article is to review the current data on the presence of *Borrelia* spp. in vectors, animals, and humans around the Mediterranean Sea in order to monitor their human-animal-environment interactions within a One Health approach. Such information provides insight for the better surveillance and management of *Borrelia* infections.

## 2. Methods

**Figure 1:**
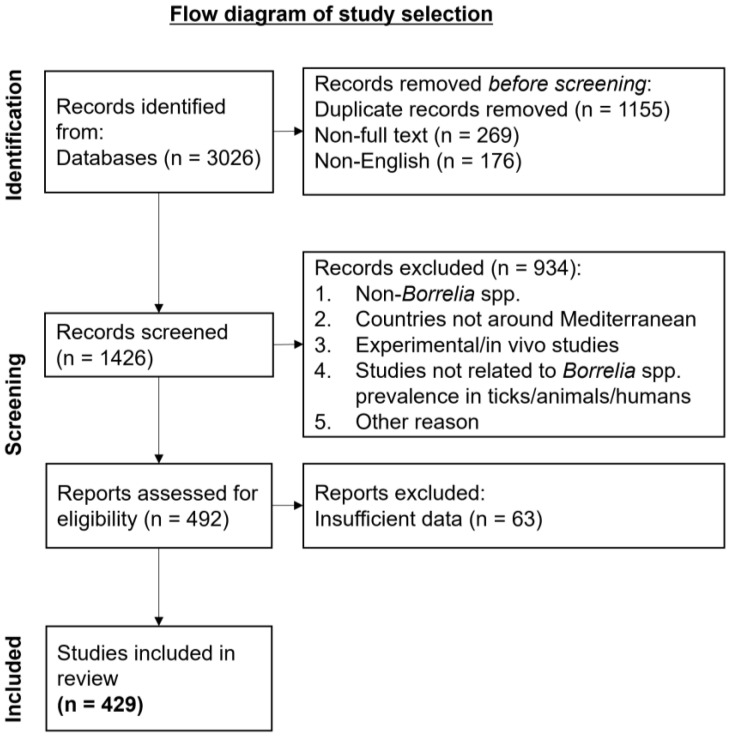
Flow diagram of study selection.

A review of the literature was conducted on the available publications from 1936 to February of 2024 by searching PubMed, Google Scholar, and Google. We included studies related to the presence of LD *Borreliella* spp. and RF *Borrelia* spp. in countries around the Mediterranean Sea in the vectors of transmission, the animal hosts, as well as the humans that can become infected. Search terms included “Lyme Disease”, “Lyme borreliosis”, “Relapsing Fever”, “*Borreliella*”, “*Borrelia*”, “bacteria”, “human-animal-environment interaction”, “One Health”, “humans”, “animals”, “ticks”, “lice”. Articles that were not written in English were excluded from this study (Figure 1).

## 3. Bacteriology

**Figure 2:**
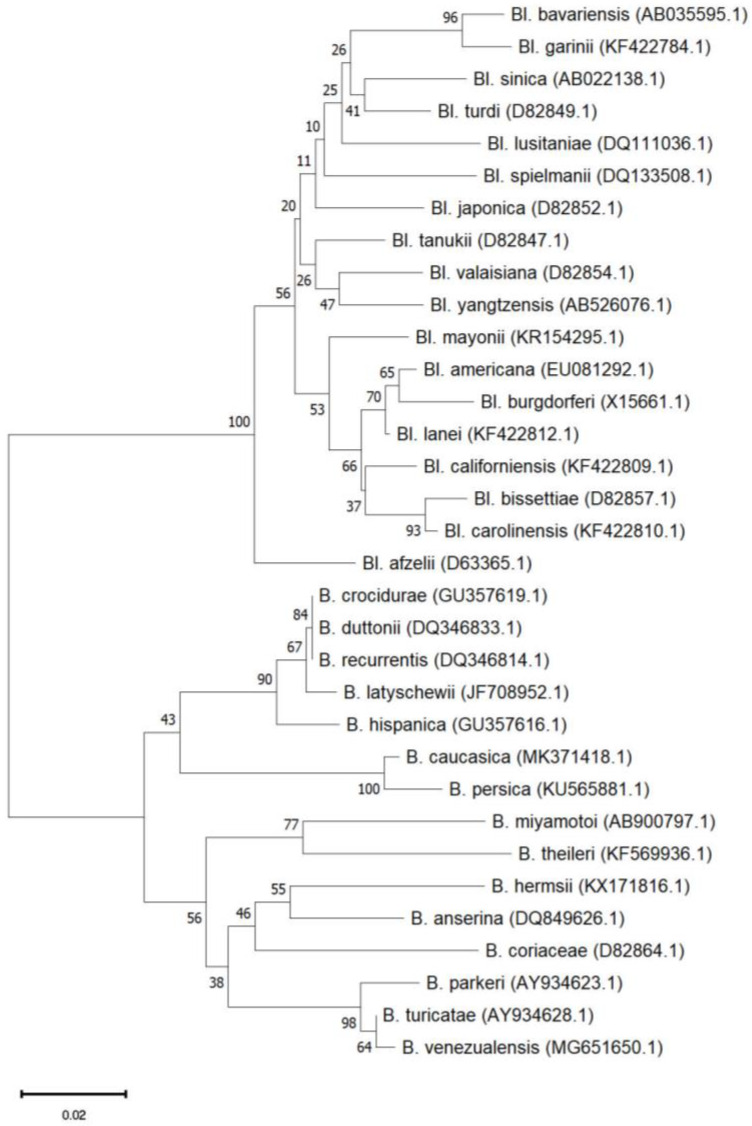
Phylogenetic tree of the genus *Borrelia* from LD and RF groups constructed with the neighbor joining method with 1000 Bootstrap replications in MEGA 11.0 software based on the flaB gene.

Species of the *Borrelia* genus are members of the Borreliaceae family of the Spirochaetales phylum and are thus characterized by the typical spirochaete (spiral) shape [20]. They are microaerophilic and host-associated since they rely on their hosts for most of their nutrients in an obligate parasitic lifestyle [21]. They possess an outer membrane around their protoplasmic cylinder analogous to that of Gram-negative bacteria, but without lipopolysaccharides [22]. They have several axial filaments attached to each pole, called endoflagella [13], between the outer membrane and the peptidoglycan layer, inside the periplasmic space, which help the bacteria move forward in a corkscrew-like motion [8]. Their genome consists of one highly conserved linear chromosome and several plasmids that can be either linear or circular and their number varies highly among strains [23]. *Borrelia* genus is divided into two genera related to human disease, *Borreliella* for the Lyme group and *Borrelia* for the Relapsing Fever group (Figure 2). Even though they belong to the same genus, they are known to have significant differences regarding their biological characteristics, their epidemiology, as well as their pathogenicity [24]. According to the phylogenetic data, although they share a genetic similarity, they are different, and they belong to independent clades under a common ancestor. For this reason, it has been proposed and recently validated [25], to include the Lyme group *Borrelia* into a new genus taxon named *Borreliella* [26].

### 3.1. LD

LD is caused by the *Borreliella* genospecies. Currently, it contains twenty-six species, twenty-three validated, and three proposed (Table 1) [27,28]. It consists of bacteria 10–20 μm long, approximately 0.3 μm in diameter and the number of their endoflagella ranges from seven to twelve [24]. The species that most commonly cause human disease are *Borreliella burgdorferi*, *Borreliella afzelii*, *Borreliella garinii*, *Borreliella mayonii*, *Borreliella spielmanii,* and *Borreliella bavariensis*. *Bl. burgdorferi* is responsible for the majority of the LD cases in North America, *Bl. garinii* in Asia, and *Bl. afzelii* along with *Bl. garinii* in Europe [29,30].

### 3.2. RF

RF is caused by the species of the relapsing fever complex, which consists of 22 validated and several proposed species (Table 2) [98,99]. Bacteria from the RF complex tend to have shorter bodies than the Lyme group and the number of their endoflagella ranges from 15 to 20 [98]. Depending on the vector of transmission, the species of the RF group are categorized in the soft-tick-borne relapsing fever (STBRF), the hard-tick-borne relapsing fever (HTBRF), and the louse-borne relapsing fever (LBRF) group [99]. Additionally, STBRF are further classified according to the geographical area into the Old World strains and the New World strains [100]. Another category is the avian RF which consists of *Borrelia anserina* and is transmitted by the soft tick *Argas* spp. [101].

## 4. One Health Approach

**Figure 3:**
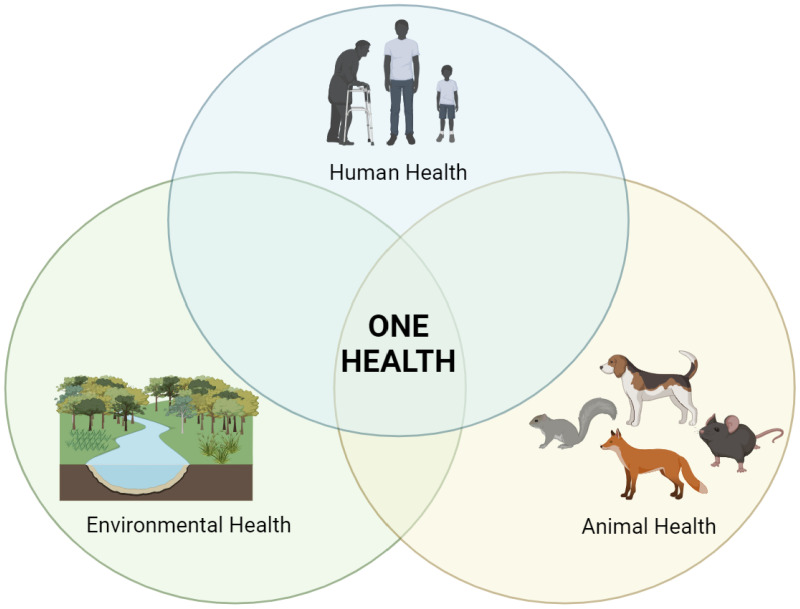
Interactions among humans, animals, and the environment within a One Health concept.

Global health challenges are escalating, and hence, One Health approaches emerge as a pivotal framework for addressing such complex health matters [153]. This holistic perspective acknowledges the interconnection of these domains and emphasizes the need for collaboration among medical, veterinary, environmental, and other relevant fields [154]. In recent years, the multifaceted nature of *Borrelia* infections has underlined the necessity for a comprehensive One Health approach. By integrating insights from diverse scientific areas, a One Health perspective offers synergistic efforts to address the complexities of *Borrelia* infections, enhancing the ability to protect both human and animal populations while promoting environmental sustainability (Figure 3) [155].

## 5. Lyme Disease

### 5.1. Clinical Manifestations

The clinical manifestations of LD can vary widely, as well as the severity and duration of the symptoms, and can affect multiple body systems [156]. The organs most frequently involved are skin, joints, nervous system, heart, and eyes [30,157]. In general, *Bl. burgdorferi* is more often associated with Lyme arthritis, *Bl. garinii* with neuroborreliosis, and *Bl. afzelii* with skin manifestations [30]. The early infection is localized, but the spirochete can spread if left untreated [158]. The most common manifestation of LD that occurs in 80–90% of the cases is a rash known as erythema migrans (EM) [159], which can have divergent characteristics [160,161]. It typically appears as an annular, erythematous rash at the site of the tick bite, which may or may not present with a central clearing [162]. It is a skin redness that appears 1–36 days (with an average of 7–14 days) after the tick bite with a tendency to expand, eventually reaching a diameter of even up to 70 cm (with a median of 10–16 cm) [163,164]. Multiple EM lesions can also occur, with a primary lesion at the site of the tick bite and secondary skin lesions caused by hematogenous dissemination [165]. Additionally, many patients also experience systemic symptoms like fatigue, fever, chills, arthralgia, myalgia, and regional lymphadenopathy [166,167]. Non-cutaneous organ involvement is also possible. Lyme neuroborreliosis occurs in approximately 12% of untreated patients who may present with cranial neuropathy (unilateral or bilateral facial nerve palsy), lymphocytic meningitis, radiculitis, or encephalitis [168,169,170]. Lyme arthritis is the most common late manifestation in the USA, presenting in approximately 60% of untreated patients, but only in a small percentage in Europe [8]. It usually appears months or even up to 2 years after initial infection and typically affects only one or a few large joints, especially the knee [171]. In rare but potentially fatal cases, Lyme carditis appears in 5% of untreated patients usually causing atrioventricular conduction block [172]. The development of chronic congestive cardiomyopathy has also been linked to LD [173,174,175].

### 5.2. Epidemiology

LD is endemic in temperate regions of the Northern Hemisphere, such as North America, Europe, and Asia [30]. The documented incidence for countries in Europe and Asia varies from negligible in the United Kingdom, Turkey, and Japan to significant in the Netherlands, Belgium, Austria, Slovenia, Lithuania, and Estonia [176,177]. Although incidents have been recorded in countries like China and Mongolia, information on the prevalence of LD in Asia is limited [178,179]. More than 90% of cases in North America are documented from only two regions of the country: the northeast and mid-Atlantic region and the north central region [180]. Both regions have grown significantly over the past 20 years and have extended up to south Canada [181,182]. LD rates are increasing worldwide [181,183] due to increased awareness [184] along with the expanding distribution of the tick vectors due to environmental factors [156,185].

#### 5.2.1. Transmission Vectors Infected with *Borreliella* spp. in Countries around the Mediterranean Sea

**Figure 4:**
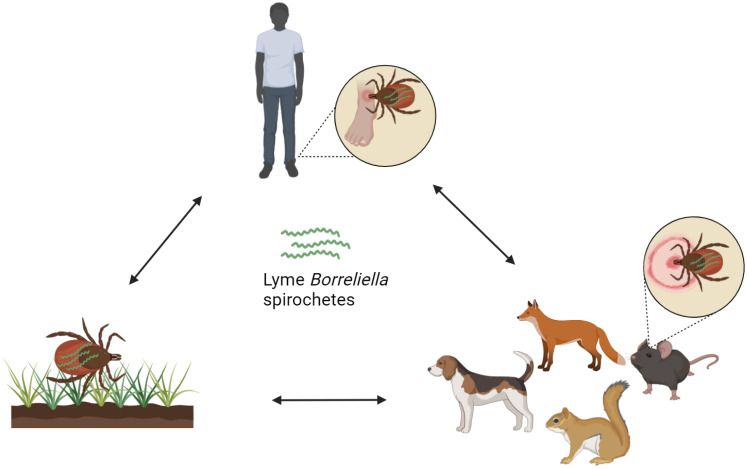
Transmission of Borreliella spirochetes.

Infectious agents causing LD are transmitted by slow-feeding hard-bodied ticks (ixodid vectors) of the *Ixodes ricinus* complex and are the only natural agents known through which humans can become infected (Figure 4) [186]. In northeastern, mid-Atlantic, and north central United States, the main vector of transmission is *Ixodes scapularis* (black-legged tick) and in the Pacific Coast states, *Ixodes pacificus* [187]. In Asia and in Eastern Europe, the main vector tick is *Ixodes persulcatus,* while in Western Europe it is *Ixodes ricinus* [8,188]. Nymphal ticks, rather than the adult ticks, are primarily responsible for transmission of the disease [189]. Spirochaetes are located mainly in the midgut of the tick before attachment and only in small numbers in the salivary glands of the ticks, however, the number in the salivary glands increases rapidly post attachment [190]. Thus, the risk of transmission increases with increased duration of tick attachment and the nymphal ticks are more likely to remain undetected for longer due to their smaller size [191]. The ticks feed on a single vertebrate host when in their larval, nymphal, and adult female phases. Adult male ticks may take sporadic small meals. When a tick, usually a larva or a nymph, feeds on an infected host, the bacteria are acquired by the tick and are subsequently transferred to its midgut [192]. The spirochetes migrate to new hosts, including humans, when the next developing stage of the tick feeds on them [193]. Nevertheless, transovarial transmission which occurs from female to offspring, is rare, resulting in a dead end for the bacteria [194]. A study concerning the human transplacental transmission has been performed, and although *Bl. burgdorferi* was not directly linked to any of the unfavorable outcomes, the frequency of adverse outcomes emphasizes the need for further studies [195].

Prevalence of the *Borreliella* complex bacteria have been identified in questing as well as feeding ticks across the Mediterranean. Nonetheless, there are no data on the following Mediterranean countries: Gibraltar, Monaco, Malta, Bosnia and Herzegovina, Montenegro, Albania, Cyprus, Syria, Lebanon, Palestine, Israel, and Libya.

##### Western Mediterranean (Spain, France)

In Spain, questing *I. ricinus* ticks were tested for the presence of *Borreliella* and *Bl. garinii* was found to be the most prevalent species followed by *Bl. valaisiana*, *Bl. lusitaniae*, *Bl. afzelii,* and *Bl. burgdorferi* [196]. Additionally, *Bl. turdi* was found in *I. frontalis* ticks collected from vegetation of urban and sub-urban areas [197] as well as in *I. frontalis*, *I. ricinus,* and *Haemaphysalis punctata* ticks collected from passerine birds [87]. Moreover, *Hyalomma lusitanicum* ticks from wild ungulates revealed that they were positive for *Bl. afzelii* [198]. *Borreliella* species have also been found in non-Ixodes ticks, as reported in studies with *Haemaphysalis* spp. ticks, *Dermacentor* spp., and *Rhipicephalus bursa* from the vegetation [199,200]. Finally, *Borreliella* DNA was reported in a male *Dermacentor reticulatus* removed from a patient [201]. In France, questing *I. ricinus* ticks from peri-urban forests demonstrated the presence of *Bl. afzelii*, *Bl. burgdorferi*, and *Bl. garinii* followed by *Bl. valaisiana*, *Bl. lusitaniae,* and *Bl. spielmanii* [202]. Moreover, *Borreliella carolinensis* was reported in one questing *I. ricinus* tick from a French forest [203]. Furthermore, ticks feeding on humans revealed the presence of *Bl. burgdorferi* in *I. ricinus*, and *Bl. garinii* in *I. ricinus* and in unidentified *Ixodes* spp. [204]. Larva ticks engorged on birds were also tested and it was found that *Borreliella* DNA was present in *I. ricinus*, *I. frontalis*, *Ixodes* spp., and *Haemaphysalis* spp. [205]. Additionally, *I. ricinus* ticks from rats and *Hyalomma marginatum* from cattle from the island of Corsica [206], as well as a questing *Dermacentor reticulatus* tick from central France were also positive for *Borreliella* DNA [207].

##### North Central Mediterranean (Italy, Slovenia)

In Italy, questing *I. ricinus* ticks, as well as *I. ricinus* ticks feeding on wild ungulates and domestic animals, were found to be infected with *Bl. afzelii*, *Bl. burgdorferi*, *Bl. garinii*, *Bl. valaisiana,* and *Bl. lusitaniae* [208,209]. Moreover, *I. ricinus* ticks feeding on humans revealed the presence of *Borreliella* DNA with *Bl. afzelii* being the most prevalent, followed by *Bl. lusitaniae*, *Bl. garinii*, *Bl. valaisiana,* and *Bl. burgdorferi* [210]. In addition, *Bl. spielmanii* and *Bl. valaisiana* were identified in *Ixodes ventalloi* and *Ixodes acuminatus* ticks feeding on feral cats and birds from Pianosa island in the Tuscany archipelago [211], and in *Hyalomma rufipes* ticks feeding on birds from Ponza island [212]. Likewise, in Slovenia, *Bl. garinii* was the most predominant species in questing *I. ricinus* ticks, followed by *Bl. afzelii*, *Bl. valaisiana*, *Bl. burgdorferi,* and *Bl. lusitaniae* [213]. Finally, *Bl. garinii* and *Bl. valaisiana* were found on *Ixodes* spp. ticks from passerine birds feeding in Slovenia [214].

##### Balkans (Croatia, Greece)

In Croatia, *I. ricinus* ticks tested positive for *Bl. afzelii*, *Bl. garinii*, *Bl. valaisiana*, *Bl. burgdorferi* [215], as well as *Bl. lusitaniae* [216]. In Greece, *Ixodes* spp. feeding on birds tested positive for *Bl. afzelii* and *Bl. garinii* [214].

##### Eastern Mediterranean (Turkey)

Questing *I. ricinus* ticks from Turkey were cultivated leading to the identification of *Bl. lusitaniae*, *Bl. afzelii*, *Bl. garinii*, *Bl. burgdorferi,* and *Bl. valaisiana* [217]. Additionally, *Bl. burgdorferi* was found in *Rhipicephalus turanicus* collected from wild boar [218], as well as in *Hyalomma marginatum*, *Hyalomma excavatum*, *Hyalomma* spp., and *Haemaphysalis parva* ticks collected from humans [219]. *Borreliella* were also prevalent in *Rhipicephalus annulatus*, in *Hyalomma anatolicum*, and *I. ricinus* questing or feeding on humans, pets, or other animals [220].

##### Southern Mediterranean (Egypt, Tunisia, Algeria, Morocco)

Several tick species feeding on one-humped camels from Cairo, Egypt, demonstrated the presence of *Bl. afzelii* in *Hyalomma dromedarii* and *H. marginatum*, as well as *Bl. burgdorferi* in *H. dromedarii* and *Amblyomma testudinarium* [221]. Moreover, *Borreliella* were also identified in *Rhipicephalus sanguineus* from dogs and in *Hyalomma anatolicum excavatum* from cattle [222]. In Tunisia, questing *I. ricinus* ticks were positive for *Borreliella* DNA [223] and after cultivation, *Bl. lusitaniae* and *Bl. garinii* were isolated [223,224]. In Algeria, questing *I. ricinus* ticks were infected with *Bl. garinii* [225] and *I. ricinus* ticks feeding on cattle with *Bl. lusitaniae* [216]. In Morocco, questing *I. ricinus* ticks were found to be infected with *Bl. lusitaniae*, *Bl. garinii,* and *Bl. burgdorferi* [226].

**Figure 5:**
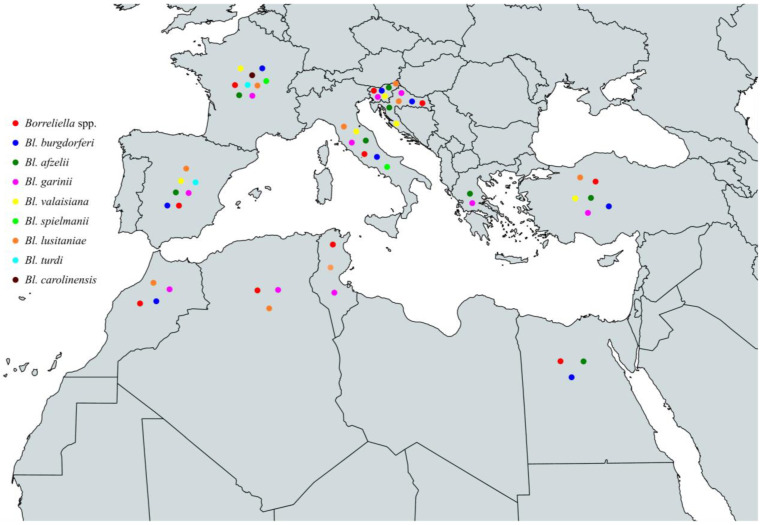
Map of *Borreliella* spp. in vectors in countries around the Mediterranean Sea.

In conclusion, the characterized species of the *Borreliella* complex that have been identified among ticks throughout the Mediterranean basin are *Bl. burgdorferi*, *Bl. afzelii*, *Bl. garinii*, *Bl. valaisiana*, *Bl. spielmanii*, *Bl. lusitaniae*, *Bl. turdi,* and *Bl. carolinensis.* Specifically, *Bl. burgdorferi* has been identified in Spain, France, Italy, Slovenia, Croatia, Turkey, Egypt, and Morocco, *Bl. afzelii* in Spain, France, Italy, Slovenia, Croatia, Greece, Turkey, and Egypt, *Bl. garinii* in Spain, France, Italy, Slovenia, Croatia, Greece, Turkey, Tunisia, Algeria, and Morocco, *Bl. valaisiana* in Spain, France, Italy, Slovenia, Croatia, and Turkey. Additionally, *Bl. spielmanii* in France and Italy, *Bl. lusitaniae* in Spain, France, Italy, Slovenia, Croatia, Turkey, Tunisia, Algeria, and Morocco, *Bl. turdi* in Spain and France and finally, *Bl. carolinensis* in France (Figure 5 and Table 3).

#### 5.2.2. Animal Hosts Infected with *Borreliella* spp. in Countries around the Mediterranean Sea

Reservoir hosts are important for the transmission of the bacterium, as they can become heavily infested with ticks and transmit the bacterium to them when they feed on their blood [227]. More than 100 animal species have been identified as *Borreliella* hosts, including small mammals, birds, and lizards [70,228,229]. The main hosts in North America are small mammals, such as mice, chipmunks, and squirrels, with the white-footed mice being the most common [230,231,232]. In Europe and Asia, the primary hosts are rodents like voles, shrews, and mice like the yellow-necked mice, especially for *Bl. afzelii* [27,192]. Birds can also become infected with *Borreliella* and serve as important hosts mainly for *Bl. garinii* as they can make long-range displacements [233]. Although deer develop antibodies when exposed to *Borreliella* via tick bites [234], they are incompetent reservoir hosts and do not transmit the infection to feeding ticks [235,236,237].

Infections of the *Borreliella* complex have been described in several animal species across the Mediterranean. However, there are no data on animal infections and the species involved in the following Mediterranean countries: Gibraltar, Monaco, Malta, Montenegro, Cyprus, Syria, Lebanon, Palestine, Israel, Libya, and Morocco.

##### Western Mediterranean (Spain, France)

In Spain, *Borreliella* have been identified in several host species. Wood mice tested positive for *Borreliella* and voles for *Bl. afzelii* [199]. Additionally, spleen samples from wild ungulates revealed the prevalence of *Bl. afzelii* [198]. Antibodies against *Borreliella* were identified in dogs [238], in red foxes [239], and in wolves [240]. Finally, chamois [241] and roe deer [198] were also presenting antibodies. In France, rodents have been identified as important host species of *Borreliella* [242,243]. Indeed, Siberian chipmunks were infected with *Bl. burgdorferi*, *Bl. afzelii*, *Bl. garinii* [244], and *Bl. spielmanii* [245], bank voles with *Bl. afzelii*, *Bl. burgdorferi,* and *Bl. garinii* [245] and wood mice with *Bl. afzelii* [245]. Additionally, red squirrels have also tested positive for *Bl. burgdorferi*, *Bl. afzelii,* and *Bl. garinii* [246]. Moreover, antibodies against *Borreliella* have also been identified in dogs [247], horses, and ponies [248]. Last but not least, skin biopsy specimens from deer and roe deer were DNA-positive for *Bl. burgdorferi*, *Bl. garinii* or *Bl. afzelii*, and one *Bl. burgdorferi* specimen was also positive in culture, indicating the presence of alive spirochetes in deer skin [249].

##### North Central Mediterranean (Italy, Slovenia)

In Italy, blood samples from rodents were positive for *Borreliella* DNA [250], and ear biopsies from bank voles and wood or yellow-necked mice revealed the presence of *Bl. afzelii*, *Bl. valaisiana,* or *Bl. lusitaniae* [251]. Foxes were also found to host several *Borreliella* genospecies, specifically *Bl. burgdorferi*, *Bl. afzelii*, *Bl. garinii*, *Bl. valaisiana*, *Bl. lusitaniae,* or *Bl. bissettiae,* and one fox was co-infected with both *Bl. afzelii* and *Bl. bissettiae* [252]. Antibodies against *Borreliella* were identified in dogs from rural as well as from urban areas [253,254], as well as in horses [255]. Furthermore, *Bl. lusitaniae* DNA has also been identified in horses [256] and in wall lizards from southern Italy [257]. Besides the animals mentioned above, wild brown hares captured in central Italy were found to carry anti-*Borreliella* antibodies [258], and one blood sample from a wild boar was positive for *Borreliella* DNA [259]. Additionally, farm-reared pheasants were seropositive [260] and feral pigeons were positive for the presence of *Borreliella* DNA [261]. Finally, it was demonstrated that *Borreliella* DNA was found in blood samples collected from the heart of hunted red deer [262], as well as antibodies against *Borreliella* in blood samples from fallow deer from farms [263]. In Slovenia, *Borreliella* DNA was found in heart and lung biopsies from rodents including yellow-necked mice, bank voles, wood mice, Mediterranean water shrews, and a white-toothed shrew [264], and after cultivation, *Bl. afzelii* was isolated [264]. In addition, antibodies against *Borreliella* were found in deer [265] and in dogs [266].

##### Balkans (Croatia, Bosnia and Herzegovina, Albania, Greece)

In Croatia, rodents from eight different collection sites were PCR-positive for *Bl. afzelii* [267] and a small seropositivity against *Borreliella* was identified in dogs [266,268,269]. Dogs were also seropositive in Bosnia and Herzegovina against *Borreliella* [266] and in Albania, for *Bl. garinii* [270]. In Greece, dogs [271,272], sheep [273], and horses [274] were also found to present antibodies against *Borreliella*.

##### Eastern Mediterranean (Turkey)

In Turkey, antibodies against *Borreliella* were identified in dogs and horses [275] and against *Bl. afzelii* in mice from North Turkey [276]. Finally, sick house cats tested positive for *Borreliella* DNA [277].

##### Southern Mediterranean (Egypt, Tunisia, Algeria)

In Egypt, blood samples from cattle were analyzed and *Borreliella* DNA was detected [222]. Additionally, dogs were found to have anti-*Borreliella* antibodies [278], as well as *Borreliella* DNA [279]. Moreover, *Borreliella* DNA was also identified in blood samples from healthy one-humped camels [221]. In Tunisia, *Borreliella* DNA was found in blood samples from goats, sheep, cattle, and camels [280]. Furthermore, lizards were also positive for *Bl. lusitaniae* as demonstrated by DNA identification from skin, liver, and/or kidney tissue samples, as well as by cultivation of a skin sample [281]. Finally, in Algeria, anti-*Borreliella* antibodies were found in dogs [282], as well as in horse blood samples [283].

**Figure 6:**
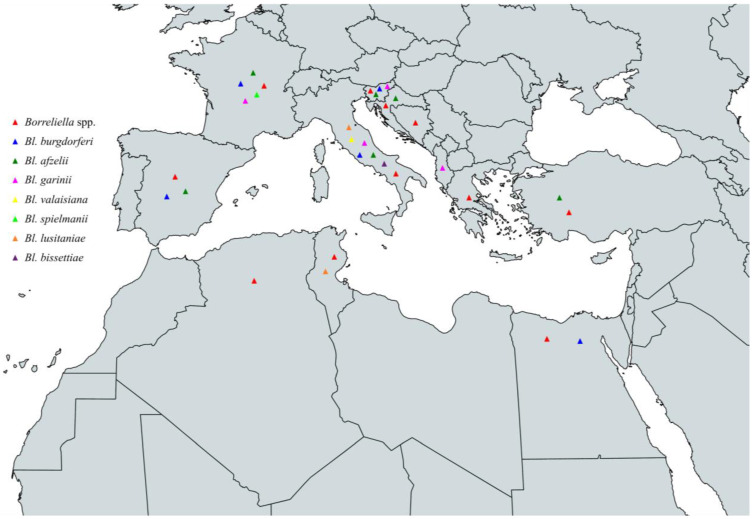
Map of *Borreliella* spp. in animals in countries around the Mediterranean Sea.

In conclusion, the circulating *Borreliella* spp. that have been characterized from animals around the Mediterranean are *Bl. burgdorferi*, *Bl. afzelii*, *Bl. garinii*, *Bl. valaisiana*, *Bl. spielmanii*, *Bl. bissettiae,* and *Bl. lusitaniae*. Specifically, *Bl. burgdorferi* has been identified in Spain, France, Italy, Slovenia, and Egypt, *Bl. afzelii* in Spain, France, Italy, Slovenia, Croatia, and Turkey, *Bl. garinii* in France, Italy, Slovenia, and Albania, *Bl. valaisiana* in Italy, *Bl. spielmanii* in France, *Bl. bissettiae* in Italy, and *Bl. lusitaniae* in Italy and Tunisia (Figure 6 and Table 4).

#### 5.2.3. *Borreliella* spp. in Humans in Countries around the Mediterranean Sea

Many *Borreliella* infections in humans are reported using molecular and/or serological assays around the Mediterranean and are associated with different genospecies of the *Borreliella* complex. However, up to date, there are no reports of human infection or very little is known on its prevalence and the species involved in the following Mediterranean countries: Gibraltar, Monaco, Malta, Montenegro, Cyprus, Syria, Lebanon, Palestine, Libya, Tunisia, Algeria, and Morocco.

##### Western Mediterranean (Spain, France)

In Spain, serum samples from the general population [284] as well as from patients bitten by ticks [201] were found to have antibodies against *Bl. burgdorferi* using the *Bl. burgdorferi* B31 strain. Additionally, *Bl. garinii* was isolated from skin biopsy specimens from patients with EM [285]. In France, synovial fluids were tested for the presence of *Borreliella* DNA, revealing that the most prevalent species was *Bl. burgdorferi*, followed by *Bl. afzelii* and *Bl. garinii* [286]. Furthermore, skin samples from patients with borrelial lymphocytoma resulted in the molecular identification of *Bl. afzelii*, *Bl. burgdorferi,* and *Bl. garinii,* which was also positive in cultivation [287]. Recently, a case of LD caused by *Bl. spielmanii* was also reported manifesting as a large EM [288].

##### North Central Mediterranean (Italy, Slovenia)

Skin biopsies from patients in Italy with clinically diagnosed LD resulted in the isolation of *Bl. afzelii*, *Bl. garinii,* and *Bl. burgdorferi* [289]. Additionally, patients with early or late LD symptoms were found to be infected with *Bl. afzelii* and *Bl. garinii*. One co-infection of *Bl. garinii* with *Bl. afzelii* and another with *Bl. valaisiana* were also reported [290]. On the other hand, serum samples from blood donors in South Tyrol revealed the presence of anti-*Bl. afzelii* and anti-*Bl. bavariensis* antibodies using Western Blot analysis [291]. Several *Borrelia* species have also been responsible for human infections in Slovenia. Culture-positive skin samples from patients with only EM as the clinical manifestation of LD indicated that the majority of the infections were due to *Bl. afzelii*, followed by *Bl. garinii* and *Bl. burgdorferi* [292]. Except from the species reported above, in Slovenia, *Bl. bavariensis* has also been isolated from humans with LD [41,293]. In addition, *Bl. spielmanii* was also isolated from skin biopsy specimens of patients with EM [294,295]. Moreover, patients with borrelial lymphocytoma were found to be infected by *Bl. afzelii*, *Bl. garinii*, or *Bl. bissettiae* [296].

##### Balkans (Croatia, Bosnia and Herzegovina, Albania, Greece)

Human infections have also been reported in the Balkan region. In Croatia, antibodies against *Bl. burgdorferi* (strain B31) were found among various population groups [297]. On the other hand, molecular identification of *Bl. afzelii* DNA was reported in serum samples from patients with EM from northwest Croatia [298]. Additionally, a woman with EM was found to be infected by *Bl. garinii* [299]. In Bosnia and Herzegovina, a case of Lyme neuroborreliosis was reported in a male patient two months after a tick bite in a forest from the southern part of the country [300] and, in Albania, the Institute of Public Health reported the diagnosis of LD cases [270] without determination of the specific *Borreliella* species responsible for the disease. Finally, in Greece, serum samples from patients with suspected zoonotic infection were tested and the presence of anti-*Borreliella* antibodies was reported [301]. Positive and equivocal samples were further analyzed by Western Blot indicating *Bl. afzelii* infection [301].

##### Eastern Mediterranean (Turkey, Israel)

In Turkey, LD cases have been documented [302], as well as seropositivity against *Bl. burgdorferi* from randomly selected subjects [303] and farmers and forestry workers [304]. A case of LD was also reported in Israel in 1991 [305].

##### Southern Mediterranean (Egypt)

Finally, in Egypt, people with close contact to feverish cattle and dogs with loss of body weight have been found positive for *Borreliella* with serological and molecular assays [222].

**Figure 7:**
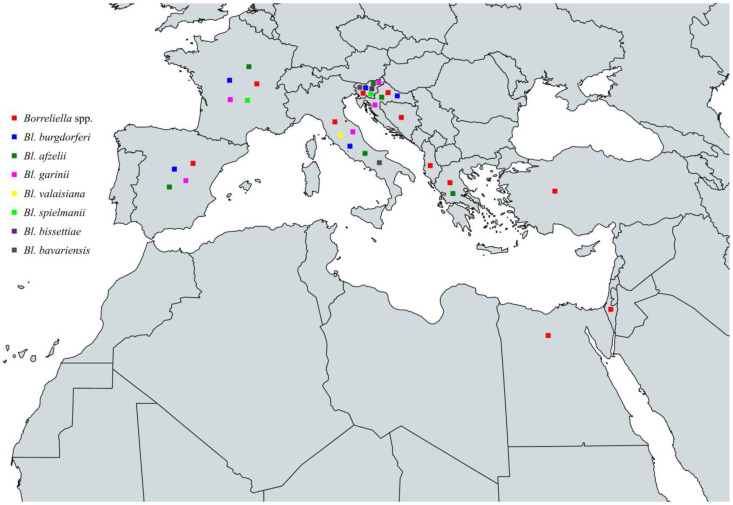
Map of *Borreliella* spp. in humans in countries around the Mediterranean Sea.

To summarize, the circulating *Borreliella* spp. from the *Borreliella* complex that have been characterized from human infections around the Mediterranean Sea are *Bl. burgdorferi*, *Bl. afzelii*, *Bl. garinii*, *Bl. valaisiana*, *Bl. bavariensis*, *Bl. spielmanii,* and *Bl. bissettiae*. Specifically, *Bl. burgdorferi* has been reported in Spain, France, Italy, Slovenia, and Croatia, *Bl. afzelii* in Spain, France, Italy, Slovenia, Croatia, and Greece, *Bl. garinii* in Spain, France, Italy, Slovenia, and Croatia, *Bl. valaisiana* in Italy, *Bl. bavariensis* in Italy and Slovenia, *Bl. spielmanii* in France and Slovenia, and *Bl. bissettiae* in Slovenia (Figure 7 and Table 5, Box 1).

Box 1 **Prevention of LD.**

Spending time in tick infested areas always carries the risk of contracting LD. However, several measures can be implemented to minimize this risk. First, tick repellent should be applied to both clothing and skin before venturing outdoors. It is also important to consistently wear long-sleeved shirts and long trousers, ensuring that they remain tucked into socks throughout the entire duration spent outside. In the case of accompanying pets, acaricides should also be used. During outdoor activi-ties, it is recommended to avoid areas with dense grass and leaf litter. After outdoor activities, a thor-ough examination should be conducted on clothing, gear, and pets for the presence of ticks. Addition-ally, inspection of the human body, including the scalp, is of great importance. If any ticks are found, appropriate removal using tweezers is essential as soon as possible. Finally, modifying the surround-ings of residential areas by clearing leaf litter, placing wood chips in areas where lawns meet forests, applying acaricides, and constructing fences to prevent the entry of tick infested animals is advisable to reduce tick populations.

## 6. Relapsing Fever

### 6.1. Clinical Manifestations

RF can be transmitted to humans through the bites of infected soft ticks, hard ticks, or body lice causing STBRF, HTBRF, and LBRF respectively. The clinical manifestations can vary, but RF hallmark is recurring episodes of high fever which can reach as high as 41 °C, spaced by afebrile periods [13]. RF patients usually experience headache, myalgia, arthralgia, and chills. Other symptoms such as fatigue, nausea and vomiting, abdominal pain and diarrhea, skin rash, neurological symptoms such as confusion and disorientation, and cardiac symptoms such as palpitations and chest pain may also occur. The severity of symptoms can vary from almost asymptomatic to lethal, depending on the species of *Borrelia* involved, the host immune response, and the stage of the infection. Untreated RF can lead to serious complications such as meningitis, hepatitis, and multi-organ failure in all three types of RF.

#### 6.1.1. TBRF

The symptoms of STBRF usually begin approximately one week after the bite of an infected tick, however the incubation period ranges from 4 to 18 days [9]. Patients typically develop an abrupt fever onset of 38.7–40 °C but can reach even up to 41 °C [99]. The first febrile episode is usually the longest and lasts for 12 h to 17 days with a median of 3 days, is frequently accompanied by nonspecific symptoms including headache, arthralgia, myalgia, and nausea, and ends with a crisis of shivering chills or rigors [306,307]. The average time between the first episode and the first relapse is 7 days and the number of recurrences of fever is up to 13 if left untreated [308]. Due to their increased neurotropism, *B. duttonii* and *B. turicatae* infections have neurological symptoms that are more severe than those of other RF pathogens, with meningitis and facial palsy among the most prevalent symptoms [102].

Untreated *B. duttonii* infections have fatality rates that range from 4 to 10%; however, if appropriate antibiotics are given right away, the death rate is less than 2% [307]. Infection during pregnancy is associated with an increased fatality rate in pregnant women, with 10–15% cases of neonatal deaths and a 43.6% perinatal lethality worldwide [309,310].

#### 6.1.2. LBRF

The incubation period is 4–18 days with a median of 7 days [311]. The symptoms initiate suddenly with a fever of almost 40 °C accompanied by stiffness, often followed by headache, anorexia, nausea, vomiting, diarrhea, and generalized pain (muscle, joint, back pain) [311,312]. The length of LBRF episodes ranges from 4 to 10 days, with a median of 5 days, and it takes 5–9 days between the episodes [307,312]. In general, patients with LBRF may have 3 to 5 febrile episodes [308]. Neurological symptoms are less frequent than in TBRF [312]. Fatality rates for untreated disease range from 30 to 80% [313], however, quick administration of the proper antibiotics reduces the fatality rate to 2–5% [306,307]. Infection during pregnancy is associated with adverse outcomes in three out of four pregnancies [313].

### 6.2. Epidemiology

#### 6.2.1. TBRF

TBRF has a worldwide distribution, with endemic regions in all continents except Australia and Antarctica [16,99]. Over the past few decades, there have been changes in its epidemiology due to the emergence of new TBRF species, the finding of hard tick RF vectors, the extension of the vectors’ geographic range, the improvements in diagnostic techniques, and the increased awareness among healthcare professionals [143,314]. Some areas such as Europe and Japan have developed into endemic areas and in contrast, others like South America and central Africa were once thought to be more endemic than they actually are [9,307]. Additionally, with instances being recorded in people who have visited endemic regions, TBRF has also become recognized as a disease connected to travel [315].

#### 6.2.2. LBRF

The epidemiology of the once-universal LBRF has radically declined during the past few decades [316]. The disappearance of this infection has been directly linked to the decline in the *Pediculus humanus humanus* lice infestation of clothing, due to improved living standards along with the use of insecticides [317]. LBRF is still endemic almost exclusively in the Horn of Africa [14], especially in Ethiopia [318,319], Somalia [320] and Eritrea [321], and it occasionally spreads to nearby countries like Sudan [308]. Remaining endemic foci of LBRF are also reported to be small parts of Asia [322] and Latin America [323]. Also, in large Mediterranean cities, homeless populations are at risk of re-emerging LBRF following reported huge lice infestation [324,325,326].

#### 6.2.3. Transmission Vectors of *Borrelia* spp. Identified in Countries around the Mediterranean Sea

**Figure 8:**
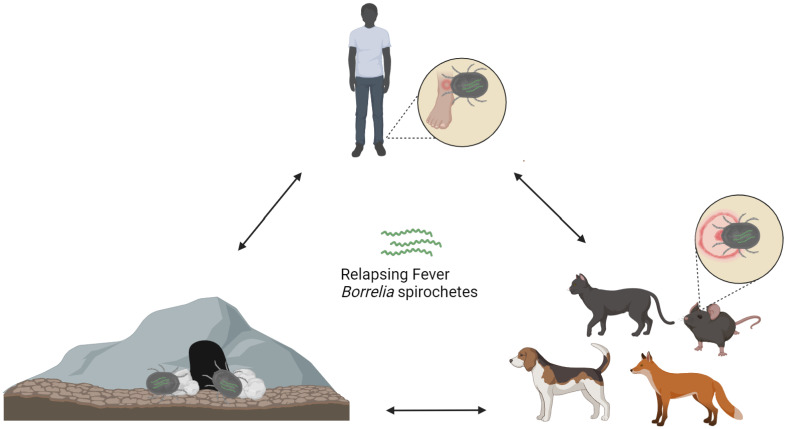
Transmission of RF Borrelia spirochetes.

Initially, it was considered that TBRF was transmitted solely by fast-feeding soft-bodied ticks (argasid vectors) of the *Ornithodoros* genus [107], however, it is now known that *Borrelia* spp. of the RF group can be transmitted from hard-ticks [143] and human body lice too [327]. In North America, *Ornithodoros hermsii* and *Ornithoodoros turicata* are the main tick species to transmit STBRF [306], along with *Ornithodoros rudis* in South America [328], *Ornithodoros erraticus* and *Ornithodoros tholozani* in Eurasia [16,329], and *Ornithodoros moubata* and *Ornithodoros sonrai* in Africa [330]. Soft tick bites are rarely noticed since they feed rapidly (15–90 min) and afterwards they return to their habitat. The STBRF-causing spirochetes enter the midgut during the blood meal of *Ornithodoros* ticks on an infected host, and in the weeks that follow, they colonize the salivary glands where they are maintained transstadially and transovarially [98]. As a result, spirochetes that colonize the salivary gland are more likely to cause an early mammalian infection, leading to rapid transmission of STBRF to the next mammalian host shortly after a bite (Figure 8) [331]. Infection can possibly occur after just 15 s of tick attachment as a tick’s salivary glands are persistently infected [332]. All tick stages are obligate blood feeders and thus potential vectors of *Borrelia* transmission [306]. Infected ticks remain infectious for their entire lifespan, and they can live up to 10 years infecting several hosts [9,333]. Hard ticks that are known to cause HTBRF are *Ixodes* spp., *Amblyomma* spp., *Dermacentor* spp., and *Rhipicephalus* spp. [138,334]. The human body louse *Pediculus humanus* is the only vector of LBRF [316]. Agents of the *Borrelia* RF group are known to infect tick species throughout the Mediterranean. However, there are insufficient data about Gibraltar, Monaco, Malta, Croatia, Bosnia and Herzegovina, Montenegro, Albania, Greece, Cyprus, Syria, Lebanon, and Libya.

##### Western Mediterranean (Spain, France)

In Spain, DNA from *B. miyamotoi* was detected in questing *I. ricinus* nymph and adult ticks [196]. This presence was confirmed across various ecological regions, including coastal, plateau, and mountain areas [335]. Nymph and male *I. ricinus* ticks feeding on roe deer were also carrying *B. miyamotoi* bacteria [336]. Additionally, on Espartar Island, a small island in the western Mediterranean Sea, *Ornithodoros maritimus* ticks from a cave occupied by small seabirds tested positive for *Borrelia* spp., showing a 99% sequence identity to *Borrelia turicatae* [337]. In France, DNA samples from free-living *I. ricinus* nymph ticks collected in the Sénart forest [338] and from the Alpes-Maritimes region [339] revealed the presence of *B. miyamotoi*. Moreover, *B. miyamotoi* was prevalent in engorged *I. ricinus* larva feeding on breeding birds [205] and in *I. ricinus* and *Haemaphysalis punctata* ticks feeding on domestic and wild animals from the island of Corsica [340]. Furthermore, *Argas vespertilionis* ticks collected from a bat-infested attic were also analyzed and a RF Group *Borrelia* sp. with a 100% sequence identity to strain CPB1 was found [341]. In addition, ticks removed from humans were also tested for the presence of RF *Borrelia* spp. and it was found that *I. ricinus* were positive [204,342].

##### North Central Mediterranean (Italy, Slovenia)

Questing *I. ricinus* ticks collected by dragging from different altitudes from the mountain areas of northwestern Italy [343], and from both the western and eastern Alps [344], tested positive for *B. miyamotoi* DNA. *B. miyamotoi* was also detected in questing *I. ricinus* ticks from two regions in Slovenia, Littoral Inner Carniola, and the Coastal-Karst area [213].

##### Eastern Mediterranean (Turkey, Palestine, Israel)

*B. miyamotoi* DNA was detected in adult *I. ricinus* ticks collected from the countryside of the European site of Turkey as well as from parks of the metropolitan area of Istanbul [345]. Moreover, *Hyalomma aegyptium* ticks feeding on *Testudo graeca* from four different collection sites of Turkey demonstrated the presence of RF *Borrelia* spp. [346]. Additionally, ticks collected from caves from several locations of the West Bank, Palestine, demonstrated the presence of *Borrelia persica* in *Ornithodoros tholozani* [347]. *B. persica* was also prevalent in nymph female and male *O. tholozani* ticks from different parts of Israel [348,349].

##### Southern Mediterranean (Egypt, Tunisia, Algeria, Morocco)

In Egypt, *B. miyamotoi* was identified in various tick species gathered from one-humped camels in Cairo and Giza. Specifically, these species include *Hyalomma dromedarii*, *Rhipicephalus annulatus*, *Amblyomma hebraeum*, *Amblyomma lepidum*, *Amblyomma cohaerens*, and *Amblyomma variegatum* [221]. Additionally, *Borrelia* spirochetes were identified in *Ornithodoros savignyi* from a village of Giza governorate [350]. Furthermore, *R. annulatus* ticks collected from donkeys tested positive for *Borrelia theileri* [351], and *Ornithodoros erraticus* ticks collected from Nile Grass Rat burrows with *Borrelia crocidurae* [352]. In Tunisia, *Rhipicephalus sanguineus* ticks feeding on hedgehogs were infected with a RF Group *Borrelia* sp. [353] and *O. erraticus* ticks collected from rodent burrows with *B. crocidurae* [354]. Additionally, *B. hispanica* was also identified in *Ornithodoros sonrai*, *O. erraticus*, and *Ornithodoros normandi* ticks from small mammal burrows collected from nine different sites [330]. In Algeria, *B. crocidurae* was also demonstrated in *O. sonrai*, *O. erraticus*, and *O. normandi* ticks from small mammal burrows collected from 18 different sites [330]. In addition, identification of *B. hispanica* DNA was found in *O. occidentalis* ticks and *B. turicatae* in *Carios capensis* ticks from seabird nests [355]. Furthermore, *Argas persicus* ticks collected from infested hen farms were also tested and it was revealed that *Borrelia anserina* was prevalent [356]. *Hyalomma aegyptium* ticks feeding on *Testudo graeca* from seven different collection sites of north Algeria as well as from 15 collection sites of central and mostly north Morocco were found to be positive for RF *Borrelia* spp. [346]. *O. sonrai* and *O. erraticus* ticks from small mammal burrows in Morocco were also tested, revealing the presence of *B. hispanica*, *B. crocidurae,* and *Borrelia merionesi* [330,357]. Finally, *Ornithodoros marocanus* complex ticks from nine sites in Morocco were found infected with *B. hispanica* and *B. crocidurae* [358].

**Figure 9:**
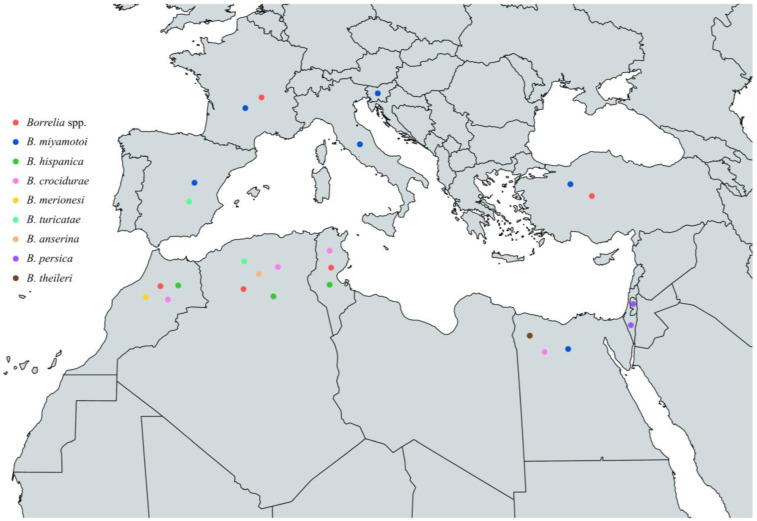
Map of *Borrelia* spp. in vectors in countries around the Mediterranean Sea.

To sum up, the circulating *Borrelia* spp. that have been identified in vectors around the Mediterranean are *B. miyamotoi*, *B. hispanica*, *B. crocidurae*, *B. merionesi*, *B. turicatae*, *B. anserina*, *B. persica,* and *B. theileri*. *B. miyamotoi* has been reported in Spain, France, Italy, Slovenia, Turkey, and Egypt, *B. hispanica* in Tunisia, Algeria, and Morocco, and *B. crocidurae* in Egypt, Tunisia, Algeria, and Morocco. In addition, *B. merionesi* has been found in ticks in Morocco, *B. turicatae* in Spain and Algeria, *B. anserina* in Algeria, *B. persica* in Palestine and Israel, and *B. theileri* in Egypt (Figure 9 and Table 6).

#### 6.2.4. *Borrelia* spp. in Animals in Countries around the Mediterranean Sea

The hosts of RF bacteria vary depending on the specific species. The majority of species can infect humans and small mammals, and a few species are also able to infect birds, as well as domestic or wild mammals [99]. The main hosts of *Borrelia hermsii* are small mammals such as chipmunks, squirrels, and mice [359] and of *Borrelia miyamotoi* are a wide range of mammals, including rodents and birds [145,360]. On the other hand, *Borrelia anserina* infects exclusively birds and is not associated with human disease [152]. Humans are the primary hosts of *Borrelia recurrentis* and *Borrelia duttonii* [102,361,362,363]. Several animal species across the Mediterranean have been found to be infected by species of the *Borrelia* RF group. Limited data are available, though, for the following Mediterranean countries: Gibraltar, Monaco, Malta, Bosnia and Herzegovina, Montenegro, Albania, Cyprus, Syria, Lebanon, Palestine, and Libya.

##### Western Mediterranean (Spain, France)

In Spain, *B. hispanica* has been identified in dogs and a street cat through positive blood smear tests and molecular analysis of 16S rRNA which revealed a 100% similarity with *B. hispanica* for all samples [364]. In France, research on the prevalence of RF spirochetes in bank voles confirmed the presence of *B. miyamotoi* [365].

##### North Central Mediterranean (Italy, Slovenia)

In Italy, pigeons were discovered to have antibodies against *B. anserina*, after analysis with surface immunofluorescence assay (SIFA) and Western blotting [151]. However, in Slovenia, molecular analysis of rodent biopsy specimens indicated the presence of *B. miyamotoi* [264].

##### Balkans (Croatia)

In Croatia, *B. miyamotoi* has been identified in various rodents, including common shrews, yellow-necked mice, and striped field mice [267].

##### Eastern Mediterranean (Turkey, Palestine, Israel)

*B. miyamotoi* has been identified in blood samples from wild rodents from Turkey, also with the highest infection rate in Ural field mice, followed by yellow-necked mice, and bank voles [366]. In addition, in West Bank, Palestine, rodents [349] and rock hyraxes [367] were found to be infected with *B. persica*. In Israel, several species have been identified as potential hosts for RF *Borrelia* spp.. Natural infection of *B. persica* in cats and dogs has been confirmed with positive blood smears and molecular assays [368], as well as with serological assays [369]. Moreover, molecular assays with blood samples from rodents and canids from all over Israel demonstrated that the species most commonly infected with *B. persica* was the social vole, followed by the red fox, the fat sand rat, the golden jackal, and the Cairo spiny mouse [349]. Additionally, besides golden jackals and red foxes, striped hyenas and European badgers also tested positive for *B. persica* DNA [370]. Finally, *B. persica* was also identified in blood and spleen samples from rock hyraxes [367].

##### Southern Mediterranean (Egypt, Tunisia, Algeria, Morocco)

In Egypt, serum samples from various animals were tested for anti-*Borrelia* spp. antibodies, with the highest infection rate found in camels, followed by sheep, goats, cows, and buffaloes [350]. Furthermore, blood samples from healthy one-humped camels from Cairo and Giza indicated infections with *B. crocidurae* and *B. miyamotoi* [221]. In addition, *B. theileri* has been identified in cattle [371,372] and in sheep blood [372,373]. In Tunisia, blood samples from fat sand rats were examined microscopically and found to be infected with a *Borrelia* spp. which could not be further characterized [374]. In Algeria, blood samples from cattle were examined, revealing an infection with RF *Borrelia* species [375]. Furthermore, *B. theileri* has also been identified in blood samples from sheep and goats by molecular assays [376]. In Morocco, rodents underwent testing for the presence of *Borrelia* spp. Thick blood film analysis was conducted in blood samples and molecular assays on brain tissue samples, revealing the presence of spirochetes in both blood and brain tissue samples [330,357]. Positive samples were not further determined to species level, except from one area, where *B. merionesi* was identified [357]. *B. crocidurae* has also been reported in rodent and insectivore samples [377].

**Figure 10:**
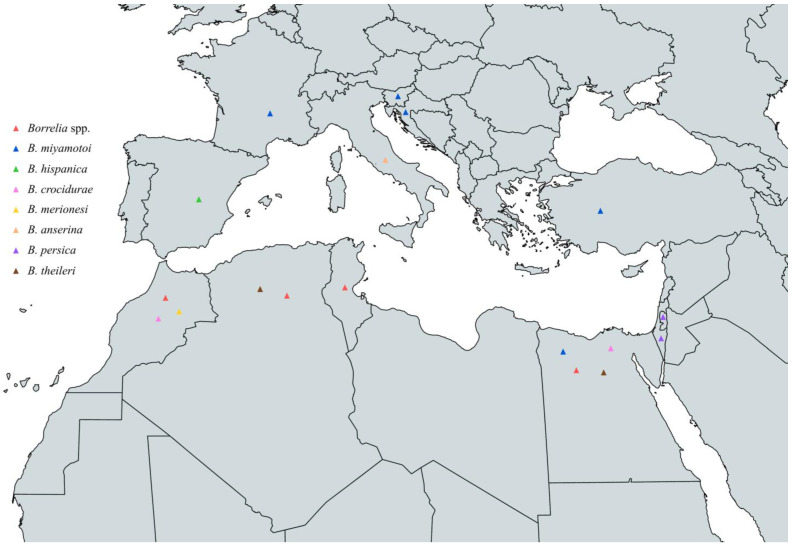
Map of *Borrelia* spp. in animals in countries around the Mediterranean Sea.

Concluding, the circulating *Borrelia* spp. that have been identified in animals across the Mediterranean are *B. miyamotoi*, *B. hispanica*, *B. persica*, *B. crocidurae*, *B. merionesi*, *B. anserina*, and *B. theileri*. *B. miyamotoi* has been reported in France, Slovenia, Croatia, Turkey, and Egypt, *B. hispanica* in Spain, *B. persica* in Palestine and Israel, *B. crocidurae* in Egypt and Morocco, *B. anserina* in Italy, *B. merionesi* in Morocco, and *B. theileri* in Egypt and Algeria (Figure 10 and Table 7).

#### 6.2.5. *Borrelia* spp. in Humans in Countries around the Mediterranean Sea

Infections due to the RF Group *Borrelia* are also present around the Mediterranean. There are no reports of human infection or very little is known on its prevalence and the species involved in the following Mediterranean countries: Gibraltar, Monaco, Malta, Slovenia, Croatia, Bosnia and Herzegovina, Montenegro, Albania, Turkey, Syria, Lebanon, Palestine, and Tunisia.

##### Western Mediterranean (Spain, France)

In Spain, RF cases have been documented in individuals within one week after exposure to ticks in mountainous regions [378] or following visits to holiday farms [314], working on a pig farm [314], or among intravenous drug users [379]. Moreover, between 1994 and 2016, numerous cases of RF were diagnosed through direct visualization of spirochetes in blood samples. Some of these samples were forwarded for identification, all of which were identified as *B. hispanica* [380]. Furthermore, from 2004 to 2015 numerous patients were diagnosed with TBRF based on microscopic analysis of blood or cerebrospinal fluid samples and had clinical data similar to those of a *B. hispanica* infection [381]. In France, *B. miyamotoi* and *B. hermsii* have been identified among patients with symptoms related to a tick bite [382]. In addition, a *B. miyamotoi* infection was confirmed by serological assays after a *B. miyamotoi*-infected tick bite [204]. Finally, *B. recurrentis* infections have been identified in homeless individuals by microimmunofluorescence [324,325] and Western Blotting [324].

##### North Central Mediterranean (Italy)

In Italy, the first autochthonous case of *B. crocidurae* infection was documented in a 51-year-old woman from Macedonia who was living in Italy from 15 years old and was diagnosed with meningitis due to *B. crocidurae* as shown by PCR from CSF specimen after a ten-day history of headache, vertical diplopia, and low-grade fever [383].

##### Balkans (Greece)

Following a two-week camping excursion on the Greek island of Tilos, during which he explored several caves, a traveler from Belgium reported experiencing recurrent febrile episodes. Upon admission to the hospital, spirochetes were detected on his blood smear, and molecular assays to identify the responsible *Borrelia* sp. confirmed a *B. persica* infection [384].

##### Eastern Mediterranean (Cyprus, Israel)

In Cyprus, a number of cases involving RF among civilians and military personnel, all of whom had somehow spent time in caves or near tick habitats, have been documented over the past century, and in some cases spirochetes were also observed in the blood smears of these individuals as well [385,386]. Also, a case of RF contracted in Cyprus and imported in the United Kingdom was published regarding a soldier who had spent three weeks on exercise in Cyprus and spent the night inside a cave where he observed ticks on his feet when he woke up [387]. In Israel, several cases of RF have been reported during the past years. Outbreaks in 2009 and 2010 have been reported among soldiers during cave entry and prolonged close contact with the ground in the context of a military exercise [388]. Additional cases have been documented, including those diagnosed by a hematological laboratory [389], neonatal cases [390] with severe Jarisch–Herxheimer reaction [391], cases who received placebos in a double-blind trial for post-exposure treatment to prevent TBRF [392], and cases confirmed by molecular assays for *B. persica* infection [348,393]. *B. persica* was also identified in spirochetemic patients from Israel as well as in patients with similar clinical manifestations, but with negative blood smears [394], and in a TBRF patient with adult respiratory distress syndrome (ARDS) [395].

##### Southern Mediterranean (Egypt, Libya, Algeria, Morocco)

A case of RF in a patient with nephritis and subarachnoid hemorrhage, confirmed by blood film, was report in Egypt after spending the night on the ground before the onset of the symptoms [396]. Additionally, humans have been found to carry antibodies against *Borrelia* spp. antigen [350]. In Libya, cases of RF with confirmed diagnosis by blood film, have been reported in one case of a young Palestinian driver who had not been anywhere else other than Benghazi for at least one month before the onset of his symptoms, an Italian P.O.W. whose specific route before the illness onset is unknown, but he came from Western Cyrenaica, an Indian pioneer who had spent his time wholly in Cyrenaica and in Benghazi, and a British lance-corporal who spent two nights in Tobruk and otherwise was totally in Benghazi [397]. In Algeria, *Borrelia* spp. DNA has been identified in feverish patients from Oran, and one sample was indicative of a new *Borrelia* spp. called *Candidatus Borrelia algerica* [103]. Finally, in Morocco, cases of RF have been confirmed in patients with unexplained fever by positive blood film [357], as well as by molecular assays demonstrating *B. hispanica* infections [398,399].

**Figure 11:**
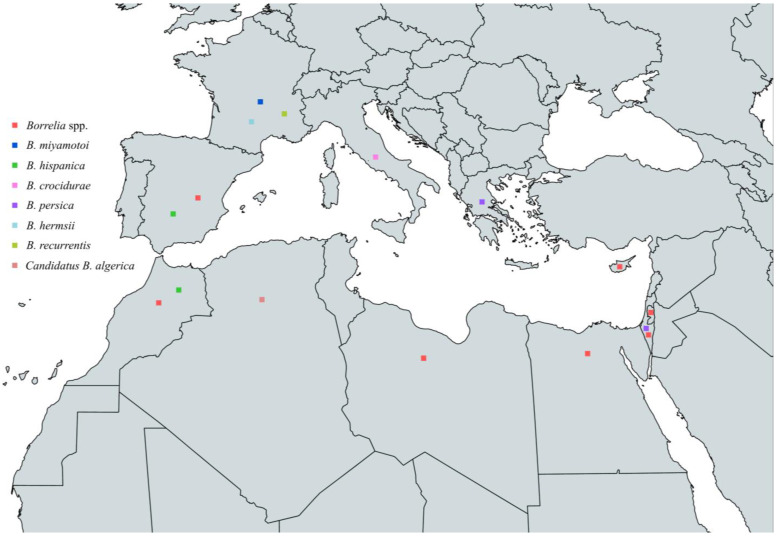
Map of *Borrelia* spp. in humans in countries around the Mediterranean Sea.

To sum up, the circulating *Borrelia* spp. that have been reported to cause human infections in countries around the Mediterranean Sea are *B. hispanica*, *B. persica*, *B. crocidurae*, *B. miyamotoi*, *B. hermsii*, *Candidatus B. algerica*, and *B. recurrentis*. *B. hispanica* has been reported in Spain and Morocco, *B. persica* in Greece and Israel, *B. crocidurae* in Italy, *B. miyamotoi* in France, *B. hermsii* in France, *Candidatus B. algerica* in Algeria, and *B. recurrentis* in France (Figure 11 and Table 8, Box 2).

Box 2 **Prevention of RF.**

Preventing TBRF relies on minimizing exposure to soft ticks. This can be achieved by avoiding habitats where soft ticks are prevalent, such as caves, animal burrows, huts, and cabins. Furthermore, it is recom-mended to use tick repellents on clothing and skin, along with wearing long-sleeved shirts and trousers tucked into socks when engaging in outdoor activities. On the other hand, preventing LBRF primarily de-pends on maintaining personal and clothing hygiene to prevent infestation by body lice.

#### 6.2.6. Imported Human Cases of RF in Mediterranean Countries

Cases of RF are not only contracted in Mediterranean countries but can also be transferred there due to travelling or immigration in other countries, especially from central Africa and Asia. In France, several cases of *B. crocidurae* infection have been identified, most of which were in immigrants from Somalia [400,401,402,403] passing through Mauritania [315] or immigrants from Mali [315]. Additionally, a case of *B. hispanica* was identified after travel in Morocco and Spain [315] and another of *B. persica* after a trip to Uzbekistan and Tajikistan [404]. In Italy, the vast majority of imported RF cases are due to *B. recurrentis* in immigrants from the Horn of Africa, e.g., Somalia, Eritrea, and Ethiopia [15,405,406]. The immigration route usually includes more or less prolonged stays in Sudan and Libya [326,407,408], and in some cases the immigration route includes Kenya and South Sudan, before crossing Sudan and Libya [409,410]. However, a case of *B. recurrentis* has also been reported in an immigrant from Mali, who passed by Algeria and Libya before arriving to Italy [411]. Also, a *B. crocidurae* infection was identified in Italy after travel to Senegal [412]. Finally, *B. recurrentis* infection has been demonstrated in Israel in Ethiopian Pilgrims [413] and in Libya and Italy in immigrants from Somalia with Germany as their final destination, who were passing through Libya and Italy as intermediate stations [14] (Table 9).

## 7. Conclusions

In order to successfully monitor and limit the spread of *Borrelia* infections, it is vital that the medical, veterinary, and environmental sectors collaborate. For this reason, we provide insight for every country around the Mediterranean Sea with available information regarding the prevalence of all *Borrelia* spp. that have been implicated in tick, animal, and human infection. In summary, LD agents were mostly found in the European part of the Mediterranean. Several *Borrelia* spp. were identified in hard ticks and in numerous animals, such as rodents, wild ungulates, farm animals, and pets. RF agents were identified all around the Mediterranean in both soft ticks as well as hard ticks. Many animal species were reported to be infected with RF agents, such as rodents, pets, and farm animals. In conclusion, infections caused by *Borrelia* spp. are still a serious issue that afflict thousands of people every year and hence demand our undivided attention.

## Figures and Tables

**Table 1:** *Borreliella* spp. of the LD Group.

*Borreliella* Species	Human Infection	Geographical Distribution	Hosts	Vectors	References
*Borreliella afzelii*	Yes	Europe, Asia	Rodents	*I. ricinus*, *I. persulcatus*, *I. pavlovsky*	[31,32,33,34]
*Borreliella americana*	Unknown	North America, Poland	Rodents, Birds	*I. pacificus*, *I. minor*, *I. ricinus*	[35,36,37]
*Borreliella andersonii*	Unknown	USA	Cottontail rabbits	*I. scapularis*, *I. dentatus*	[38,39,40]
*Borreliella bavariensis*	Yes	Europe, Asia	Rodents	*I. ricinus*, *I. persulcatus*	[41,42,43]
*Borreliella bissettiae*	Yes	USA, Europe	Rodents	*I. spinipalpis*, *I. ricinus*, *I. pacificus*	[34,44,45,46,47]
*Borreliella burgdorferi*	Yes	America, Europe, Asia	Rodents, Mammals, Birds	*I. scapularis*, *I. pacificus*, *I. ricinus*, *I. kaiseri*	[34,48,49,50]
*Borreliella californiensis*	Unknown	USA, Europe	Kangaroo Rats	*I. jellisoni*, *I. spinipalipis*, *I. pacificus*, *I. ricinus*	[34,46,51,52,53]
*Borreliella carolinensis*	Unknown	USA, Europe	Rodents	*I. minor*, *I. ricinus*, *I. affinis*	[37,54,55,56,57,58]
*Borreliella chilensis*	Unknown	Chile	Rodents	*I. stilesi*	[59]
*Borreliella finlandensis*	Unknown	Finland	Unknown	*I. ricinus*	[60]
*Borreliella garinii*	Yes	Europe, Asia	Rodents, Birds	*I. ricinus*, *I. persulcatus*	[34,61]
*Candidatus* Borreliella ibitipoquensis	Unknown	Brazil	Birds	*I. paranaensis*	[62]
*Borreliella japonica*	Unknown	Japan	Rodents	*I. ovatus*	[63,64]
*Borreliella kurtenbachii*	Unknown	North America	Rodents	*I. scapularis*	[58,65,66]
*Borreliella lanei*	Unknown	USA, Europe	Lagomorph	*I. spinipalpis*, *I. pacificus*, *I. ricinus*	[34,44,52,53,67,68]
*Borreliella lusitaniae*	Yes	Europe, North Africa	Lizards, Rodents	*I. ricinus*	[69,70,71]
*Borrelia maritima*	Unknown	North America	Unknown	*Ixodes spinipalpis*	[72]
*Borreliella mayonii*	Yes	USA	Rodents	*I. scapularis*	[73,74,75]
*Candidatus* Borrelia paulista	Unknown	Brazil	Rodents	Unknown	[76]
*Candidatus* Borrelia sibirica	Unknown	Siberia	Rodents	*I. apronophorus*, *I. persulcatus*, *I. trianguliceps*	[77]
*Borreliella sinica*	Unknown	China	Rodents	*I. ovatus*	[78]
*Borreliella spielmanii*	Yes	Europe	Rodents	*I. ricinus*	[79,80,81,82]
*Borreliella tanukii*	Unknown	Japan	Unknown	*I. tanuki*	[83,84,85]
*Borreliella turdi*	Unknown	Japan, Europe	Birds	*I. turdus*, *I. frontalis*, *I. ricinus*	[84,85,86,87]
*Borreliella valaisiana*	No	Europe, Asia	Birds	*I. ricinus*	[88,89,90,91,92]
*Borreliella yangtzensis*	Unknown	Asia	Rodents	*I. granulatus*	[93,94,95,96,97]

**Table 2:** *Borrelia* spp. of the RF Group.

*Borrelia* Species	Human Infection	Geographical Distribution	Hosts	Vectors	References
Louse-Borne Relapsing Fever					
*Borrelia recurrentis*	LBRF	Virtually worldwide, currently Ethiopia, Sudan	Human	*Pediculus humanus*	[14,102]
Old World Soft-Tick Borne Relapsing Fever					
*Candidatus Borrelia algerica*	TBRF	Algeria	Human	Unknown	[103]
*Borrelia caucasica*	STBRF	Azerbaijan, Georgia, Armenia, Ukraine	Rodents, Human	*O. verrucosus*	[104,105,106,107]
*Borrelia crocidurae*	STBRF	Western and Northern Africa	Insectivores, Rodents, Camels, Human	*O. sonrai*, *O. erraticus*	[105,107,108]
*Borrelia duttonii*	STBRF neurological signs, neonatal infection	Central, Eastern, and Southern Africa	Human	*O. moubata*	[105,107,109,110]
*Borrelia graingeri*	Unknown	East Africa	Rodents	*O. graingeri*	[105,107,111,112]
*Borrelia harveyi*	Unknown	Kenya	Monkey	Unknown	[105,113,114]
*Borrelia hispanica*	STBRF Ocular and neurological symptoms (rare)	Iberian Peninsula and Northwestern Africa	Insectivores, Rodents, Foxes, Jackals, Dogs, Human	*O. erraticus*, *O. marocanus*, *O. occidentalis*	[105,107,115]
*Candidatus Borrelia kalaharica*	STBRF	Kalahari Desert (Botswana and Namibia)	Human	*O. savignyi*	[116]
*Borrelia latyschewii*	Unknown	Central Asia, Middle East	Unknown	*O. tartakovsky*	[105,114,117]
*Borrelia merionesi*	No	Morocco and Atlantic coastal areas of the Sahara Desert	Rodents	*O. merionesi*, *O. costalis*, *O. erraticus*	[118]
*Borrelia microti*	STBRF	Iran, Afghanistan	Human	*O. erraticus*	[107,119,120,121]
*Borrelia persica*	STBRF, neurological symptoms (rare), respiratory distress (rare)	Central Asia, Middle East, Egypt, Greece	Rodents, Carnivores, Dogs, Cats, Human	*O. tholozani*	[102,105,122]
*Borrelia tillae*	No	Southern Africa	Rodents	*O. zumpti*	[123]
New World Soft-Tick Borne Relapsing Fever					
*Borrelia brasiliensis*	Unknown	Brazil	Unknown	*O. brasiliensis*	[124,125]
*Borrelia coriaceae*	No	USA	Rodents, Deer	*O. coriaceus*	[125,126,127,128]
*Borrelia dugesii*	Unknown	Mexico	Unknown	*O. dugesii*	[105,129]
*Borrelia hermsii*	STBRF, neurological symptoms (rare), neonatal infections (rare)	British Columbia, Western USA, France	Rodents, Deer, Dog, Human	*O. hermsi*	[102,105,126,128,130]
*Borrelia johnsonii*	TBRF	USA	Bats	*O talaje*	[131,132,133]
*Borrelia mazzottii*	No	Mexico	Unknown	*O. talaje*	[134]
*Borrelia parkeri*	Unknown	Western USA	Horses	*O. parkeri*	[130,135]
*Borrelia turicatae*	STBRF, Ocular and neurological symptoms	British Columbia, USA, Mexico, Africa, Europe, Asia	Rodents, Dog, Human	*O. turicata*, *O. maritimus*, *C. capensis*	[102,105,136]
*Borrelia venezualensis*	STBRF	Central America and northern South America	Human	*O. rudis*	[105,137]
Hard-Tick Borne Relapsing Fever					
*Borrelia lonestari*	STARI?	USA	Rodents, Birds, Deer, Human	*Amblyomma americanum* (lone star tick)	[133,138,139,140,141,142]
*Borrelia miyamotoi*	Flu-like syndrome, HTBRF, neurological symptoms	Asia, Europe, USA	Rodents, Birds, Human	*I. ricinus*, *I. persulcatus*, *I. scapularis*	[143,144,145,146]
*Candidatus Borrelia texasensis*	Unknown	USA (Texas)	Unknown	*Dermacentor variabilis*	[147]
*Borrelia theileri*	No	Africa, Australia, North and South America	Cattle, Sheep, Goats, Camels	*Rhipicephalus* spp.	[105,110,148,149]
Avian Relapsing Fever					
*Borrelia anserina*	No	Worldwide	Birds	*Argas* spp.	[105,110,150,151,152]

**Table 3:** *Borreliella* spp. in vectors around the Mediterranean Sea.

Country	*Borreliella* Species								
	*Borreliella* spp.	*Bl. burgdorferi*	*Bl. afzelii*	*Bl. garinii*	*Bl. valaisiana*	*Bl. spielmanii*	*Bl. lusitaniae*	*Bl. turdi*	*Bl. carolinensis*
Spain	✓	✓	✓	✓	✓		✓	✓	
France	✓	✓	✓	✓	✓	✓	✓	✓	✓
Italy	✓	✓	✓	✓	✓	✓	✓		
Slovenia	✓	✓	✓	✓	✓		✓		
Croatia	✓	✓	✓	✓	✓		✓		
Greece			✓	✓					
Turkey	✓	✓	✓	✓	✓		✓		
Egypt	✓	✓	✓						
Tunisia	✓			✓			✓		
Algeria	✓			✓			✓		
Morocco	✓	✓		✓			✓		

**Table 4:** *Borreliella* spp. in animals around the Mediterranean Sea.

Country	*Borreliella* Species							
	*Borreliella* spp.	*Bl. burgdorferi*	*Bl. afzelii*	*Bl. garinii*	*Bl. valaisiana*	*Bl. spielmanii*	*Bl. bissettiae*	*Bl. lusitaniae*
Spain	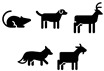	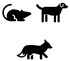	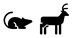					
France	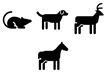	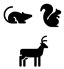	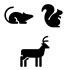	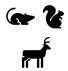		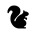		
Italy	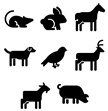	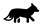	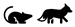	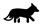	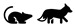		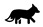	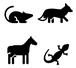
Slovenia	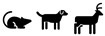	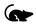	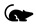	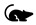				
Croatia	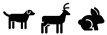		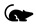					
Bosnia and Herzegovina	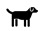							
Albania				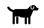				
Greece	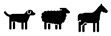							
Turkey	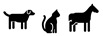		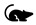					
Egypt	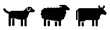	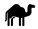						
Tunisia	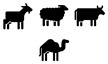							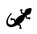
Algeria	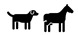							

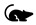
 Rodents 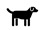 Dogs 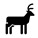 Deer 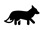 Foxes/Wolves 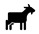 Goats/Chamois 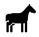 Horses 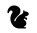 Squirrels/Chipmunks 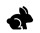 Hares 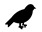 Birds 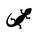 Lizards 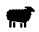 Sheep 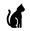 Cats 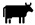 Cattle 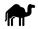 Camels 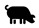 Wild boar.

**Table 5:** *Borreliella* spp. in humans around the Mediterranean Sea.

Country	*Borreliella* Species							
	*Borreliella* spp.	*Bl. burgdorferi*	*Bl. afzelii*	*Bl. garinii*	*Bl. valaisiana*	*Bl. bavariensis*	*Bl. spielmanii*	*Bl. bissettiae*
Spain	✓	✓	✓	✓				
France	✓	✓	✓	✓			✓	
Italy	✓	✓	✓	✓	✓	✓		
Slovenia	✓	✓	✓	✓		✓	✓	✓
Croatia	✓	✓	✓	✓				
Albania	✓							
Greece	✓		✓					
Turkey	✓							
Israel	✓							
Egypt	✓							

**Table 6:** *Borrelia* spp. in vectors around the Mediterranean Sea.

Country	*Borrelia* Species								
	*Borrelia* spp.	*B. miyamotoi*	*B. hispanica*	*B. crocidurae*	*B. merionesi*	*B. turicatae*	*B. anserina*	*B. persica*	*B. theileri*
Spain		✓				✓			
France	✓	✓							
Italy		✓							
Slovenia		✓							
Turkey	✓	✓							
Palestine								✓	
Israel								✓	
Egypt		✓		✓					✓
Tunisia	✓		✓	✓					
Algeria	✓		✓	✓		✓	✓		
Morocco	✓		✓	✓	✓				

**Table 7:** *Borrelia* spp. in animals around the Mediterranean Sea.

Country	*Borrelia* Species							
	*Borrelia* spp.	*B. hispanica*	*B. persica*	*B. crocidurae*	*B. miyamotoi*	*B. anserina*	*B. merionesi*	*B. theileri*
Spain		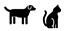						
France					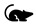			
Italy						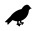		
Slovenia					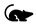			
Croatia					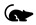			
Turkey					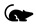			
Palestine			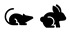					
Israel			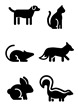					
Egypt	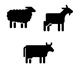			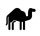	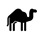			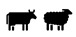
Tunisia	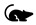							
Algeria	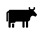							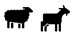
Morocco	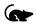			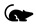			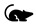	

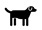
 Dogs 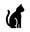 Cats 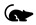 Rodents 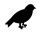 Birds 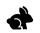 Hyraxes 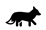 Foxes/Jackals/Hyenas 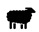 Sheep 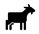 Goats 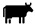 Cattle/Buffalos 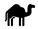 Camels 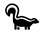 Badger.

**Table 8:** *Borrelia* spp. in humans around the Mediterranean Sea.

Country	*Borrelia* Species							
	*Borrelia* spp.	*B. hispanica*	*B. persica*	*B. crocidurae*	*B. miyamotoi*	*B. hermsii*	*Candidatus B. algerica*	*B. recurrentis*
Spain	✓	✓						
France					✓	✓		✓
Italy				✓				
Greece			✓					
Cyprus	✓							
Palestine	✓							
Israel	✓		✓					
Egypt	✓							
Libya	✓							
Algeria							✓	
Morocco	✓	✓						

**Table 9:** Imported RF cases in Mediterranean countries.

Country of Manifestation	Country of Origin/Immigration Route	*Borrelia* Species	References
France	Senegal	*B. crocidurae*	[400,401,402,403]
	Senegal and Mauritania	*B. crocidurae*	[315]
	Mali	*B. crocidurae*	[315]
	Morocco and Spain	*B. hispanica*	[315]
	Uzbekistan and Tajikistan	*B. persica*	[404]
Italy	Eritrea/Somalia/Ethiopia and Libya	*B. recurrentis*	[15]
	Somalia and Libya	*B. recurrentis*	[326,408]
	Somalia, Kenya, South Sudan, Sudan, and Libya	*B. recurrentis*	[409]
	Somalia/Sudan and Libya	*B. recurrentis*	[407]
	East Africa	*B. recurrentis*	[405]
	Mali, Algeria, and Libya	*B. recurrentis*	[411]
	Somalia, Kenya, Uganda, Sudan, and Libya	*B. recurrentis*	[410]
	Somalia	*B. recurrentis*	[406]
	Senegal	*B. crocidurae*	[412]
Israel	Ethiopia	*B. recurrentis*	[413]
Libya and Italy as intermediate destinations	Somalia, Ethiopia, Kenya, South Sudan, and Sudan	*B. recurrentis*	[14]

## Data Availability

The original contributions presented in the study are included in the article/Supplementary Material, further inquiries can be directed to the corresponding author.

## Supplementary Materials

The tables with all the references of the *Borrelia* spp. in humans, animals and vectors can be downloaded at https://www.mdpi.com/article/10.3390/pathogens13060512/s1, Table S1: Lyme Group *Borreliella* spp. in humans [41,201,222,265,270,284,285,286,287,288,289,290,291,292,293,294,295,296,297,298,299,300,301,302,303,304,305,382,414,415,416,417,418,419,420,421,422,423,424,425,426,427,428,429,430,431,432,433,434,435,436,437,438,439,440,441,442,443,444,445,446,447,448,449,450,451,452,453,454,455,456,457,458,459,460,461,462,463,464,465,466,467,468,469,470,471,472,473,474,475,476,477,478,479,480,481,482,483,484,485,486,487,488,489,490,491,492,493,494,495,496,497,498,499,500,501,502,503,504,505,506,507,508,509,510,511,512,513,514,515,516,517]; Table S2: Lyme Group *Borreliella* spp. in animals [198,199,221,222,238,239,240,241,242,243,244,245,246,247,248,249,250,251,252,253,254,255,256,257,258,259,260,261,262,263,264,265,266,267,268,269,270,271,272,273,274,275,276,277,278,279,280,281,282,283,365,373,447,496,502,518,519,520,521,522,523,524,525,526,527,528,529,530,531,532,533,534,535,536,537,538]; Table S3: Lyme Group *Borreliella* spp. in ticks [61,87,196,197,198,199,200,201,202,203,204,205,206,207,208,209,210,211,212,213,214,215,216,217,218,219,220,221,222,223,224,225,226,233,244,250,251,257,259,279,281,285,291,295,335,336,338,339,340,342,343,346,365,447,473,495,501,502,525,535,539,540,541,542,543,544,545,546,547,548,549,550,551,552,553,554,555,556,557,558,559,560,561,562,563,564,565,566,567,568,569,570,571,572,573,574,575,576,577,578,579,580,581,582,583,584,585,586,587,588,589,590,591,592,593,594,595,596,597,598,599,600,601,602,603,604,605,606,607,608,609,610,611,612,613,614,615,616,617,618,619,620,621,622,623,624,625,626,627,628,629,630,631,632,633,634,635,636,637,638,639,640,641]; Table S4: Relapsing Fever Group *Borrelia* spp. in humans [103,204,314,324,325,347,348,350,357,378,379,380,381,382,383,384,385,386,387,388,389,390,391,392,393,394,395,396,397,398,399,642,643,644,645,646,647,648,649,650,651]; Table S5: Relapsing Fever Group *Borrelia* spp. in animals [151,221,264,267,330,349,350,357,364,365,366,367,368,369,370,371,372,373,374,375,376,377,652,653]; Table S6: Relapsing Fever Group *Borrelia* spp. in ticks [196,204,205,213,221,330,335,336,337,338,339,340,341,342,343,344,345,346,347,348,349,351,352,353,354,355,356,357,358,365,447,540,551,559,562,563,566,568,570,575,576,582,643,653,654,655].
