# Supplementary material for: Prevalence of Lyme Disease and Relapsing Fever Borrelia spp. in Vectors, Animals, and Humans within a One Health Approach in Mediterranean Countries
Source: Pathogens. 2024 Jun 17;13(6):512. doi: 10.3390/pathogens13060512 (PMC11206712; doi:10.3390/pathogens13060512)
Supplement: Supplementary file 1 [file pathogens-13-00512-s001.zip › pathogens-3017594-supplementary.pdf]

Table S1 Lyme Group Borreliella spp. in humans.

#	Country	***Borreliella* species**							
		Borreliella	*Bl. burgdorferi*	*Bl. afzelii*	*Bl. garinii*	*Bl. valaisiana*	*Bl. bavariensis*	*Bl. spielmanii*	*Bl. bissetti*
1	Gibraltar								
2	Spain	✓ [1, 2]	✓ [3, 4]	✓ [5]	✓ [6]				
3	France	✓ [1, 7-26]	✓ [27-33]	✓ [27, 29, 31-40]	✓ [27, 29-33, 39-43]			✓ [44]	
4	Monaco								
5	Italy	✓ [1, 45-60]	✓ [39, 61-63]	✓ [39, 62-67]	✓ [39, 62, 63, 65, 67, 68]	✓ [67]	✓ [66]		
6	Malta								
7	Slovenia	✓ [1]	✓ [39, 69-75]	✓ [39, 69-73, 75-89]	✓ [39, 69-73, 75, 76, 78-86, 88-91]		✓ [39, 92-94]	✓ [75, 95]	✓ [96-98]
8	Croatia	✓ [99-107]	✓ [108]	✓ [99, 109-111]	✓ [110]				
9	Bosnia and Herzegovina	✓ [112]							
10	Montenegro								
11	Albania	✓ [113]							
12	Greece	✓ [114-118]		✓ [114, 119]					
13	Cyprus								
14	Turkey	✓ [1, 120-128]							
15	Syria								
16	Lebanon								
17	Palestine								
18	Israel	✓ [129, 130]							
19	Egypt	✓ [131, 132]							
20	Libya								
21	Tunisia								
22	Algeria								
23	Morocco								

Table S2 Lyme Group Borreliella spp. in animals.

#	Country	***Borreliella* species**							
		Borreliella	*Bl. burgdorferi*	*Bl. afzelii*	*Bl. garinii*	*Bl. valaisiana*	*Bl. spielmanii*	*Bl. bissettiae*	*Bl. lusitaniae*
1	Gibraltar								
2	Spain	Rodents [133, 134], Dogs [135-137], Deer [138], Foxes and wolves [139], Chamois [140]	Rodents [134], Dogs [135], Foxes [141]	Rodents [133, 142], Deer [143]					
3	France	Rodents [144], Dogs [136, 145], Deer [146, 147], Horses [148]	Rodents [149], Chipmunks [40, 149-152], Bank voles [152], Squirrels [153], Deer [154]	Small mammals [155], Rodents [149-151, 156, 157], Chipmunks [40, 149-152], Bank voles [40, 152], Wood mice [152], Squirrels [153], Deer [154]	Chipmunks [149, 150, 152], Bank voles [40, 152], Squirrels [153], Deer [154]		Chipmunks [152]		
4	Monaco								
5	Italy	Rodents [158], Hares [159], Horses [160-163], Dogs [136, 160-162, 164-170], Pheasants [171], Pigeons [172], Deer [173-175] [176], Sheep and Goats [177], Wild Boar [175]	Foxes [178]	Rodents [179], Foxes [178]	Foxes [178]	Rodents [179], Foxes [178]		Foxes [178]	Horses [180], Rodents [179], Lizards [181], Foxes [178]
6	Malta								
7	Slovenia	Rodents [96], Deer [96], Dogs [136]	Small mammals [182]	Rodents [96, 183], Small mammals [182]	Small mammals [182]				
8	Croatia	Dogs [100, 136, 184, 185], roe Deer [106], Hares [106]		Rodents [186]					
9	Bosnia and Herzegovina	Dogs [136]							

10	Montenegro								
11	Albania				Dogs [113]				
12	Greece	Dogs [187, 188], Sheep [189, 190], Horses [191]							
13	Cyprus								
14	Turkey	Dogs [192], Cats [193], Horses [192]		Rodents [194]					
15	Syria								
16	Lebanon								
17	Palestine								
18	Israel								
19	Egypt	Dogs [195, 196], Cattle [131], Sheep [197]	Camels [198]						
20	Libya								
21	Tunisia	Goats, sheep, cattle, camels [199]							Lizards [200]
22	Algeria	Dogs [201], Horses [202]							
23	Morocco								

Table S3 Lyme Group Borreliella spp. in ticks.

**Ref.**	**Vector species**	**Questing / Feeding**	**Description**	***Borreliella* species**								
				Borreliella	Bl. burgdorferi	Bl. afzelii	Bl. garinii	Bl. valaisiana	Bl. spielmanii	Bl. lusitaniae	Bl. turdi	Bl. caroline nsis
				**Gibraltar**								
				**Spain**								
[203]	7 tick species	questing	3.7% of 489 ticks from diverse geographic and ecological areas in Asturias	✓	✓	✓	✓			✓		
[204]	*Ixodes ricinus, Hyalomma lusitanicum, Rhipicephalus sanguineus*	*I. ricinus* feeding on barbary sheep, *H. lusitanicum* and *R. sanguineus* feeding on Mediterranean tortoise and *R. sanguineus* feeding on dog	*Borrelia* detected in 5.3% of tick pools (5/94): 2/5 from *I. ricinus*, 2/5 from *R. sanguineus*, and 1/5 from *H. lusitanicum*			✓	✓					
[205]	*I. ricinus*	questing	11.84%, mostly female		✓	✓	✓	✓		✓		
[206]	different species	feeding on birds	9.2% (31/336) infected, infection rates for several species available, and three co-infections of *B. turdi* and *B. valaisiana*					✓			✓	
[207]	*I. ricinus*	questing	384 ticks, positive 32.3% adult female, 18.8% nymphs, 15.6% adult male		✓	✓	✓	✓		✓		
[133]	7 tick species of the genera *Ixodes, Haemaphysalis, Dermacentor* and *Rhipicephalus*	questing	ticks 1.4% (12/845) positive nymphs and 6.1% (2/33) adults			✓	✓	✓		✓		
[208]	*I. Ricinus*	questing	nymphs		✓	✓	✓	✓		✓		
[209]	*Ixodes* spp.	feeding on cows, birds, and foxes		✓								
[210]	*I. Ricinus*	feeding on roe deer	nymphs and adult			✓	✓	✓		✓		
[143]	*Hyalomma lusitanicum* and *I. Ricinus*	feeding on wild ruminants	51.7% (46/89) total ticks positive for TBP, 50.6% *H. lusitanicum* and 75% *I. ricinus*			✓						

[211]	*I. Ricinus*	feeding on owned dogs				✓	✓	✓				
[212]	*Ixodes* spp., *I. Ricinus*	questing	2 step study, 1st step: *Ixodes* spp. 2nd step: *I. ricinus* only 9.3% adults and 1.5% nymphs		✓		✓	✓		✓		
[213]	ixodid ticks	questing		✓	✓	✓	✓					
[214]		questing and feeding	adult ticks	✓								
[215]	*Ixodes* spp.	questing	from urban and suburban area					✓			✓	
[6]	*I. ricinus*	questing	ticks		✓		✓	✓		✓		
[4]	*D. reticulatus*	from seropositive patient		✓								
[216]		questing	ticks from road edges and underpasses 5.7% (3/53) positive	✓	✓							
[217]	several tick species	feeding on passerine birds					✓	✓			✓	
[218]	Nine tick species, most frequent *Dermacentor marginatus* (55. 7%), *Ixodes ricinus* (12. 4%) and *Rhipicephalus bursa* (11. 9%)	feeding on humans		✓								
[219]	*I. ricinus*	questing and feeding on birds and rodents				✓	✓	✓				
[220]	*I. ricinus*	questing	ticks	✓								
				**France**								
[221]	Nymphal *I. ricinus*	questing	significant co-infection with Anaplasmataceae		✓	✓	✓	✓				
[222]	*I. ricinus*	questing	Nymphs, one co-infection of *B. burgdorferi s.s. and B. garinii,* study conducted with MALDI-TOF		✓	✓	✓					
[223]	Ticks (*Ixodes ricinus* for *Borrelia* spp.)	feeding on cats	Vast majority adults	✓		✓	✓	✓				

[224]	*D. marginatus* ticks	questing	18% (12/67) positive, ticks collected from an island off the west coasts of France	✓								
[225]	*Ixodes* spp.	Feeding on patients		✓		✓						
[226]	*Ixodes* spp.	Feeding on migratory birds						✓				
[227]	*I. ricinus* nymphs and adults	questing	Co-infections with *Bartonella* and *Babesia* and with both	✓								
[228]	4701 *I. ricinus* (102 female, 123 male and 4476 nymphs)	questing	Adult females significant more infected than males	✓		✓	✓					
[229]	*I. ricinus* nymphs and adults	questing	Tick density and infection rate compared for 3 years 2008, 2009, 2011		✓	✓	✓	✓	✓	✓		
[230]	*I. ricinus* nymphs and adults	questing	2-year study, nymphs, and adults from an endemic and one non-endemic region, *B. afzelii* more in nymphs, *B. garinii* and *B. valaisiana* mostly in adults and infection rates on male, female and nymphs for the 2 regions		✓	✓	✓	✓	✓			
[231]	I. ricinus adults and nymphs	questing	From 2 forests, data for infection rates between forests, between females and males and between adults and nymphs			✓	✓					✓
[232]	*I. ricinus* nymphs and adult females	questing	Infection rates		✓	✓	✓					
[156]	Female *I. ricinus* ticks	questing	3% (8/267) and one *B. miyamotoi*-infected tick positive also for *B. garinii*		✓	✓	✓	✓	✓			
[233]	Female and male adult *Dermacentor marginatus* and *Dermacentor reticulatus* ticks + *I. Ricinus*	questing	3 positives, 2 from *I. ricinus* females and 1 from *D. reticulatus* male	✓								
[234]	*I. ricinus* adults, nymphs, larvae	questing	13.2% (91/688) positive for *B. burgdorferi* s.l. and n=11 co-infections of *B. valaisiana* and *B. garinii* and other combinations		✓	✓	✓	✓				

[235]	Nymphs and adult females and males *I. ricinus*	questing	IFA on tick guts and percentages for males, females, and nymphs	✓								
[236]	Nymphs and adult females and males *I. ricinus*	questing	*B. garinii* or *B. lusitaniae*, *B. afzelii* or *B. valaisiana* but discrimination was not possible due to the given sequence	✓								
[237]	Female, male and nymphs from several species	1232 questing, 107 from patients and 56 from animals (hunted or domestic)	With details on the infection rates for every tick species	✓	✓	✓	✓	✓				
[238]	Ixodidae species	Feeding on cattle, goats, sheep and horses, domestic carnivores, wild boars, hedgehogs, mouflons, deer, birds	Pools of ticks grouped by sex, host and locality. *B. miyamotoi* in *I. ricinus* and *Ha. punctata* and *B. afzelii* in *I. ricinus*			✓						
[239]	*I. ricinus* female	questing	Percentages for every borrelia species and 5 combinations of *Borrelia* species co-infection with either 2, 3 or 4 borrelia species, statistically significant		✓	✓	✓	✓	✓			
[240]	Ticks from several species	Feeding on human		✓	✓		✓					
[241]	*I. ricinus*	questing	Nymphs, male, female with infection rates 12.4%, 2.8% and 2.9% respectively, with DFA	✓								
[242]	*I. ricinus*	questing	Nymphs, adults<br>42% *B. afzelii*, 22% *B. garinii*, 18% *B. valaisiana*, info on infection rates in 2 regions of France + coinfection data		✓	✓	✓	✓	✓	✓		

[243]	*I. ricinus*	questing	Data on infection rates for France and Germany side of Rhein Valley and %similarity of relapsing fever with B. miyamotoi, B. lonestari, B. hispanica		✓	✓	✓	✓		✓		
[244]	*I. ricinus*, I. *frontalis, Ixodes* spp., *Haemaphysalis* spp. and unidentified species	Feeding on breeding birds	Larvae<br>Microfluidic real-time PCR, info on infection rates with specific bird species and specific pathogen, co-infection data also available	✓	✓	✓	✓	✓			✓	
[245]	*Hyalomma marginatum* and *I. ricinus* respectively	Feeding on cattle and black rats	Nymphs, female and male	✓								
[246]	*I. ricinus*	questing	Nymphs, map of infection rates of the forest tested	✓								
[247]	*I. ricinus*	questing	30 females and 30 males, bacteria tested separately in salivary glands and midguts			✓	✓		✓	✓		
[248]	*I. ricinus*	questing	8.4% (59/696) 79 males, 86 females, 531 nymphs, severe co-infections, and significant tri-infections with *B. burgdorferi* s.l., *Rickettsia* spp. and Anaplasmataceae	✓	✓	✓	✓					
[155]	*I. ricinus*	questing	Comparison of two agricultural sites, one north and one south,<br>One sample positive for *B. turdi* or *B. lusitaniae*		✓	✓	✓	✓	✓	✓ or *B. turdi*	✓ or *B. lusitania e*	
[249]	*I. ricinus*	questing			✓	✓	✓	✓		✓		
[250]	*I. ricinus*	questing	78 females, 89 males, and 455 nymphs (divided in pools)	✓								
[251]	*I. ricinus*	questing	8.2% (38/461) and infection rates		✓	✓	✓					
[252]	*I. ricinus*	questing	998 nymphs, co-infections with 2 or 3 *Borrelia* species	✓	✓	✓	✓	✓	✓			
[253]	*I. ricinus*	questing	Adults and nymphs		✓	✓	✓					
[254]	*I. ricinus*	questing	Adults and nymphs, comparison between pastures and woodlands, infection rates for female, male and nymphs for each area and one co-infection	✓								
[255]	*I. ricinus*	Feeding on humans	With MALDI-TOF MS				✓					
[256]	*I. ricinus*	questing	6294 nymphs and 2300 adults (1133 Females and 1167 Males)	✓								

[257]	*I. ricinus*		Not clear if the strains were found in ticks, animals, or human samples		✓		✓					
[258]	*I. ricinus*	questing	Comparison with other places of Europe (Belgium, Germany, Estonia, Sweden)	✓								
[259]	*I. ricinus*	questing	Phylogenetic analyses and comparison with strains isolated from ticks feeding on seabirds				✓					
[260]	*I. ricinus*	questing	Nymphs, and co-infection data with *Anaplasma phagocytophilum*, SFG *Rickettsia* spp. or even all of them	✓								
[150]	*I. ricinus*	questing	10.1% (75/743) nymphs, 2 co-infections with *B. afzelii* and *B. burgdorferi* s.s.	✓	✓	✓	✓	✓	✓	✓		
[261]	*I. ricinus*	questing	6 co-infections with *B. burgdorferi* s.s., *B. garinii* and *B. afzelii* (2 species at a time)		✓	✓	✓					
[262]	*I. ricinus*	questing	Phylogenetic tree		✓	✓	✓	✓				
[263]	*I. ricinus*				✓		✓					
[40]	*I. ricinus*	questing			✓	✓	✓	✓	✓	✓		
				**Monaco**								
				**Italy**								
[264]	Several species	questing	identified with age and infection rate data available *I. ricinus* ticks 8.5% positive (from 294)		✓	✓	✓	✓		✓		
[265]	*I. ricinus*	Feeding on humans, and unknown hosts	Females and nymphs, 7.6% (13/170) feeding on humans, 8% (4/50) from unknown hosts	✓	✓	✓	✓	✓		✓		
[175]	*I. ricinus*	Feeding on deer, wild boar and chamois	8.9 (15/168) from roe deer, 7.2% (10/138) from red deer, ½ from wild boar ad 13.6% (3/22) from mouflon		✓	✓	✓	✓				
[266]	*I. ricinus*	questing	by PCR		✓	✓	✓	✓				
[181]	*I. ricinus*	questing and feeding on lizards	infection rates and details available				✓			✓		
[267]	*I. ricinus*	feeding on patients	one co-infection with *Rickettsia helvetica*	✓								
[268]	ticks of 16 species	feeding on patients	statistical data provided			✓		✓				
[269]	*I. ricinus*	feeding on domestic dogs				✓				✓		
[270]	ticks of several species	questing or engorged in wild or domestic animals	with details and statistical analysis		✓	✓	✓	✓		✓		
[179]	*I. ricinus*	feeding on rodents	21% (14/67) larvae, 0/2 nymphs		✓	✓	✓	✓		✓		

[271]	*I. ricinus*	questing	collected from same area in 2 different years with annual data provided and on tick age infection rate	✓								
[272]	*Hyalomma rufipes*	feeding on birds		✓								
[273]	*I. ricinus*	questing and engorged from hunted wild animals	but only questing ticks were positive for Borrelia spp.			✓	✓	✓		✓		
[274]	Ixodidae (data on species)	feeding on domestic dogs		✓	✓							
[275]	ticks (data on rick species provided)	feeding on patients	one female and three nymphs positive and one co-infection reported with SFG rickettsiae			✓	✓					
[276]	several tick species	feeding on patients	data on tick species age available as well as 3 co-infections		✓	✓	✓	✓		✓		
[277]	*I. ricinus*	questing				✓	✓	✓				
[278]	*I. ricinus*	questing	Nymphs, adults, by IFA and culture		✓							
[279]	*I. ricinus*	feeding on birds	with data on tick age and infection rates and 7 co-infections			✓	✓	✓		✓		
[280]	*I. ricinus*	questing	with data on tick age infection rate		✓	✓	✓	✓				
[281]	*I. ricinus*	questing	co-infected with *Ehrlicia phagocytophila* and *B. burgdorferi* sensu lato	✓								
[282]	*I. ricinus*	questing			✓	✓	✓	✓				
[283]	*I. ricinus*	questing	40% (12/30) nymphs	✓								
[284]	*I. ricinus*	questing and feeding on wild cervids and humans			✓					✓		
[285]	17 tick species	feeding on migratory birds	several co-infections	✓								
[286]	ticks of several species	questing	Different age groups and several co-infections	✓								
[287]	*I. ricinus*	questing	ticks from high altitude		✓	✓	✓	✓				
[288]	*I. ricinus*				✓		✓					
[289]	several tick species	from hunted deer and boars		✓								
[290]	*I. ricinus*	questing						✓				
[291]		questing	co-infection with *B. henselae*	✓								

[292]	*I. ricinus*	questing	6-year study of ticks, data on tick age and infection rates and annual data for all 6 years		✓	✓	✓	✓				
[293]	ticks of different species	questing	several sites including farms and a military zone inspected and co-infections with *C. burnettii, Rickettsia* spp. and *Bartonella* spp.	✓								
[294]	*I. ricinus*	questing	adults and nymphs with percentages of borrelia genospecies		✓	✓	✓			✓		
[295]	*I. ricinus*	questing	co-infection with *Borrelia burgdorferi* and *Ehrlichia phagocytophila*	✓								
[296]	several tick species	questing and feeding on feral cats and terrestrial birds	with details on infected tick origin					✓	✓			
[297]	several tick species	feeding on patients				✓	✓	✓				
[298]	*I. ricinus*	questing	7-year study		✓	✓	✓	✓				
[66]	*I. ricinus*	questing			✓	✓	✓	✓				
[299]	*I. ricinus*			✓								
[300]	*I. ricinus*	questing	from north, central, and insular areas of Italy		✓	✓	✓					
[301]	*I. ricinus*	feeding on blackbirds	larvae				✓	✓				
[302]	*I. ricinus*	questing	co-infection of 2 different *Borrelia* spp. combinations	✓	✓	✓	✓	✓				
[303]	*I. ricinus*	feeding on native small rodents and Siberian chipmunks	Co-infection with *Rickettsia monacensis*							✓		
[304]	ticks (several species)	feeding on hunters and hunting dogs								✓		
[305]		questing		✓								
[306]	*Ixodes ventalloi*	questing	*Borrelia* spp. in microbiota	✓								
[307]	*I. ricinus*	feeding on lizards, mice and passerine birds	immature ticks larval and nymphal					✓		✓		
[308]	*I. ricinus*	questing				✓				✓		
[309]	*I. ricinus*	questing	of all ages			✓				✓		
[310]	different species	questing	double or triple infections	✓								
[311]	*I. ricinus*	questing and feeding on lizards								✓		

[312]		feeding on migratory birds	co-infection with rickettsia	✓				✓				
[176]	*I. ricinus*	feeding on hunted roe deer		✓								
[158]	several tick species	feeding on rodents	different ages	✓		✓						
[313]	*I. ricinus*	questing	from 11 sampling sites and one co-infection with *A. phagocytophilum* and *B. valaisiana*			✓	✓	✓				
[314]	*I. ricinus*	feeding on lizards	3.7% of larvae and 8.0% of nymphs			✓	✓	✓		✓		
[315]	*I. ricinus*	questing	most of which nymphs	✓								
[316]	*I. ricinus*	questing	adult ticks with different sampling strategies, two of which involved only female adult ticks, double and triple co-infections		✓	✓	✓	✓				
[317]	*I. ricinus*	questing	larva, nymphs, female adult, male adult		✓	✓	✓					
[318]	*I. ricinus*	questing				✓	✓					
[319]	*I. ricinus*	questing	details on infection rates according to tick age	✓		✓	✓	✓				
[320]	*I. ricinus*	questing	nymphs from several areas	✓								
								**Malta**				
								**Slovenia**				
[321]	Several tick species	feeding on birds					✓	✓				
[322]	*I. ricinus*	questing	ticks from different areas of Slovenia 23.5% (20/85) adult and 4.4% (18/411) nymphs infected	✓								
[323]	*I. ricinus* and *Haemaphysalis punctata*	questing	from 2 areas of Slovenia by PCR and/or culture, 32.7% in one area and 33% in the other		✓	✓	✓	✓		✓		
[324]	*I. ricinus*	questing	ticks collected from six regions of Slovenia, info for infection rates and predominant species available, 35% (23/66) female, 22% (20/91) male and 13% (26/206) nymphs		✓	✓	✓					
[70]	*I. ricinus*	questing	ticks collected from six regions of Slovenia as previously described [324]		✓	✓	✓					
[75]			from Slovenia		✓	✓	✓	✓				
								**Croatia**				
[105]			from endemic region	✓								
[325]	*I. ricinus*	questing	45% (56/124) infected		✓	✓	✓	✓				
[99]	*I. ricinus*	questing	17.7% (254/1432) Croatian ticks	✓								

[106]	*I. ricinus*		from endemic area		✓	✓	✓	✓				
[326]	*I. ricinus*	questing								✓		
				**Bosnia and Herzegovina**								
				**Montenegro**								
				**Albania**								
				**Greece**								
[321]	Several tick species	feeding on birds				✓	✓					
				**Cyprus**								
				**Turkey**								
[327]	*Hyalomma aegyptium*	feeding on testudo graeca		✓								
[328]	11 tick species	feeding on patients				✓						
[329]	*I. ricinus*	questing			✓	✓	✓	✓		✓		
[330]	*I. ricinus*	questing				✓	✓	✓		✓		
[331]	*Rhipicephalus turanicus*	feeding on wild boar			✓							
[332]	*Hyalomma marginatum, Hyalomma excavatum, Hyalomma* spp. (nymph), *Haemaphysalis parva*	feeding on humans			✓							
[333]	Ticks		from meta-analysis review	✓								
[334]	*I. ricinus*	questing				✓				✓		
[335]	*R. annulatus, H. anatolicum, I. ricinus*	questing and feeding on humans, pets and other animals	1/63 (1.6%) *R. annulatus*, 1/5 (20%) *H. anatolicum* and 3/21 (14.3%) *I. ricinus*	✓								
[336]	*I. ricinus*	questing and feeding on domestic ruminants	samples from three ecologically distinct areas (wooded, transitional, and semi-arid zones)					✓				
				**Syria**								
				**Lebanon**								
				**Palestine**								

				**Israel**								
				**Egypt**								
[196]	*Rhipicephalus sanguineus*	feeding on dog		✓								
[198]		feeding on camels			✓	✓						
[131]	*Hyalomma anatolicum axcavatum* and *Rhipicephalus sanguineus*	*H. anatolicum axcavatum* from cattle and *R. sanguineus* from dogs	21.4% (3/14) *Hyalomma anatolicum axcavatum* ticks and 58.3% (7/12) *Rhipicephalus sanguineus*	✓								
				**Libya**								
				**Tunisia**								
[337]	*I. ricinus*	questing	30.5% infected, DFA assay							✓		
[200]	*I. ricinus*	questing		✓								
[338]	*I. ricinus*	questing	adult ticks	✓						✓		
[339]	*I. ricinus*	questing	with infection rates for adult, nymphs, and larvae				✓			✓		
				**Algeria**								
[326]	*I. ricinus*	feeding on cattle								✓		
[340]	different species	feeding on animals (sheep, goats, cattle, cats, dogs, chicken etc)	By MALDI-TOF but couldn't be confirmed by PCR	✓								
[341]	*I. ricinus*	questing					✓					
				**Morocco**								
[338]	*I. ricinus*	questing	adult ticks	✓						✓		
[342]	*I. ricinus*	questing as well as feeding on sheep			✓		✓			✓		

Table S4 Relapsing Fever Group Borrelia spp. in humans.

#	Country	***Borrelia* species**							
		Borrelia spp.	*B. hispanica*	*B. persica*	*B. crocidurae*	*B. miyamotoi*	*B. hermsii*	*Candidatus B. algerica*	*B. recurrentis*
1	Gibraltar								
2	Spain	✓ [343-345]	✓ [346-348]						
3	France					✓ [36, 240, 349, 350]	✓ [36]		✓ [351, 352]
4	Monaco								
5	Italy				✓ [353]				
6	Malta								
7	Slovenia								
8	Croatia								
9	Bosnia and Herzegovina								
10	Montenegro								
11	Albania								
12	Greece			✓ [354]					
13	Cyprus	✓ [355-357]							
14	Turkey								
15	Syria								
16	Lebanon								
17	Palestine	✓ [358]							
18	Israel	✓ [359-364]		✓ [365-373]					
19	Egypt	✓ [374, 375]							
20	Libya	✓ [376]							
21	Tunisia								
22	Algeria							✓ [377]	
23	Morocco	✓ [378]	✓ [379-381]						

Table S5 Relapsing Fever Group Borrelia spp. in animals.

#	Country	***Borrelia* species**							
		Borrelia spp.	*B. hispanica*	*B. persica*	*B. crocidurae*	*B. miyamotoi*	*B. anserina*	*B. merionesi*	*B. theileri*
1	Gibraltar								
2	Spain		Dogs [382], cats [382]						
3	France					Rodents [156]			
4	Monaco								
5	Italy						Birds [383]		
6	Malta								
7	Slovenia					Rodents [183]			
8	Croatia					Rodents [186]			
9	Bosnia and Herzegovina								
10	Montenegro								
11	Albania								
12	Greece								
13	Cyprus								
14	Turkey					Rodents [384]			
15	Syria								
16	Lebanon								
17	Palestine (West Bank)			Rodents [385], Rock hyraxes [386]					
18	Israel			Dogs [387], Cats [388-390], Rock hyraxes [386], Rodents, Foxes [391], Jackals					

				[385, 391], striped hyenas [391], European badger [391]					
19	Egypt	Sheep, goats, cows, buffalos [374]			Camels [198]	Camels [198]			Cattle [392, 393], Sheep [197, 393]
20	Libya								
21	Tunisia	Rodents [394]							
22	Algeria	Cattle [395]							Sheep, Goats [396]
23	Morocco	Rodents [397]			Rodents [398]			Rodents [379]	

Table S6 Relapsing Fever Group Borrelia spp. in ticks.

Ref.	Vector species	Questing / Feeding	Description	**Borrelia species**								
				Relapsing fever spp.	B. miyamotoi	B. hispanica	B. crocidurae	B. merionesi	B. turicatae	B. anserina	B. persica	B. theileri
				**Gibraltar**								
				**Spain**								
[205]	*I. ricinus*	questing	11.84%, mostly female		✓							
[207]	*I. ricinus*	questing	384 ticks, positive 32.3% adult female, 18.8% nymphs, 15.6% adult male		✓							
[208]	*I. ricinus*	questing	nymphs		✓							
[210]	*I. ricinus*	feeding on roe deer	nymphs and adult		✓							
[399]	*Ornithodoros maritimus*	questing	from cave						✓			
				**France**								
[221]	Nymphal *I. ricinus*	questing	significant co-infection with Anaplasmataceae		✓							
[349]	*I. ricinus* nymphs	questing	2.18% (94/4354) positive, ticks collected from 2013 to 2016		✓							
[400]	*Argas vespertilionis* (bat ticks)		From a bat-infested attic	✓								
[225]	*Ixodes* spp.	Feeding on patients			✓							
[232]	*I. ricinus* nymphs and adult females	questing	Infection rates		✓							
[156]	Female *I. ricinus* ticks	questing	3% (8/267) and one *B. miyamotoi*-infected tick positive also for *B. garinii*		✓							
[236]	Nymphs and adult females and males *I. ricinus*	questing	*B. garinii* or *B. lusitaniae*, *B. afzelii* or *B. valaisiana* but discrimination was not possible due to the given sequence		✓							
[237]	Female, male and nymphs from several species	1232 questing, 107 from patients and 56 from animals (hunted or domestic)	With details on the infection rates for every tick species		✓							

[238]	Ixodidae species	Feeding on cattle, goats, sheep and horses, domestic carnivores, wild boars, hedgehogs, mouflons, deer, birds	Pools of ticks grouped by sex, host and locality.<br>*B. miyamotoi* in *I. ricinus* and *Ha. punctata* and *B. afzelii* in *I. ricinus*		✓							
[239]	*I. ricinus* female	questing	Percentages for every borrelia species and 5 combinations of *Borrelia* species co-infection with either 2, 3 or 4 borrelia species, statistically significant		✓							
[240]	Ticks from several species	Feeding on human			✓							
[243]	*I. ricinus*	questing	Data on infection rates for France and Germany side of Rhein Valley and %similarity of relapsing fever with B. miyamotoi, B. lonestari, B. hispanica	✓								
[244]	*I. ricinus*, I. *frontalis, Ixodes* spp., *Haemaphysalis* spp. and unidentified species	Feeding on breeding birds	Larvae<br>Microfluidic real-time PCR, info on infection rates with specific bird species and specific pathogen, co-infection data also available		✓							
[247]	*I. ricinus*	questing	30 females and 30 males, bacteria tested separately in salivary glands and midguts		✓							
[249]	*I. ricinus*	questing			✓							
[252]	*I. ricinus*	questing	998 nymphs, co-infections with 2 or 3 *Borrelia* species		✓							
[255]	*I. ricinus*	Feeding on humans	With MALDI-TOF MS		✓							
[256]	*I. ricinus*	questing	6294 nymphs and 2300 adults (1133 Females and 1167 Males)		✓							
[40]	*I. ricinus*	questing			✓							
				**Monaco**								
				**Italy**								
[401]	*I. ricinus*	questing	ticks from 2 areas		✓							
[265]	*I. ricinus*	feeding on humans, ungulates and unknown hosts	In total, 0.6% (1/170) from humans, 1.8% (2/113) from ungulates, 2% (1/50) from unknown hosts		✓							
[287]	*I. ricinus*	questing	ticks from high altitude		✓							
				**Malta**								

				**Slovenia**								
[323]	*I. ricinus* and *Haemaphysalis punctata*	questing	from 2 areas of Slovenia by PCR and/or culture, 32.7% in one area and 33% in the other		✓							
				**Croatia**								
				**Bosnia and Herzegovina**								
				**Montenegro**								
				**Albania**								
				**Greece**								
				**Cyprus**								
				**Turkey**								
[327]	*Hyalomma aegyptium*	feeding on testudo graeca		✓								
[402]	*I. ricinus*	questing	ticks from Istanbul and countryside		✓							
				**Syria**								
				**Lebanon**								
				**Palestine**								
[366]	*O. tholozani*		from caves of the West Bank								✓	
				**Israel**								
[389]	Ticks		from cave								✓	
[365]	*Ornithodoros tholozani*		from caves								✓	
[385]	*O. tholozani*		statistical data on nymphs, larval and adult, female and male and the species of their blood meals								✓	
				**Egypt**								
[198]		feeding on camels			✓							
[403]	*Rhipicephalus annulatus*	feeding on equines										✓
[404]	*Ornithodoros erraticus*		From burrows of Nile grass rat				✓					
				**Libya**								
				**Tunisia**								
[405]	*Rhipicephalus sanguineus*	feeding on African hedgehog		✓								

[406]	*Ornithodoros erraticus*	questing	ticks from caves of 4 different climate regions with infection rates for 19.2 % (5/26) female, 27.3% (6/22) male and11.7% (13/111) nymphs				✓					
[397]	*Ornithodoros* spp.		from small mammal burrows			✓						
				**Algeria**								
[327]	*Hyalomma aegyptium*	feeding on *testudo graeca*		✓								
[397]	*Ornithodoros* spp.		from small mammal burrows				✓					
[407]	Ixodid ticks	Feeding on cattle	Data on infection rates according to each tick species	✓								
[408]	*Carios capensis* and *Ornithodoros occidentalis*		from seabirds' nest 10.4% (5/48) for *B. turicatae* and *Ornithodoros occidentalis* from rodent burrows for *B. hispanica* 8% (2/6)			✓			✓			
[409]	*Argas persicus*		collected from hen and laying hen's farm walls, 5.9% (2/34) positive							✓		
				**Morocco**								
[327]	*Hyalomma aegyptium*	feeding on testudo graeca		✓								
[397]	*Ornithodoros* spp.		from small mammal burrows			✓	✓	✓				
[410]	*Ornithodoros sonrai*		ticks from houses and private lands				✓					
[379]	*Ornithodoros erraticus* complex (*O. erraticus s.s.* and *O. sonrai*)		from rodent and insectivore burrows			✓	✓	✓				
[411]	*Ornithodoros marocanus* s.l.		species from rodent and insectivore burrows with 3% (2/65) nymphs, 5.9% (2/34) males and 15.6% (5/32) females			✓	✓					

Table 7 Imported Relapsing Fever cases in Mediterranean countries.

	**Country of manifestation**	**Country of origin / Immigration route**	***Borrelia* species**	**References**
1	France	Senegal	*B. crocidurae*	[412-415]
		Senegal and Mauritania	*B. crocidurae*	[416]
		Mali	*B. crocidurae*	[416]
		Morocco and Spain	*B. hispanica*	[416]
		Uzbekistan and Tajikistan	*B. persica*	[417]
2	Italy	Eritrea/Somalia/Ethiopia and Libya	*B. recurrentis*	[418]
		Somalia and Libya	*B. recurrentis*	[419, 420]
		Somalia, Kenya, South Sudan, Sudan, and Libya	*B. recurrentis*	[421]
		Somalia/Sudan and Libya	*B. recurrentis*	[422]
		East Africa	*B. recurrentis*	[423]
		Mali, Algeria, and Libya	*B. recurrentis*	[424]
		Somalia, Kenya, Uganda, Sudan, and Libya	*B. recurrentis*	[425]
		Somalia	*B. recurrentis*	[426]
		Senegal	*B. crocidurae*	[427]
3	Israel	Ethiopia	*B. recurrentis*	[428]
4	Libya and Italy as intermediate destinations	Somalia, Ethiopia, Kenya, South Sudan, and Sudan	*B. recurrentis*	[429]

1. Burn L, Pilz A, Vyse A, Gutierrez Raba AV, Angulo FJ, Tran TMP, Fletcher MA, Gessner BD, Moisi JC, Stark JH (2023) Seroprevalence of Lyme Borreliosis in Europe: Results from a Systematic Literature Review (2005-2020). Vector Borne Zoonotic Dis 23:195-220. doi:10.1089/vbz.2022.0069.
2. Montero E, Folgueras M, Rodriguez-Perez M, Perez-Ls L, Diaz-Arias J, Meana M, Revuelta B, Haapasalo K, Collazos J, Asensi V, Gonzalez LM (2023) Retrospective study of the epidemiological risk and serological diagnosis of human babesiosis in Asturias, Northwestern Spain. Parasit Vectors 16:195. doi:10.1186/s13071-023-05817-x.
3. Lledo L, Gegundez MI, Saz JV, Beltran M (2004) Screening of the prevalence of antibodies to Borrelia burgdorferi in Madrid province, Spain. Eur J Epidemiol 19:471-2. doi:10.1023/b:ejep.0000027349.48337.cb.
4. Lledo L, Gegundez MI, Gimenez-Pardo C, Alamo R, Fernandez-Soto P, Nuncio MS, Saz JV (2014) A seventeen-year epidemiological surveillance study of Borrelia burgdorferi infections in two provinces of northern Spain. Int J Environ Res Public Health 11:1661-72. doi:10.3390/ijerph110201661.
5. Fernandez-Jorge B, Almagro-Sanchez M, Escudero-Nieto R, Fonseca-Capdevila E (2006) [Erythema migrans due to Borrelia afzelii]. Med Clin (Barc) 126:237-8. doi:10.1157/13084875.
6. Escudero R, Barral M, Perez A, Vitutia MM, Garcia-Perez AL, Jimenez S, Sellek RE, Anda P (2000) Molecular and pathogenic characterization of Borrelia burgdorferi sensu lato isolates from Spain. J Clin Microbiol 38:4026-33. doi:10.1128/JCM.38.11.4026-4033.2000.
7. Rigaud E, Jaulhac B, Garcia-Bonnet N, Hunfeld KP, Femenia F, Huet D, Goulvestre C, Vaillant V, Deffontaines G, Abadia-Benoist G (2016) Seroprevalence of seven pathogens transmitted by the Ixodes ricinus tick in forestry workers in France. Clin Microbiol Infect 22:735 e1-9. doi:10.1016/j.cmi.2016.05.014.
8. Jaulhac B, Chary-Valckenaere I, Sibilia J, Javier RM, Piemont Y, Kuntz JL, Monteil H, Pourel J (1996) Detection of Borrelia burgdorferi by DNA amplification in synovial tissue samples from patients with Lyme arthritis. Arthritis Rheum 39:736-45. doi:10.1002/art.1780390505.
9. Riescher S, Dos Santos A, Lecomte R, Lenoble C, Guillon B (2023) A case report of unilateral cerebral vasculitis in adults: keep in mind Lyme neuroborreliosis. BMC Infect Dis 23:283. doi:10.1186/s12879-023-08259-z.
10. Bernard A, Kodjikian L, Abukhashabh A, Roure-Sobas C, Boibieux A, Denis P, Broussolle C, Seve P (2018) Diagnosis of Lyme-associated uveitis: value of serological testing in a tertiary centre. Br J Ophthalmol 102:369-372. doi:10.1136/bjophthalmol-2017-310251.
11. Jeantin L, Rodriguez-Regent C, De Martino S, Gayraud M, Cosserat J (2021) Intrathecal antibody kinetics in neuroborreliosis: A report on three cases and a literature review. Infect Dis Now 51:627-629. doi:10.1016/j.idnow.2021.02.006.
12. Christiann F, Rayet P, Patey O, Ngueodjibaye DB, Theron-le Gargasson JF, Lafaix C (1997) Lyme borreliosis in central France: a sero-epidemiologic examination involving hunters. Eur J Epidemiol 13:855. doi:10.1023/a:1007420008690.
13. Christiann F, Rayet P, Ngueodjibaye DB, Patey O, Godefroy A, Klein J, Lapegue R, Theron-Le Gargasson JF, Godfroid E, Lafaix C (1997) Endemic level of Lyme borreliosis in a region of central France: a sero-epidemiologic examination involving blood donors. Eur J Epidemiol 13:361-2. doi:10.1023/a:1007331421763.

14. Guet-Revillet H, Levy C, Vallet C, Maghraoui-Slim V, Dommergues MA, Hentgen V, Paget C, Laugel V, Cohen R, Ferroni A (2019) Lyme neuroborreliosis in children: Report of nine cases and a review of the literature. Arch Pediatr 26:133-137. doi:10.1016/j.arcped.2019.02.010.
15. Gibaud M, Pauvert O, Gueden S, Durigneux J, Van Bogaert P (2019) Opsoclonus in a child with neuroborreliosis: Case report and review of the literature. Arch Pediatr 26:118-119. doi:10.1016/j.arcped.2018.11.013.
16. Mereaux JL, Hebant B, Magne N, Quesney G, Lefaucheur R (2020) Bilateral facial palsy in an older person. Age Ageing 49:887-888. doi:10.1093/ageing/afaa082.
17. Nubling M, Rieger MA, Batsford S, Wagner M, Wertenschlag E, Hofmann F (2002) Seroprevalence of infection with Borrelia burgdorferi s. l. in two adjacent regions of eastern France and southwestern Germany. Int J Med Microbiol 291 Suppl 33:218. doi:10.1016/s1438-4221(02)80057-1.
18. Sevestre J, Benichou A, Rio V, Delaunay P, Gonfrier G, Martaresche C, Carlo V, Nakam S, Mondain V, Carles M, Jeandel PY, Durant J (2022) Emergence of Lyme Disease on the French Riviera, a Retrospective Survey. Front Med (Lausanne) 9:737854. doi:10.3389/fmed.2022.737854.
19. Letrilliart L, Ragon B, Hanslik T, Flahault A (2005) Lyme disease in France: a primary care-based prospective study. Epidemiol Infect 133:935-42. doi:10.1017/S0950268805004413.
20. Frey M, Jaulhac B, Piemont Y, Marcellin L, Boohs PM, Vautravers P, Jesel M, Kuntz JL, Monteil H, Sibilia J (1998) Detection of Borrelia burgdorferi DNA in muscle of patients with chronic myalgia related to Lyme disease. Am J Med 104:591-4. doi:10.1016/s0002-9343(98)00112-0.
21. Mercier G, Burckel A, Lucotte G (1997) Detection of Borrelia burgdorferi DNA by polymerase chain reaction in urine specimens of patients with erythema migrans lesions. Mol Cell Probes 11:89-94. doi:10.1006/mcpr.1996.0090.
22. Christiann F, Rayet P, Patey O, Lafaix C (1996) Epidemiology of Lyme disease in France: Lyme borreliosis in the region of Berry sud: a six year retrospective. Eur J Epidemiol 12:479-83. doi:10.1007/BF00144000.
23. Dh te R, Basse-Guerineau AL, Beaumesnil V, Christoforov B, Assous MV (2000) Full spectrum of clinical, serological, and epidemiological features of complicated forms of Lyme borreliosis in the Paris, France, area. Eur J Clin Microbiol Infect Dis 19:809-15. doi:10.1007/s100960000391.
24. Arzouni JP, Laveran M, Beytout J, Ramousse O, Raoult D (1993) Comparison of western blot and microimmunofluorescence as tools for Lyme disease seroepidemiology. Eur J Epidemiol 9:269-73. doi:10.1007/BF00146262.
25. Septfons A, Rigaud E, Benezet L, Velay A, Zilliox L, Baldinger L, Gonzalez G, Figoni J, de Valk H, Deffontaines G, Desenclos JC, Jaulhac B (2023) Seroprevalence for Borrelia burgdorferi sensu lato and tick-borne encephalitis virus antibodies and associated risk factors among forestry workers in northern France, 2019 to 2020. Euro Surveill 28. doi:10.2807/1560-7917.ES.2023.28.32.2200961.
26. Bonnet C, Figoni J, Souty C, Septfons A, de Martino S, de Valk H, Fournier L, Hanslik T, Jaulhac B, Blanchon T (2023) Prevalence and factors associated with a prescription of a Lyme borreliosis serology for erythema migrans diagnosis in general practice: a study from the French sentinel network, 2009-2020. BMC Prim Care 24:163. doi:10.1186/s12875-023-02108-3.
27. Lenormand C, Jaulhac B, De Martino S, Barthel C, Lipsker D (2009) Species of Borrelia burgdorferi complex that cause borrelial lymphocytoma in France. Br J Dermatol 161:174-6. doi:10.1111/j.1365-2133.2009.09100.x.

28. Zhioua E, Rodhain F, Binet P, Perez-Eid C (1997) Prevalence of antibodies to Borrelia burgdorferi in forestry workers of Ile de France, France. Eur J Epidemiol 13:959-62. doi:10.1023/a:1007465305193.
29. Corre C, Coiffier G, Le Goff B, Ferreyra M, Guennic X, Patrat-Delon S, Degeilh B, Albert JD, Tattevin P (2022) Lyme arthritis in Western Europe: a multicentre retrospective study. Eur J Clin Microbiol Infect Dis 41:21-27. doi:10.1007/s10096-021-04334-y.
30. Jaulhac B, Heller R, Limbach FX, Hansmann Y, Lipsker D, Monteil H, Sibilia J, Piemont Y (2000) Direct molecular typing of Borrelia burgdorferi sensu lato species in synovial samples from patients with lyme arthritis. J Clin Microbiol 38:1895-900. doi:10.1128/JCM.38.5.1895-1900.2000.
31. Grillon A, Scherlinger M, Boyer PH, De Martino S, Perdriger A, Blasquez A, Wipff J, Korganow AS, Bonnard C, Cantagrel A, Eyer D, Guerin F, Monteiro I, Woehl JM, Moreau P, Pennaforte JL, Lechevallier J, Bastides F, Colombey A, Imbert I, Maugars Y, Gicquel P, Cuchet F, Brax M, Sibilia J, Zilliox L, Barthel C, Arnaud L, Jaulhac B (2019) Characteristics and clinical outcomes after treatment of a national cohort of PCR-positive Lyme arthritis. Semin Arthritis Rheum 48:1105-1112. doi:10.1016/j.semarthrit.2018.09.007.
32. Gocko X, Lenormand C, Lemogne C, Bouiller K, Gehanno JF, Rabaud C, Perrot S, Eldin C, de Broucker T, Roblot F, Toubiana J, Sellal F, Vuillemet F, Sordet C, Fantin B, Lina G, Sobas C, Jaulhac B, Figoni J, Chirouze C, Hansmann Y, Hentgen V, Caumes E, Dieudonne M, Picone O, Bodaghi B, Gangneux JP, Degeilh B, Partouche H, Saunier A, Sotto A, Raffetin A, Monsuez JJ, Michel C, Boulanger N, Cathebras P, Tattevin P, endorsed by the following scientific s (2019) Lyme borreliosis and other tick-borne diseases. Guidelines from the French scientific societies. Med Mal Infect 49:296-317. doi:10.1016/j.medmal.2019.05.006.
33. Assous MV, Postic D, Paul G, Nevot P, Baranton G (1993) Western blot analysis of sera from Lyme borreliosis patients according to the genomic species of the Borrelia strains used as antigens. Eur J Clin Microbiol Infect Dis 12:261-8. doi:10.1007/BF01967256.
34. Bauvin O, Schmutz JL, De Martino S, Busato T, Cribier B, Barbaud A, Wahl D, Bursztejn AC (2017) A foot tumour as late cutaneous Lyme borreliosis: a new entity? Br J Dermatol 177:1127-1130. doi:10.1111/bjd.15633.
35. Del Giudice P, Reverte M, Giraudon E, Durant J, Ahmed Abdoulah S, Jaulhac B, Poirier JP (2022) First case of documented Lyme borreliosis in the Alpes-Maritimes department of South-Eastern France: Erythema chronicum migrans associated with Borrelia afzelii. Ann Dermatol Venereol 149:146-147. doi:10.1016/j.annder.2021.12.003.
36. Lacout A, Mas M, Pajaud J, Perronne V, Lequette Y, Franck M, Perronne C (2021) Real time micro-organisms PCR in 104 patients with polymorphic signs and symptoms that may be related to a tick bite. Eur J Microbiol Immunol (Bp) 11:62-75. doi:10.1556/1886.2021.00011.
37. Gallais F, De Martino SJ, Sauleau EA, Hansmann Y, Lipsker D, Lenormand C, Talagrand-Reboul E, Boyer PH, Boulanger N, Jaulhac B, Schramm F (2018) Multilocus sequence typing of clinical Borreliella afzelii strains: population structure and differential ability to disseminate in humans. Parasit Vectors 11:374. doi:10.1186/s13071-018-2938-x.
38. Lenormand C, Jaulhac B, Debarbieux S, Dupin N, Granel-Brocard F, Adamski H, Barthel C, Cribier B, Lipsker D (2016) Expanding the clinicopathological spectrum of late cutaneous Lyme borreliosis (acrodermatitis chronica atrophicans [ACA]): A prospective study of 20 culture- and/or polymerase chain reaction (PCR)-documented cases. J Am Acad Dermatol 74:685-92. doi:10.1016/j.jaad.2015.10.046.

39. Coipan EC, Jahfari S, Fonville M, Oei GA, Spanjaard L, Takumi K, Hovius JW, Sprong H (2016) Imbalanced presence of Borrelia burgdorferi s.l. multilocus sequence types in clinical manifestations of Lyme borreliosis. Infect Genet Evol 42:66-76. doi:10.1016/j.meegid.2016.04.019.
40. Jacquot M, Abrial D, Gasqui P, Bord S, Marsot M, Masseglia S, Pion A, Poux V, Zilliox L, Chapuis JL, Vourc'h G, Bailly X (2016) Multiple independent transmission cycles of a tick-borne pathogen within a local host community. Sci Rep 6:31273. doi:10.1038/srep31273.
41. Eiferman V, Guenno GL, Boiret-Dupre N, Barres B, Luciani L, Fournier PE (2022) Atypical Borrelia garinii infection in an immunocompromised patient mimicking high-grade lymphoma. Int J Infect Dis 121:102-104. doi:10.1016/j.ijid.2022.04.062.
42. Duffau P, Korbi S, Guillotin V, Talagrand-Reboul E, Menard A, Peuchant O (2022) An unexpected case of Borrelia garinii liver infection. Ann Clin Microbiol Antimicrob 21:15. doi:10.1186/s12941-022-00506-6.
43. Limbach FX, Jaulhac B, Puechal X, Monteil H, Kuntz JL, Piemont Y, Sibilia J (2001) Treatment resistant Lyme arthritis caused by Borrelia garinii. Ann Rheum Dis 60:284-6. doi:10.1136/ard.60.3.284.
44. Del Giudice P, Freychet F, Kopec L, Fenollar F, Eldin C, Velin M, Hubiche T, Raoult D, Mediannikov O (2023) Erythema Migrans Caused by Borrelia spielmanii, France. Emerg Infect Dis 29:2366-2369. doi:10.3201/eid2911.230149.
45. Calderaro A, Montecchini S, Gorrini C, Piccolo G, Chezzi C, Dettori G (2011) Presence of anti-Borrelia burgdorferi antibodies and Borrelia burgdorferi sensu lato DNA in samples of subjects in an area of the Northern Italy in the period 2002-2008. Diagn Microbiol Infect Dis 70:455-60. doi:10.1016/j.diagmicrobio.2010.09.007.
46. Santino I, Iori A, Sessa R, Sulli C, Favia G, Del Piano M (1998) Borrelia burgdorferi s.l. and Ehrlichia chaffeensis in the National Park of Abruzzo. FEMS Microbiol Lett 164:1-6. doi:10.1111/j.1574-6968.1998.tb13059.x.
47. Pauluzzi P, Bonin S, Gonzalez Inchaurraga MA, Stanta G, Trevisan G (2004) Detection of spirochaetal DNA simultaneously in skin biopsies, peripheral blood and urine from patients with erythema migrans. Acta Derm Venereol 84:106-10. doi:10.1080/00015550310006815.
48. Di Renzi S, Martini A, Binazzi A, Marinaccio A, Vonesch N, D'Amico W, Moro T, Fiorentini C, Ciufolini MG, Visca P, Tomao P (2010) Risk of acquiring tick-borne infections in forestry workers from Lazio, Italy. Eur J Clin Microbiol Infect Dis 29:1579-81. doi:10.1007/s10096-010-1028-6.
49. Stufano A, Iatta R, Sgroi G, Jahantigh HR, Cagnazzo F, Floel A, Lucchese G, Loconsole D, Centrone F, Mendoza-Roldan JA, Chironna M, Otranto D, Lovreglio P (2022) Seroprevalence of vector-borne pathogens in outdoor workers from southern Italy and associated occupational risk factors. Parasit Vectors 15:264. doi:10.1186/s13071-022-05385-6.
50. Stinco G, Ruscio M, Bergamo S, Trotter D, Patrone P (2014) Clinical features of 705 Borrelia burgdorferi seropositive patients in an endemic area of northern Italy. ScientificWorldJournal 2014:414505. doi:10.1155/2014/414505.
51. Santino I, Cammarata E, Franco S, Galdiero F, Oliva B, Sessa R, Cipriani P, Tempera G, Del Piano M (2004) Multicentric study of seroprevalence of Borrelia burgdorferi and Anaplasma phagocytophila in high-risk groups in regions of central and southern Italy. Int J Immunopathol Pharmacol 17:219-23. doi:10.1177/039463200401700214.

52. Santino I, Dastoli F, Lavorino C, Navazio M, Nicosia R, Oliveti A, Oliveti G, Pustorino R, Sessa R, Del Piano M (1996) Determination of antibodies to Borrelia burgdorferi in the serum of patients living in Calabria, southern Italy. Panminerva Med 38:167-72.
53. Cimmino MA, Azzolini A, Tobia F, Pesce CM (1989) Spirochetes in the spleen of a patient with chronic Lyme disease. Am J Clin Pathol 91:95-7. doi:10.1093/ajcp/91.1.95.
54. Beltrame A, Rodari P, Mauroner L, Zanella F, Moro L, Bertoli G, Da Re F, Russo F, Napoletano G, Silva R (2021) Emergence of Lyme borreliosis in the province of Verona, Northern Italy: Five-years of sentinel surveillance. Ticks Tick Borne Dis 12:101628. doi:10.1016/j.ttbdis.2020.101628.
55. Pugliese A, Beltramo T, Torre D (2007) Seroprevalence study of Tick-borne encephalitis, Borrelia burgdorferi, Dengue and Toscana virus in Turin Province. Cell Biochem Funct 25:185-8. doi:10.1002/cbf.1302.
56. Santino I, Sessa R, Di Pietro M, Del Piano M (2000) Lyme borreliosis in central Italy (1995-1998). New Microbiol 23:261-9.
57. Ciceroni L, Bartoloni A, Leoncini F, Ciarrocchi S, Pinto A, Favia G, Bartalesi F, Scagnoli L, Iori A (2003) Risk of tick-borne bacterial diseases in humans in the Florence area, Tuscany. Ann N Y Acad Sci 990:346-9. doi:10.1111/j.1749-6632.2003.tb07386.x.
58. Fumarola D, Marcuccio C, Crovato F, Nazzari G, Rovetta G, Cimmino MA, Bianchi G (1985) Lyme disease in Italy: first reported case. Boll Ist Sieroter Milan 64:483-5.
59. Milanese R, Marin M, Antonini-Canterin A, Crovatto M, Modolo ML, Scian I, Cilia AM, Santini GF (1991) Borreliosis risk after tick bite in north-eastern Italy. Microbiologica 14:257-9.
60. Trevisan G, Ruscio M, Cinco M, Nan K, Forgione P, Di Meo N, Tranchini P, Nacca M, Trincone S, Rimoldi SG, Giacomet V, Ricci M, Melandri D, Artioli S, Monteforte P, Stinco G, Bonin S (2023) The history of Lyme disease in Italy and its spread in the Italian territory. Front Pharmacol 14:1128142. doi:10.3389/fphar.2023.1128142.
61. Cinco M, De Giovannini R (1993) Protein and antigenic analysis of Borrelia burgdorferi isolated in northern Italy: computerized analysis of phenotypic characteristics. J Clin Microbiol 31:440-3. doi:10.1128/jcm.31.2.440-443.1993.
62. Ciceroni L, Ciarrochi S, Ciervo A, Mondarini V, Guzzo F, Caruso G, Murgia R, Cinco M (2001) Isolation and characterization of Borrelia burgdorferi sensu lato strains in an area of Italy where Lyme borreliosis is endemic. J Clin Microbiol 39:2254-60. doi:10.1128/JCM.39.6.2254-2260.2001.
63. Cinco M, Murgia R, Ruscio M, Andriolo B (1996) IgM and IgG significant reactivity to Borrelia burgdorferi sensu stricto, Borrelia garinii and Borrelia afzelii among Italian patients affected by Lyme arthritis or neuroborreliosis. FEMS Immunol Med Microbiol 14:159-66. doi:10.1111/j.1574-695X.1996.tb00283.x.
64. Tomasini C (2016) Cordoniform morphea: a clinicopathologic study of two cases presenting with the rope sign. J Cutan Pathol 43:613-622. doi:10.1111/cup.12704.
65. Cinco M, Murgia R, Costantini C (1995) Prevalence of IgG reactivity in Lyme borreliosis patients versus Borrelia garinii and Borrelia afzelii in a restricted area of Northern Italy. FEMS Immunol Med Microbiol 12:217-22. doi:10.1111/j.1574-695X.1995.tb00195.x.

66. Sonnleitner ST, Margos G, Wex F, Simeoni J, Zelger R, Schmutzhard E, Lass-Florl C, Walder G (2015) Human seroprevalence against Borrelia burgdorferi sensu lato in two comparable regions of the eastern Alps is not correlated to vector infection rates. Ticks Tick Borne Dis 6:221-7. doi:10.1016/j.ttbdis.2014.12.006.
67. Santino I, Berlutti F, Pantanella F, Sessa R, del Piano M (2008) Detection of Borrelia burgdorferi sensu lato DNA by PCR in serum of patients with clinical symptoms of Lyme borreliosis. FEMS Microbiol Lett 283:30-5. doi:10.1111/j.1574-6968.2008.01134.x.
68. Matera G, Labate A, Quirino A, Lamberti AG, Borz AG, Barreca GS, Mumoli L, Peronace C, Giancotti A, Gambardella A, Foc AA, Quattrone A (2014) Chronic neuroborreliosis by B. garinii: an unusual case presenting with epilepsy and multifocal brain MRI lesions. New Microbiol 37:393-7.
69. Ruzic-Sabljic E, Maraspin V, Lotric-Furlan S, Jurca T, Logar M, Pikelj-Pecnik A, Strle F (2002) Characterization of Borrelia burgdorferi sensu lato strains isolated from human material in Slovenia. Wien Klin Wochenschr 114:544-50.
70. Picken RN, Cheng Y, Strle F, Cimperman J, Maraspin V, Lotric-Furlan S, Ruzic-Sabljic E, Han D, Nelson JA, Picken MM, Trenholme GM (1996) Molecular characterization of Borrelia burgdorferi sensu lato from Slovenia revealing significant differences between tick and human isolates. Eur J Clin Microbiol Infect Dis 15:313-23. doi:10.1007/BF01695664.
71. Ruzic-Sabljic E, Strle F, Cimperman J, Maraspin V, Lotric-Furlan S, Pleterski-Rigler D (2000) Characterisation of Borrelia burgdorferi sensu lato strains isolated from patients with skin manifestations of Lyme borreliosis residing in Slovenia. J Med Microbiol 49:47-53. doi:10.1099/0022-1317-49-1-47.
72. Strle F, Lusa L, Ruzic-Sabljic E, Maraspin V, Lotric Furlan S, Cimperman J, Ogrinc K, Rojko T, Videcnik Zorman J, Stupica D (2013) Clinical characteristics associated with Borrelia burgdorferi sensu lato skin culture results in patients with erythema migrans. PLoS One 8:e82132. doi:10.1371/journal.pone.0082132.
73. Strle F, Ruzic-Sabljic E, Cimperman J, Lotric-Furlan S, Maraspin V (2006) Comparison of findings for patients with Borrelia garinii and Borrelia afzelii isolated from cerebrospinal fluid. Clin Infect Dis 43:704-10. doi:10.1086/506936.
74. Cizman M, Avsic-Zupanc T, Petrovec M, Ruzic-Sabljic E, Pokorn M (2000) Seroprevalence of ehrlichiosis, Lyme borreliosis and tick-borne encephalitis infections in children and young adults in Slovenia. Wien Klin Wochenschr 112:842-5.
75. Ruzic-Sabljic E, Zore A, Strle F (2008) Characterization of Borrelia burgdorferi sensu lato isolates by pulsed-field gel electrophoresis after MluI restriction of genomic DNA. Res Microbiol 159:441-8. doi:10.1016/j.resmic.2008.05.005.
76. Logar M, Ruzic-Sabljic E, Maraspin V, Lotric-Furlan S, Cimperman J, Jurca T, Strle F (2004) Comparison of erythema migrans caused by Borrelia afzelii and Borrelia garinii. Infection 32:15-9. doi:10.1007/s15010-004-3042-z.
77. Strle F, Nadelman RB, Cimperman J, Nowakowski J, Picken RN, Schwartz I, Maraspin V, Aguero-Rosenfeld ME, Varde S, Lotric-Furlan S, Wormser GP (1999) Comparison of culture-confirmed erythema migrans caused by Borrelia burgdorferi sensu stricto in New York State and by Borrelia afzelii in Slovenia. Ann Intern Med 130:32-6. doi:10.7326/0003-4819-130-1-199901050-00006.
78. Rojko T, Bogovic P, Lotric-Furlan S, Ogrinc K, Cerar-Kisek T, Glinsek Biskup U, Petrovec M, Ruzic-Sabljic E, Kastrin A, Strle F (2019) Borrelia burgdorferi sensu lato infection in patients with peripheral facial palsy. Ticks Tick Borne Dis 10:398-406. doi:10.1016/j.ttbdis.2018.11.019.

79. Arnez M, Ruzic-Sabljic E (2011) Borrelia burgdorferi sensu lato bacteremia in Slovenian children with solitary and multiple erythema migrans. Pediatr Infect Dis J 30:988-90. doi:10.1097/INF.0b013e318225b8c3.
80. Rozic M, Lah LL, Ruzic-Sabljic E, Kastrin A, Arnez M (2019) Lyme Neuroborreliosis in Children: Etiology and Comparison of Clinical Findings of Lyme Neuroborreliosis Caused by Borrelia garinii and Borrelia afzelii. Pediatr Infect Dis J 38:e279-e284. doi:10.1097/INF.0000000000002415.
81. Stupica D, Lusa L, Maraspin V, Bogovic P, Vidmar D, O'Rourke M, Traweger A, Livey I, Strle F (2015) Correlation of Culture Positivity, PCR Positivity, and Burden of Borrelia burgdorferi Sensu Lato in Skin Samples of Erythema Migrans Patients with Clinical Findings. PLoS One 10:e0136600. doi:10.1371/journal.pone.0136600.
82. Cerar T, Ruzic-Sabljic E, Glinsek U, Zore A, Strle F (2008) Comparison of PCR methods and culture for the detection of Borrelia spp. in patients with erythema migrans. Clin Microbiol Infect 14:653-8. doi:10.1111/j.1469-0691.2008.02013.x.
83. Maraspin V, Ruzic-Sabljic E, Cimperman J, Lotric-Furlan S, Jurca T, Picken RN, Strle F (2001) Isolation of Borrelia burgdorferi sensu lato from blood of patients with erythema migrans. Infection 29:65-70. doi:10.1007/s15010-001-0154-6.
84. Maraspin V, Ogrinc K, Rojko T, Bogovic P, Ruzic-Sabljic E, Kastrin A, Wormser GP, Strle F (2021) Characteristics of spirochetemic patients with a solitary erythema migrans skin lesion in Europe. PLoS One 16:e0250198. doi:10.1371/journal.pone.0250198.
85. Ruzic-Sabljic E, Arnez M, Logar M, Maraspin V, Lotric-Furlan S, Cimperman J, Strle F (2005) Comparison of Borrelia burgdorferi sensu lato strains isolated from specimens obtained simultaneously from two different sites of infection in individual patients. J Clin Microbiol 43:2194-200. doi:10.1128/JCM.43.5.2194-2200.2005.
86. Ruzic-Sabljic E, Arnez M, Lotric-Furlan S, Maraspin V, Cimperman J, Strle F (2001) Genotypic and phenotypic characterisation of Borrelia burgdorferi sensu lato strains isolated from human blood. J Med Microbiol 50:896-901. doi:10.1099/0022-1317-50-10-896.
87. Picken RN, Strle F, Ruzic-Sabljic E, Maraspin V, Lotric-Furlan S, Cimperman J, Cheng Y, Picken MM (1997) Molecular subtyping of Borrelia burgdorferi sensu lato isolates from five patients with solitary lymphocytoma. J Invest Dermatol 108:92-7. doi:10.1111/1523-1747.ep12285646.
88. Arnez M, Pleterski-Rigler D, Luznik-Bufon T, Ruzic-Sabljic E, Strle F (2003) Solitary and multiple erythema migrans in children: comparison of demographic, clinical and laboratory findings. Infection 31:404-9. doi:10.1007/s15010-003-4007-3.
89. Zore A, Ruzic-Sabljic E, Maraspin V, Cimperman J, Lotric-Furlan S, Pikelj A, Jurca T, Logar M, Strle F (2002) Sensitivity of culture and polymerase chain reaction for the etiologic diagnosis of erythema migrans. Wien Klin Wochenschr 114:606-9.
90. Strle F, Ruzic-Sabljic E, Logar M, Maraspin V, Lotric-Furlan S, Cimperman J, Ogrinc K, Stupica D, Nadelman RB, Nowakowski J, Wormser GP (2011) Comparison of erythema migrans caused by Borrelia burgdorferi and Borrelia garinii. Vector Borne Zoonotic Dis 11:1253-8. doi:10.1089/vbz.2010.0230.
91. Ogrinc K, Lusa L, Lotric-Furlan S, Bogovic P, Stupica D, Cerar T, Ruzic-Sabljic E, Strle F (2016) Course and Outcome of Early European Lyme Neuroborreliosis (Bannwarth Syndrome): Clinical and Laboratory Findings. Clin Infect Dis 63:346-53. doi:10.1093/cid/ciw299.

92. Becker NS, Rollins RE, Nosenko K, Paulus A, Martin S, Krebs S, Takano A, Sato K, Kovalev SY, Kawabata H, Fingerle V, Margos G (2020) High conservation combined with high plasticity: genomics and evolution of Borrelia bavariensis. BMC Genomics 21:702. doi:10.1186/s12864-020-07054-3.
93. Margos G, Vollmer SA, Cornet M, Garnier M, Fingerle V, Wilske B, Bormane A, Vitorino L, Collares-Pereira M, Drancourt M, Kurtenbach K (2009) A new Borrelia species defined by multilocus sequence analysis of housekeeping genes. Appl Environ Microbiol 75:5410-6. doi:10.1128/AEM.00116-09.
94. Gatzmann F, Metzler D, Krebs S, Blum H, Sing A, Takano A, Kawabata H, Fingerle V, Margos G, Becker NS (2015) NGS population genetics analyses reveal divergent evolution of a Lyme Borreliosis agent in Europe and Asia. Ticks Tick Borne Dis 6:344-51. doi:10.1016/j.ttbdis.2015.02.008.
95. Maraspin V, Ruzic-Sabljic E, Strle F (2006) Lyme borreliosis and Borrelia spielmanii. Emerg Infect Dis 12:1177. doi:10.3201/eid1207.060077.
96. Strle F (1999) Lyme borreliosis in Slovenia. Zentralbl Bakteriol 289:643-52. doi:10.1016/s0934-8840(99)80023-1.
97. Strle F, Picken RN, Cheng Y, Cimperman J, Maraspin V, Lotric-Furlan S, Ruzic-Sabljic E, Picken MM (1997) Clinical findings for patients with Lyme borreliosis caused by Borrelia burgdorferi sensu lato with genotypic and phenotypic similarities to strain 25015. Clin Infect Dis 25:273-80. doi:10.1086/514551.
98. Maraspin V, Nahtigal Klevisar M, Ruzic-Sabljic E, Lusa L, Strle F (2016) Borrelial Lymphocytoma in Adult Patients. Clin Infect Dis 63:914-21. doi:10.1093/cid/ciw417.
99. Tijsse-Klasen E, Sprong H, Pandak N (2013) Co-infection of Borrelia burgdorferi sensu lato and Rickettsia species in ticks and in an erythema migrans patient. Parasit Vectors 6:347. doi:10.1186/1756-3305-6-347.
100. Poljak I, Troselj-Vukic B, Miletic B, Morovic M, Ruzic-Sabljic E, Vucemilovic A, Materljan E (2000) Low sero-prevalence of Lyme borreliosis in the forested mountainous area of Gorski Kotar, Croatia. Croat Med J 41:433-6.
101. Habek M, Mubrin Z, Brinar VV (2007) Avellis syndrome due to borreliosis. Eur J Neurol 14:112-4. doi:10.1111/j.1468-1331.2006.01528.x.
102. Topolovec J, Puntaric D, Antolovic-Pozgain A, Vukovic D, Topolovec Z, Milas J, Drusko-Barisic V, Venus M (2003) Serologically detected "new" tick-borne zoonoses in eastern Croatia. Croat Med J 44:626-9.
103. Juric S, Janculjak D, Tomic S, Butkovic Soldo S, Bilic E (2014) Epileptic seizure as initial and only manifestation of neuroborreliosis: case report. Neurol Sci 35:793-4. doi:10.1007/s10072-014-1648-1.
104. Vukelic D, Bozinovic D, Morovic M, Tesovic G, Ruzic Sabljic E, Barisic N, Knezovic I (2000) Opsoclonus-myoclonus syndrome in a child with neuroborreliosis. J Infect 40:189-91. doi:10.1016/s0163-4453(00)80016-x.
105. Golubic D, Zember S (2001) Dual infection: tularemia and Lyme borreliosis acquired by single tick bite in northwest Croatia. Acta Med Croatica 55:207-9.
106. Golubic D, Rijpkema S, Tkalec-Makovec N, Ruzic E (1998) Epidemiologic, ecologic and clinical characteristics of Lyme borrelliosis in northwest Croatia. Acta Med Croatica 52:7-13.

107. Blazina K, Martinez I, Foro Znika M (2023) Bilateral facial nerve palsy as a presentation of coexisting neuroborreliosis and post-acute COVID-19 syndrome. Croat Med J 64:440-443. doi:10.3325/cmj.2023.64.440.
108. Burek V, Misic-Mayerus L, Maretic T (1992) Antibodies to Borrelia burgdorferi in various population groups in Croatia. Scand J Infect Dis 24:683-4. doi:10.3109/00365549209054658.
109. Situm M, Grahovac B, Markovic S, Lipozencic J, Poje G, Dobric I, Marinovic B, Bolanca-Bumber S, Misic-Majerus L (2000) Detection and genotyping of Borrelia burgdorferi sensu lato by polymerase chain reaction. Croat Med J 41:47-53.
110. Mulic R, Antonijevic S, Klismanic Z, Ropac D, Lucev O (2006) Epidemiological characteristics and clinical manifestations of Lyme borreliosis in Croatia. Mil Med 171:1105-9. doi:10.7205/milmed.171.11.1105.
111. Situm M, Poje G, Grahovac B, Marinovic B, Levanat S (2002) Diagnosis of Lyme borreliosis by polymerase chain reaction. Clin Dermatol 20:147-55. doi:10.1016/s0738-081x(01)00242-5.
112. Arapovic J, Skocibusic S, Grgic S, Nikolic J (2014) The first evidence of lyme neuroborreliosis in southern bosnia and herzegovina. Case Rep Infect Dis 2014:231969. doi:10.1155/2014/231969.
113. Myrseli T, Schönberg A, Simaku A, Çomo N, Pipero P, Bërxholi K, Bino S, Kraja D (2018) The current situation of Lyme borreliosis and the first seroepidemiology survey in dogs in Albania. Albanian Medical Journal 2.
114. Karageorgou I, Koutantou M, Papadogiannaki I, Voulgari-Kokota A, Makka S, Angelakis E (2022) Serological evidence of possible Borrelia afzelii lyme disease in Greece. New Microbes New Infect 46:100978. doi:10.1016/j.nmni.2022.100978.
115. Chatzipanagiotou S, Papandreou-Rakitzis P, Malamou-Ladas H, Antoniou P (1992) Determination of antibody titres for Borrelia burgdorferi in the serum of gipsies living in Attika, Greece. Eur J Clin Microbiol Infect Dis 11:477-8. doi:10.1007/BF01961871.
116. Antoniou M, Tselentis Y, Babalis T, Gikas A, Stratigakis N, Vlachonikolis I, Kafatos A, Fioretos M (1995) The seroprevalence of ten zoonoses in two villages of Crete, Greece. Eur J Epidemiol 11:415-23. doi:10.1007/BF01721226.
117. Stamouli M, Totos G, Braun HB, Michel G, Gizaris V (2000) Very low seroprevalence of Lyme borreliosis in young Greek males. Eur J Epidemiol 16:495-6. doi:10.1023/a:1007657430880.
118. Kalogeropoulos D, Asproudis I, Stefaniotou M, Moschos M, Gartzonika C, Bassukas I, Konitsiotis S, Milionis H, Gaitanis G, Malamos K, Kalogeropoulos C (2021) Spirochetal uveitis: Spectrum of clinical manifestations, diagnostic and therapeutic approach, final outcome and epidemiological data. Int Ophthalmol 41:4111-4126. doi:10.1007/s10792-021-01984-x.
119. Kouroupis D, Terzaki M, Moscha N, Sarvani A, Simoulidou E, Chatzimichailidou S, Giza E, Sapouridis G, Angelakis E, Petidis K, Pyrpasopoulou A (2024) Aseptic Meningitis Linked to Borrelia afzelii Seroconversion in Northeastern Greece: An Emerging Infectious Disease Contested in the Region. Trop Med Infect Dis 9. doi:10.3390/tropicalmed9010025.
120. Bucak O, Kocoglu ME, Tas T, Mengeloglu FZ (2016) Evaluation of Borrelia burgdorferi sensu lato seroprevalencein the province of Bolu, Turkey. Turk J Med Sci 46:727-32. doi:10.3906/sag-1504-100.

121. Akar N, Caliskan E, Ozturk CE, Ankarali H, Kilincel O, Oksuz S, Sahin I (2019) Seroprevalence of hantavirus and Borrelia burgdorferi in Duzce (Turkey) forest villages and the relationship with sociodemographic features. Turk J Med Sci 49:483-489. doi:10.3906/sag-1807-160.
122. Onal U, Aytac Erdem H, Uyan Onal A, Resat Sipahi O (2019) Systematic review of Lyme disease in Turkey. Trop Doct 49:165-170. doi:10.1177/0049475519843387.
123. Cikman A, Aydin M, Gulhan B, Karakecili F, Demirtas L, Kesik OA (2018) Geographical Features and Seroprevalence of Borrelia burgdorferi in Erzincan, Turkey. J Arthropod Borne Dis 12:378-386.
124. Gazi H, Ozkutuk N, Ecemis O, Atasoylu G, Koroglu G, Kurutepe S, Horasan GD (2016) Seroprevalence of West Nile virus, Crimean-Congo hemorrhagic fever virus, Francisella tularensis and Borrelia burgdorferi in rural population of Manisa, western Turkey. J Vector Borne Dis 53:112-7.
125. Kaya AD, Parlak AH, Ozturk CE, Behcet M (2008) Seroprevalence of Borrelia burgdorferi infection among forestry workers and farmers in Duzce, north-western Turkey. New Microbiol 31:203-9.
126. Cora M, Kaklikkaya N, Topbas M, Can G, Yavuzyilmaz A, Tosun I, Aydin F (2017) Determination of Seroprevalence of Borrelia burgdorferi IgG in Adult Population Living in Trabzon. Balkan Med J 34:47-52. doi:10.4274/balkanmedj.2015.0478.
127. Onen F, Tuncer D, Akar S, Birlik M, Akkoc N (2003) Seroprevalence of Borrelia burgdorferi in patients with Behcet's disease. Rheumatol Int 23:289-93. doi:10.1007/s00296-003-0313-4.
128. Ozcan Y, Gunes Takir S, Karagun E, Uyar B (2023) Dermatoscopic Features of Early Erythema Chronicum Migrans. Acta Dermatovenerol Croat 31:110-112.
129. Abraham Z, Feuerman EJ, Rozenbaum M, Gluck Z (1991) Lyme disease in Israel. J Am Acad Dermatol 25:729. doi:10.1016/s0190-9622(08)80681-0.
130. Holubar K (1992) Lyme disease in Israel. J Am Acad Dermatol 27:486-7. doi:10.1016/s0190-9622(08)80893-6.
131. Elhelw RA, El-Enbaawy MI, Samir A (2014) Lyme borreliosis: A neglected zoonosis in Egypt. Acta Trop 140:188-92. doi:10.1016/j.actatropica.2014.09.005.
132. Haberberger RL, Jr., Constantine NT, Schwan TG, Woody JN (1989) Lyme disease agent in Egypt? Trans R Soc Trop Med Hyg 83:556. doi:10.1016/0035-9203(89)90293-9.
133. Espi A, Del Cerro A, Somoano A, Garcia V, J MP, Barandika JF, Garcia-Perez AL (2017) Borrelia burgdorferi sensu lato prevalence and diversity in ticks and small mammals in a Lyme borreliosis endemic Nature Reserve in North-Western Spain. Incidence in surrounding human populations. Enferm Infecc Microbiol Clin 35:563-568. doi:10.1016/j.eimc.2016.06.011.
134. Gil H, Barral M, Escudero R, Garcia-Perez AL, Anda P (2005) Identification of a new Borrelia species among small mammals in areas of northern Spain where Lyme disease is endemic. Appl Environ Microbiol 71:1336-45. doi:10.1128/AEM.71.3.1336-1345.2005.
135. Delgado S, Carmenes P (1995) Seroepidemiological survey for Borrelia burgdorferi (Lyme disease) in dogs from northwestern of Spain. Eur J Epidemiol 11:321-4. doi:10.1007/BF01719437.

136. Miro G, Wright I, Michael H, Burton W, Hegarty E, Rodon J, Buch J, Pantchev N, von Samson-Himmelstjerna G (2022) Seropositivity of main vector-borne pathogens in dogs across Europe. Parasit Vectors 15:189. doi:10.1186/s13071-022-05316-5.
137. Solano-Gallego L, Llull J, Osso M, Hegarty B, Breitschwerdt E (2006) A serological study of exposure to arthropod-borne pathogens in dogs from northeastern Spain. Vet Res 37:231-44. doi:10.1051/vetres:2005054.
138. Pato FJ, Panadero R, Vazquez L, Lopez CM, Diaz P, Vazquez E, Diez-Banos P, Morrondo P, Fernandez G (2013) Seroprevalence of Borrelia burgdorferi sensu lato in roe deer (Capreolus capreolus) from northwestern Spain. J Zoo Wildl Med 44:660-5. doi:10.1638/2012-0240R2.1.
139. Sobrino R, Gortazar C (2008) Seroprevalence of antibodies to Borrelia burgdorferi in wild canids in Spain. Vet Rec 162:248-9. doi:10.1136/vr.162.8.248.
140. Ortuno A, Castella J, Marco I, Ruiz M, Lavin S (2003) Prevalence of antibodies to Borrelia burgdorferi sensu lato in southern chamois (Rupicapra pyrenaica) in Spain. J Vet Med B Infect Dis Vet Public Health 50:253-4. doi:10.1046/j.1439-0450.2003.00661.x.
141. Lledo L, Serrano JL, Isabel Gegundez M, Gimenez-Pardo C, Saz JV (2016) Antibodies to Rickettsia spp. and Borrelia burgdorferi in Spanish Wild Red Foxes (Vulpes vulpes). J Wildl Dis 52:122-5. doi:10.7589/2015-03-074.
142. Barandika JF, Hurtado A, Garcia-Esteban C, Gil H, Escudero R, Barral M, Jado I, Juste RA, Anda P, Garcia-Perez AL (2007) Tick-borne zoonotic bacteria in wild and domestic small mammals in northern Spain. Appl Environ Microbiol 73:6166-71. doi:10.1128/AEM.00590-07.
143. Diaz-Cao JM, Adaszek L, Dziegiel B, Paniagua J, Caballero-Gomez J, Winiarczyk S, Winiarczyk D, Cano-Terriza D, Garcia-Bocanegra I (2022) Prevalence of selected tick-borne pathogens in wild ungulates and ticks in southern Spain. Transbound Emerg Dis 69:1084-1094. doi:10.1111/tbed.14065.
144. Perez G, Bastian S, Chastagner A, Agoulon A, Plantard O, Vourc'h G, Butet A (2017) Ecological factors influencing small mammal infection by Anaplasma phagocytophilum and Borrelia burgdorferi s.l. in agricultural and forest landscapes. Environ Microbiol 19:4205-4219. doi:10.1111/1462-2920.13885.
145. Pantchev N, Schaper R, Limousin S, Norden N, Weise M, Lorentzen L (2009) Occurrence of Dirofilaria immitis and tick-borne infections caused by Anaplasma phagocytophilum, Borrelia burgdorferi sensu lato and Ehrlichia canis in domestic dogs in France: results of a countrywide serologic survey. Parasitol Res 105 Suppl 1:S101-14. doi:10.1007/s00436-009-1501-2.
146. Deruaz D, Eid P, Deruaz J, Sempere A, Bourgouin C, Rodhain F, Perez-Eid C (1996) Use of enzyme-labelled protein G assay for the detection of anti Borrelia burgdorferi antibodies in wild animal sera. Eur J Epidemiol 12:515-9. doi:10.1007/BF00144006.
147. Ollivier V, Choquet R, Gamble A, Bastien M, Combes B, Gilot-Fromont E, Pellerin M, Gaillard JM, Lemaitre JF, Verheyden H, Boulinier T (2023) Temporal dynamics of antibody level against Lyme disease bacteria in roe deer: Tale of a sentinel? Ecol Evol 13:e10414. doi:10.1002/ece3.10414.
148. Maurizi L, Marie JL, Aoun O, Courtin C, Gorsane S, Chal D, Davoust B (2010) Seroprevalence survey of equine Lyme borreliosis in France and in sub-Saharan Africa. Vector Borne Zoonotic Dis 10:535-7. doi:10.1089/vbz.2009.0083.
149. Marsot M, Chapuis JL, Gasqui P, Dozieres A, Masseglia S, Pisanu B, Ferquel E, Vourc'h G (2013) Introduced Siberian chipmunks (Tamias sibiricus barberi) contribute more to lyme borreliosis risk than native reservoir rodents. PLoS One 8:e55377. doi:10.1371/journal.pone.0055377.

150. Marsot M, Sigaud M, Chapuis JL, Ferquel E, Cornet M, Vourc'h G (2011) Introduced Siberian chipmunks (Tamias sibiricus barberi) harbor more-diverse Borrelia burgdorferi sensu lato genospecies than native bank voles (Myodes glareolus). Appl Environ Microbiol 77:5716-21. doi:10.1128/AEM.01846-10.
151. Vourc'h G, Marmet J, Chassagne M, Bord S, Chapuis JL (2007) Borrelia burgdorferi Sensu Lato in Siberian chipmunks (Tamias sibiricus) introduced in suburban forests in France. Vector Borne Zoonotic Dis 7:637-41. doi:10.1089/vbz.2007.0111.
152. Jacquot M, Bisseux M, Abrial D, Marsot M, Ferquel E, Chapuis JL, Vourc'h G, Bailly X (2014) High-throughput sequence typing reveals genetic differentiation and host specialization among populations of the Borrelia burgdorferi species complex that infect rodents. PLoS One 9:e88581. doi:10.1371/journal.pone.0088581.
153. Pisanu B, Chapuis JL, Dozieres A, Basset F, Poux V, Vourc'h G (2014) High prevalence of Borrelia burgdorferi s.l. in the European red squirrel Sciurus vulgaris in France. Ticks Tick Borne Dis 5:1-6. doi:10.1016/j.ttbdis.2013.07.007.
154. Pichon B, Gilot B, Perez-Eid C (2000) Detection of spirochaetes of Borrelia burgdorferi complexe in the skin of cervids by PCR and culture. Eur J Epidemiol 16:869-73. doi:10.1023/a:1007646216035.
155. Lebert I, Agoulon A, Bastian S, Butet A, Cargnelutti B, Cebe N, Chastagner A, Leger E, Lourtet B, Masseglia S, McCoy KD, Merlet J, Noel V, Perez G, Picot D, Pion A, Poux V, Rames JL, Rantier Y, Verheyden H, Vourc'h G, Plantard O (2020) Distribution of ticks, tick-borne pathogens and the associated local environmental factors including small mammals and livestock, in two French agricultural sites: the OSCAR database. Biodivers Data J 8:e50123. doi:10.3897/BDJ.8.e50123.
156. Cosson JF, Michelet L, Chotte J, Le Naour E, Cote M, Devillers E, Poulle ML, Huet D, Galan M, Geller J, Moutailler S, Vayssier-Taussat M (2014) Genetic characterization of the human relapsing fever spirochete Borrelia miyamotoi in vectors and animal reservoirs of Lyme disease spirochetes in France. Parasit Vectors 7:233. doi:10.1186/1756-3305-7-233.
157. Buffet JP, Marsot M, Vaumourin E, Gasqui P, Masseglia S, Marcheteau E, Huet D, Chapuis JL, Pisanu B, Ferquel E, Halos L, Vourc'h G, Vayssier-Taussat M (2012) Co-infection of Borrelia afzelii and Bartonella spp. in bank voles from a suburban forest. Comp Immunol Microbiol Infect Dis 35:583-9. doi:10.1016/j.cimid.2012.07.002.
158. Pascucci I, Di Domenico M, Dall'Acqua F, Sozio G, Camma C (2015) Detection of Lyme Disease and Q Fever Agents in Wild Rodents in Central Italy. Vector Borne Zoonotic Dis 15:404-11. doi:10.1089/vbz.2015.1807.
159. Ebani VV, Poli A, Rocchigiani G, Bertelloni F, Nardoni S, Papini RA, Mancianti F (2016) Serological survey on some pathogens in wild brown hares (Lepus europaeus) in Central Italy. Asian Pac J Trop Med 9:465-9. doi:10.1016/j.apjtm.2016.03.032.
160. Traversa D, Milillo P, Maggi R, Simonato G, Di Cesare A, Pezzuto C, Grillini M, Morelli S, Colombo M, Passarelli A, Grassano A, Serio P, Losurdo M, Brueckmann R (2023) Seroexposure to Zoonotic Anaplasma and Borrelia in Dogs and Horses That Are in Contact with Vulnerable People in Italy. Pathogens 12. doi:10.3390/pathogens12030470.

161. Petruccelli A, Ferrara G, Iovane G, Schettini R, Ciarcia R, Caputo V, Pompameo M, Pagnini U, Montagnaro S (2020) Seroprevalence of Ehrlichia spp., Anaplasma spp., Borrelia burgdorferi sensu lato, and Dirofilaria immitis in Stray Dogs, from 2016 to 2019, in Southern Italy. Animals (Basel) 11. doi:10.3390/ani11010009.
162. Ebani VV, Bertelloni F, Pinzauti P, Cerri D (2012) Seroprevalence of Leptospira spp. and Borrelia burgdorferi sensu lato in Italian horses. Ann Agric Environ Med 19:237-40.
163. Laus F, Veronesi F, Passamonti F, Paggi E, Cerquetella M, Hyatt D, Tesei B, Fioretti DP (2013) Prevalence of tick borne pathogens in horses from Italy. J Vet Med Sci 75:715-20. doi:10.1292/jvms.12-0449.
164. Mendoza-Roldan JA, Benelli G, Bezerra-Santos MA, Nguyen VL, Conte G, Iatta R, Furlanello T, Otranto D (2021) Seropositivity to canine tick-borne pathogens in a population of sick dogs in Italy. Parasit Vectors 14:292. doi:10.1186/s13071-021-04772-9.
165. Piantedosi D, Neola B, D'Alessio N, Di Prisco F, Santoro M, Pacifico L, Sgroi G, Auletta L, Buch J, Chandrashekar R, Breitschwerdt EB, Veneziano V (2017) Seroprevalence and risk factors associated with Ehrlichia canis, Anaplasma spp., Borrelia burgdorferi sensu lato, and D. immitis in hunting dogs from southern Italy. Parasitol Res 116:2651-2660. doi:10.1007/s00436-017-5574-z.
166. Ebani VV, Bertelloni F, Torracca B, Cerri D (2014) Serological survey of Borrelia burgdorferi sensu lato, Anaplasma phagocytophilum, and Ehrlichia canis infections in rural and urban dogs in Central Italy. Ann Agric Environ Med 21:671-5. doi:10.5604/12321966.1129912.
167. Galluzzo P, Grippi F, Di Bella S, Santangelo F, Sciortino S, Castiglia A, Sciacca C, Arnone M, Alduina R, Chiarenza G (2020) Seroprevalence of Borrelia burgdorferi in Stray Dogs from Southern Italy. Microorganisms 8. doi:10.3390/microorganisms8111688.
168. Morelli S, Gori F, Colombo M, Traversa D, Sarrocco G, Simonato G, Nespeca C, Di Cesare A, Frangipane di Regalbono A, Veronesi F, Russi I, Schnyder M (2021) Simultaneous Exposure to Angiostrongylus vasorum and Vector-Borne Pathogens in Dogs from Italy. Pathogens 10. doi:10.3390/pathogens10091200.
169. Colombo M, Morelli S, Simonato G, Di Cesare A, Veronesi F, Frangipane di Regalbono A, Grassi L, Russi I, Tiscar PG, Morganti G, Hattab J, Rizzo V, Traversa D (2021) Exposure to Major Vector-Borne Diseases in Dogs Subjected to Different Preventative Regimens in Endemic Areas of Italy. Pathogens 10. doi:10.3390/pathogens10050507.
170. Giudice E, Domina F, Britti D, Di Pietro S, Pugliese A (2003) Clinical findings associated with Borrelia burgdorferi infection in the dog. Vet Res Commun 27 Suppl 1:767-70. doi:10.1023/b:verc.0000014267.25428.32.
171. Ebani VV, Bertelloni F, Mani P (2017) Serological evidence of exposure to zoonotic tick-borne bacteria in pheasants (Phasianus colchicus). Ann Agric Environ Med 24:82-85. doi:10.5604/12321966.1234004.
172. Ebani VV, Bertelloni F, Mani P (2016) Molecular survey on zoonotic tick-borne bacteria and chlamydiae in feral pigeons (Columba livia domestica). Asian Pac J Trop Med 9:324-327. doi:10.1016/j.apjtm.2016.03.005.
173. Ebani VV, Rocchigiani G, Bertelloni F, Nardoni S, Leoni A, Nicoloso S, Mancianti F (2016) Molecular survey on the presence of zoonotic arthropod-borne pathogens in wild red deer (Cervus elaphus). Comp Immunol Microbiol Infect Dis 47:77-80. doi:10.1016/j.cimid.2016.06.003.

174. Cuteri V, Diverio S, Carnieletto P, Turilli C, Valente C (1999) Serological survey for antibodies against selected infectious agents among fallow deer (Dama dama) in central Italy. Zentralbl Veterinarmed B 46:545-9. doi:10.1111/j.1439-0450.1999.tb01247.x.
175. Grassi L, Drigo M, Zelena H, Pasotto D, Cassini R, Mondin A, Franzo G, Tucciarone CM, Ossola M, Vidorin E, Menandro ML (2023) Wild ungulates as sentinels of flaviviruses and tick-borne zoonotic pathogen circulation: an Italian perspective. BMC Vet Res 19:155. doi:10.1186/s12917-023-03717-x.
176. Ebani VV, Guardone L, Rocchigiani G, Bascherini A, Cagnoli G, Bertelloni F, Bongi P, Russo C, Riccioli F, Mancianti F (2022) Molecular survey on the presence of arthropod-borne bacteria and protozoans in roe deer (Capreolus capreolus) and ticks from Central Italy. Acta Trop 233:106586. doi:10.1016/j.actatropica.2022.106586.
177. Ciceroni L, Simeoni J, Pacetti AI, Ciarrocchi S, Cacciapuoti B (1996) Antibodies to Borrelia burgdorferi in sheep and goats. Alto Adige-South Tyrol, Italy. New Microbiol 19:171-4.
178. Sgroi G, Iatta R, Veneziano V, Bezerra-Santos MA, Lesiczka P, Hrazdilova K, Annoscia G, D'Alessio N, Golovchenko M, Rudenko N, Modry D, Otranto D (2021) Molecular survey on tick-borne pathogens and Leishmania infantum in red foxes (Vulpes vulpes) from southern Italy. Ticks Tick Borne Dis 12:101669. doi:10.1016/j.ttbdis.2021.101669.
179. Martello E, Mannelli A, Grego E, Ceballos LA, Ragagli C, Stella MC, Tomassone L (2019) Borrelia burgdorferi sensu lato and spotted fever group rickettsiae in small rodents and attached ticks in the Northern Apennines, Italy. Ticks Tick Borne Dis 10:862-867. doi:10.1016/j.ttbdis.2019.04.005.
180. Veronesi F, Laus F, Passamonti F, Tesei B, Piergili Fioretti D, Genchi C (2012) Occurrence of Borrelia lusitaniae infection in horses. Vet Microbiol 160:535-8. doi:10.1016/j.vetmic.2012.06.029.
181. Mendoza-Roldan JA, Colella V, Lia RP, Nguyen VL, Barros-Battesti DM, Iatta R, Dantas-Torres F, Otranto D (2019) Borrelia burgdorferi (sensu lato) in ectoparasites and reptiles in southern Italy. Parasit Vectors 12:35. doi:10.1186/s13071-019-3286-1.
182. Zore A, Petrovec M, Prosenc K, Trilar T, Ruzic-Sabljic E, Avsic-Zupanc T (1999) Infection of small mammals with Borrelia burgdorferi sensu lato in Slovenia as determined by polymerase chain reaction (PCR). Wien Klin Wochenschr 111:997-9.
183. Cerar T, Korva M, Avsic-Zupanc T, Ruzic-Sabljic E (2015) Detection, identification and genotyping of Borrellia spp. in rodents in Slovenia by PCR and culture. BMC Vet Res 11:188. doi:10.1186/s12917-015-0501-y.
184. Mrljak V, Kules J, Mihaljevic Z, Torti M, Gotic J, Crnogaj M, Zivicnjak T, Mayer I, Smit I, Bhide M, Baric Rafaj R (2017) Prevalence and Geographic Distribution of Vector-Borne Pathogens in Apparently Healthy Dogs in Croatia. Vector Borne Zoonotic Dis 17:398-408. doi:10.1089/vbz.2016.1990.
185. Jurkovic D, Beck A, Huber D, Mihaljevic Z, Polkinghorne A, Martinkovic F, Lukacevic D, Pilat M, Brezak R, Bosnic S, Beck R (2019) Seroprevalence of vector-borne pathogens in dogs from Croatia. Parasitol Res 118:347-352. doi:10.1007/s00436-018-6129-7.
186. Tadin A, Tokarz R, Markotic A, Margaletic J, Turk N, Habus J, Svoboda P, Vucelja M, Desai A, Jain K, Lipkin WI (2016) Molecular Survey of Zoonotic Agents in Rodents and Other Small Mammals in Croatia. Am J Trop Med Hyg 94:466-73. doi:10.4269/ajtmh.15-0517.
187. Angelou A, Gelasakis AI, Verde N, Pantchev N, Schaper R, Chandrashekar R, Papadopoulos E (2019) Prevalence and risk factors for selected canine vector-borne diseases in Greece. Parasit Vectors 12:283. doi:10.1186/s13071-019-3543-3.

188. Athanasiou LV, Kontos VI, Kritsepi Konstantinou M, Polizopoulou ZS, Rousou XA, Christodoulopoulos G (2019) Cross-Sectional Serosurvey and Factors Associated with Exposure of Dogs to Vector-Borne Pathogens in Greece. Vector Borne Zoonotic Dis 19:923-928. doi:10.1089/vbz.2019.2471.
189. Athanasiou LV, Spanou VM, Katsogiannou EG, Katsoulos PD (2021) Hematological Features in Sheep with IgG and IgM Antibodies against Borrelia burgdorferi sensu lato. Pathogens 10. doi:10.3390/pathogens10020164.
190. Athanasiou LV, Tsokana CN, Gougoulis DA, Tzivara AH, Dedousi A, Katsoulos PD (2023) Natural Co-Exposure to Borrelia burgdorferi s.l. and Anaplasma phagocytophilum: Unraveling the Hematological Profile in Sheep. Life (Basel) 13. doi:10.3390/life13020469.
191. Athanasiou LV, Katsogiannou EG, Tyrnenopoulou P, Gougoulis D, Apostolidis KN, Papadakis SM, Kokkinaki KCG, Papatsiros VG, Tsokana CN (2023) Evidence of Horse Exposure to Anaplasma phagocytophilum, Borrelia burgdorferi, and Leishmania infantum in Greece through the Detection of IgG Antibodies in Serum and in an Alternative Diagnostic Sample-The Saliva. Biomolecules 13. doi:10.3390/biom13091374.
192. Bhide M, Yilmaz Z, Golcu E, Torun S, Mikula I (2008) Seroprevalence of anti-Borrelia burgdorferi antibodies in dogs and horses in Turkey. Ann Agric Environ Med 15:85-90.
193. Muz MN, Erat S, Mumcuoglu KY (2021) Protozoan and Microbial Pathogens of House Cats in the Province of Tekirdag in Western Turkey. Pathogens 10. doi:10.3390/pathogens10091114.
194. Guner ES, Watanabe M, Kadosaka T, Polat E, Gargili A, Gulanber A, Ohashi N, Kaneda K, Imai Y, Masuzawa T (2005) Seroepidemiology of Borrelia burgdorferi sensu lato and Anaplasma phagocytophilum in wild mice captured in northern Turkey. Epidemiol Infect 133:331-6. doi:10.1017/s0950268804003309.
195. Selim A, Alanazi AD, Sazmand A, Otranto D (2021) Seroprevalence and associated risk factors for vector-borne pathogens in dogs from Egypt. Parasit Vectors 14:175. doi:10.1186/s13071-021-04670-0.
196. Elhelw R, Elhariri M, Hamza D, Abuowarda M, Ismael E, Farag H (2021) Evidence of the presence of Borrelia burgdorferi in dogs and associated ticks in Egypt. BMC Vet Res 17:49. doi:10.1186/s12917-020-02733-5.
197. El-Alfy ES, Abbas I, Baghdadi HB, El-Sayed SAE, Ji S, Rizk MA (2022) Molecular Epidemiology and Species Diversity of Tick-Borne Pathogens of Animals in Egypt: A Systematic Review and Meta-Analysis. Pathogens 11. doi:10.3390/pathogens11080912.
198. Ashour R, Hamza D, Kadry M, Sabry MA (2023) The Surveillance of Borrelia Species in Camelus dromedarius and Associated Ticks: The First Detection of Borrelia miyamotoi in Egypt. Vet Sci 10. doi:10.3390/vetsci10020141.
199. Ben Said M, Belkahia H, Alberti A, Abdi K, Zhioua M, Daaloul-Jedidi M, Messadi L (2016) First molecular evidence of [i]Borrelia burgdorferi[/i] sensu lato in goats, sheep, cattle and camels in Tunisia. Ann Agric Environ Med 23:442-7. doi:10.5604/12321966.1219184.
200. Dsouli N, Younsi-Kabachii H, Postic D, Nouira S, Gern L, Bouattour A (2006) Reservoir role of lizard Psammodromus algirus in transmission cycle of Borrelia burgdorferi sensu lato (Spirochaetaceae) in Tunisia. J Med Entomol 43:737-42. doi:10.1603/0022-2585(2006)43[737:rrolpa]2.0.co;2.
201. Azzag N, Petit E, Gandoin C, Bouillin C, Ghalmi F, Haddad N, Boulouis HJ (2015) Prevalence of select vector-borne pathogens in stray and client-owned dogs from Algiers. Comp Immunol Microbiol Infect Dis 38:1-7. doi:10.1016/j.cimid.2015.01.001.

202. Laamari A, Azzag N, Tennah S, Derdour SY, China B, Bouabdallah R, Ghalmi F (2020) Seroprevalence of Antibodies Against Anaplasma Phagocytophilum and Borrelia Burgdorferi in Horses (Equus Caballus) from Northern Algeria. J Vet Res 64:413-419. doi:10.2478/jvetres-2020-0045.
203. Del Cerro A, Oleaga A, Somoano A, Barandika JF, Garcia-Perez AL, Espi A (2022) Molecular identification of tick-borne pathogens (Rickettsia spp., Anaplasma phagocytophilum, Borrelia burgdorferi sensu lato, Coxiella burnetii and piroplasms) in questing and feeding hard ticks from North-Western Spain. Ticks Tick Borne Dis 13:101961. doi:10.1016/j.ttbdis.2022.101961.
204. Ortega N, Arcenillas-Hernandez I, Villa MI, Gonzalez MD, Caro MR (2024) Molecular identification of Borrelia and SFG Rickettsia spp. in hard ticks parasitizing domestic and wild animals in southeastern Spain. Vet Res Commun doi:10.1007/s11259-023-10292-x. doi:10.1007/s11259-023-10292-x.
205. Remesar S, Diaz P, Venzal JM, Prieto A, Estrada-Pena A, Lopez CM, Panadero R, Fernandez G, Diez-Banos P, Morrondo P (2019) Longitudinal Study of Infection with Borrelia spp. in Questing Ticks from North-Western Spain. Vector Borne Zoonotic Dis 19:785-792. doi:10.1089/vbz.2019.2442.
206. Palomar AM, Portillo A, Santibanez P, Mazuelas D, Roncero L, Gutierrez O, Oteo JA (2017) Presence of Borrelia turdi and Borrelia valaisiana (Spirochaetales: Spirochaetaceae) in Ticks Removed From Birds in the North of Spain, 2009-2011. J Med Entomol 54:243-246. doi:10.1093/jme/tjw158.
207. Diaz P, Arnal JL, Remesar S, Perez-Creo A, Venzal JM, Vazquez-Lopez ME, Prieto A, Fernandez G, Lopez CM, Panadero R, Benito A, Diez-Banos P, Morrondo P (2017) Molecular identification of Borrelia spirochetes in questing Ixodes ricinus from northwestern Spain. Parasit Vectors 10:615. doi:10.1186/s13071-017-2574-x.
208. Palomar AM, Portillo A, Santibanez P, Santibanez S, Oteo JA (2018) Borrelia miyamotoi: Should this pathogen be considered for the diagnosis of tick-borne infectious diseases in Spain? Enferm Infecc Microbiol Clin (Engl Ed) 36:568-571. doi:10.1016/j.eimc.2017.10.020.
209. Estrada-Pena A, Oteo JA, Estrada-Pena R, Gortazar C, Osacar JJ, Moreno JA, Castella J (1995) Borrelia burgdorferi sensu lato in ticks (Acari: Ixodidae) from two different foci in Spain. Exp Appl Acarol 19:173-80. doi:10.1007/BF00046289.
210. Diaz P, Remesar S, Venzal JM, Vazquez-Lopez ME, Fernandez G, Lopez C, Diez-Banos P, Morrondo P, Panadero R (2019) Occurrence of Borrelia and Borreliella species in Ixodes ricinus collected from roe deer in northwestern Spain. Med Vet Entomol 33:427-430. doi:10.1111/mve.12364.
211. Estrada-Pena A, Roura X, Sainz A, Miro G, Solano-Gallego L (2017) Species of ticks and carried pathogens in owned dogs in Spain: Results of a one-year national survey. Ticks Tick Borne Dis 8:443-452. doi:10.1016/j.ttbdis.2017.02.001.
212. Barral M, Garcia-Perez AL, Juste RA, Hurtado A, Escudero R, Sellek RE, Anda P (2002) Distribution of Borrelia burgdorferi sensu lato in Ixodes ricinus (Acari: Ixodidae) ticks from the Basque Country, Spain. J Med Entomol 39:177-84. doi:10.1603/0022-2585-39.1.177.
213. Barandika JF, Hurtado A, Garcia-Sanmartin J, Juste RA, Anda P, Garcia-Perez AL (2008) Prevalence of tick-borne zoonotic bacteria in questing adult ticks from northern Spain. Vector Borne Zoonotic Dis 8:829-35. doi:10.1089/vbz.2008.0023.
214. Toledo A, Olmeda AS, Escudero R, Jado I, Valcarcel F, Casado-Nistal MA, Rodriguez-Vargas M, Gil H, Anda P (2009) Tick-borne zoonotic bacteria in ticks collected from central Spain. Am J Trop Med Hyg 81:67-74.

215. Remesar S, Matute R, Diaz P, Martinez-Calabuig N, Prieto A, Diaz-Cao JM, Lopez-Lorenzo G, Fernandez G, Lopez C, Panadero R, Diez-Banos P, Morrondo P, Garcia-Dios D (2023) Tick-borne pathogens in ticks from urban and suburban areas of north-western Spain: Importance of Ixodes frontalis harbouring zoonotic pathogens. Med Vet Entomol doi:10.1111/mve.12648. doi:10.1111/mve.12648.
216. Delgado JD, Abreu-Yanes E, Abreu-Acosta N, Flor MD, Foronda P (2017) Vertebrate Ticks Distribution and Their Role as Vectors in Relation to Road Edges and Underpasses. Vector Borne Zoonotic Dis 17:376-383. doi:10.1089/vbz.2016.2073.
217. Palomar AM, Santibanez P, Mazuelas D, Roncero L, Santibanez S, Portillo A, Oteo JA (2012) Role of birds in dispersal of etiologic agents of tick-borne zoonoses, Spain, 2009. Emerg Infect Dis 18:1188-91. doi:10.3201/eid1807.111777.
218. Merino FJ, Nebreda T, Serrano JL, Fernandez-Soto P, Encinas A, Perez-Sanchez R (2005) Tick species and tick-borne infections identified in population from a rural area of Spain. Epidemiol Infect 133:943-9. doi:10.1017/S0950268805004061.
219. Estrada-Pena A, Osacar JJ, Pichon B, Gray JS (2005) Hosts and pathogen detection for immature stages of Ixodes ricinus (Acari: Ixodidae) in North-Central Spain. Exp Appl Acarol 37:257-68. doi:10.1007/s10493-005-3271-6.
220. Ruiz-Fons F, Fernandez-de-Mera IG, Acevedo P, Gortazar C, de la Fuente J (2012) Factors driving the abundance of ixodes ricinus ticks and the prevalence of zoonotic I. ricinus-borne pathogens in natural foci. Appl Environ Microbiol 78:2669-76. doi:10.1128/AEM.06564-11.
221. Nebbak A, Dahmana H, Almeras L, Raoult D, Boulanger N, Jaulhac B, Mediannikov O, Parola P (2019) Co-infection of bacteria and protozoan parasites in Ixodes ricinus nymphs collected in the Alsace region, France. Ticks Tick Borne Dis 10:101241. doi:10.1016/j.ttbdis.2019.06.001.
222. Boyer PH, Boulanger N, Nebbak A, Collin E, Jaulhac B, Almeras L (2017) Assessment of MALDI-TOF MS biotyping for Borrelia burgdorferi sl detection in Ixodes ricinus. PLoS One 12:e0185430. doi:10.1371/journal.pone.0185430.
223. Geurden T, Becskei C, Six RH, Maeder S, Latrofa MS, Otranto D, Farkas R (2018) Detection of tick-borne pathogens in ticks from dogs and cats in different European countries. Ticks Tick Borne Dis 9:1431-1436. doi:10.1016/j.ttbdis.2018.06.013.
224. Michelet L, Joncour G, Devillers E, Torina A, Vayssier-Taussat M, Bonnet SI, Moutailler S (2016) Tick species, tick-borne pathogens and symbionts in an insular environment off the coast of Western France. Ticks Tick Borne Dis 7:1109-1115. doi:10.1016/j.ttbdis.2016.08.014.
225. Jumpertz M, Sevestre J, Luciani L, Houhamdi L, Fournier PE, Parola P (2023) Bacterial Agents Detected in 418 Ticks Removed from Humans during 2014-2021, France. Emerg Infect Dis 29:701-710. doi:10.3201/eid2904.221572.
226. Socolovschi C, Reynaud P, Kernif T, Raoult D, Parola P (2012) Rickettsiae of spotted fever group, Borrelia valaisiana, and Coxiella burnetii in ticks on passerine birds and mammals from the Camargue in the south of France. Ticks Tick Borne Dis 3:355-60. doi:10.1016/j.ttbdis.2012.10.019.
227. Halos L, Jamal T, Maillard R, Beugnet F, Le Menach A, Boulouis HJ, Vayssier-Taussat M (2005) Evidence of Bartonella sp. in questing adult and nymphal Ixodes ricinus ticks from France and co-infection with Borrelia burgdorferi sensu lato and Babesia sp. Vet Res 36:79-87. doi:10.1051/vetres:2004052.
228. Halos L, Vourc'h G, Cotte V, Gasqui P, Barnouin J, Boulous HJ, Vayssier-Taussat M (2006) Prevalence of Anaplasma phagocytophilum, Rickettsia sp. and Borrelia burgdorferi sensu lato DNA in questing Ixodes ricinus ticks from France. Ann N Y Acad Sci 1078:316-9. doi:10.1196/annals.1374.059.

229. Marchant A, Le Coupanec A, Joly C, Perthame E, Sertour N, Garnier M, Godard V, Ferquel E, Choumet V (2017) Infection of Ixodes ricinus by Borrelia burgdorferi sensu lato in peri-urban forests of France. PLoS One 12:e0183543. doi:10.1371/journal.pone.0183543.
230. Ferquel E, Garnier M, Marie J, Bernede-Bauduin C, Baranton G, Perez-Eid C, Postic D (2006) Prevalence of Borrelia burgdorferi sensu lato and Anaplasmataceae members in Ixodes ricinus ticks in Alsace, a focus of Lyme borreliosis endemicity in France. Appl Environ Microbiol 72:3074-8. doi:10.1128/AEM.72.4.3074-3078.2006.
231. Cotte V, Bonnet S, Cote M, Vayssier-Taussat M (2010) Prevalence of five pathogenic agents in questing Ixodes ricinus ticks from western France. Vector Borne Zoonotic Dis 10:723-30. doi:10.1089/vbz.2009.0066.
232. Vayssier-Taussat M, Moutailler S, Michelet L, Devillers E, Bonnet S, Cheval J, Hebert C, Eloit M (2013) Next generation sequencing uncovers unexpected bacterial pathogens in ticks in western Europe. PLoS One 8:e81439. doi:10.1371/journal.pone.0081439.
233. Bonnet S, de la Fuente J, Nicollet P, Liu X, Madani N, Blanchard B, Maingourd C, Alongi A, Torina A, Fernandez de Mera IG, Vicente J, George JC, Vayssier-Taussat M, Joncour G (2013) Prevalence of tick-borne pathogens in adult Dermacentor spp. ticks from nine collection sites in France. Vector Borne Zoonotic Dis 13:226-36. doi:10.1089/vbz.2011.0933.
234. Quessada T, Martial-Convert F, Arnaud S, Leudet De La Vallee H, Gilot B, Pichot J (2003) Prevalence of Borrelia burgdorferi species and identification of Borrelia valaisiana in questing Ixodes ricinus in the Lyon region of France as determined by polymerase chain reaction-restriction fragment length polymorphism. Eur J Clin Microbiol Infect Dis 22:165-73. doi:10.1007/s10096-002-0866-2.
235. Gilot B, Degeilh B, Pichot J, Doche B, Guiguen C (1996) Prevalence of Borrelia burgdorferi (sensu lato) in Ixodes ricinus (L.) populations in France, according to a phytoecological zoning of the territory. Eur J Epidemiol 12:395-401. doi:10.1007/BF00145304.
236. Reis C, Cote M, Paul RE, Bonnet S (2011) Questing ticks in suburban forest are infected by at least six tick-borne pathogens. Vector Borne Zoonotic Dis 11:907-16. doi:10.1089/vbz.2010.0103.
237. Sevestre J, Diarra AZ, Oumarou HA, Durant J, Delaunay P, Parola P (2021) Detection of emerging tick-borne disease agents in the Alpes-Maritimes region, southeastern France. Ticks Tick Borne Dis 12:101800. doi:10.1016/j.ttbdis.2021.101800.
238. Grech-Angelini S, Stachurski F, Vayssier-Taussat M, Devillers E, Casabianca F, Lancelot R, Uilenberg G, Moutailler S (2020) Tick-borne pathogens in ticks (Acari: Ixodidae) collected from various domestic and wild hosts in Corsica (France), a Mediterranean island environment. Transbound Emerg Dis 67:745-757. doi:10.1111/tbed.13393.
239. Moutailler S, Valiente Moro C, Vaumourin E, Michelet L, Tran FH, Devillers E, Cosson JF, Gasqui P, Van VT, Mavingui P, Vourc'h G, Vayssier-Taussat M (2016) Co-infection of Ticks: The Rule Rather Than the Exception. PLoS Negl Trop Dis 10:e0004539. doi:10.1371/journal.pntd.0004539.
240. Aubry C, Socolovschi C, Raoult D, Parola P (2016) Bacterial agents in 248 ticks removed from people from 2002 to 2013. Ticks Tick Borne Dis 7:475-81. doi:10.1016/j.ttbdis.2016.02.003.
241. Zhioua E, Postic D, Rodhain F, Perez-Eid C (1996) Infection of Ixodes ricinus (Acari:Ixodidae) by Borrelia burgdorferi in Ile de France. J Med Entomol 33:694-7. doi:10.1093/jmedent/33.4.694.

242. Beytout J, George JC, Malaval J, Garnier M, Beytout M, Baranton G, Ferquel E, Postic D (2007) Lyme borreliosis incidence in two French departments: correlation with infection of Ixodes ricinus ticks by Borrelia burgdorferi sensu lato. Vector Borne Zoonotic Dis 7:507-17. doi:10.1089/vbz.2006.0633.
243. Richter D, Schlee DB, Matuschka FR (2003) Relapsing fever-like spirochetes infecting European vector tick of Lyme disease agent. Emerg Infect Dis 9:697-701. doi:10.3201/eid0906.020459.
244. Rataud A, Galon C, Bournez L, Henry PY, Marsot M, Moutailler S (2022) Diversity of Tick-Borne Pathogens in Tick Larvae Feeding on Breeding Birds in France. Pathogens 11. doi:10.3390/pathogens11080946.
245. Cicculli V, Capai L, Quilichini Y, Masse S, Fernandez-Alvarez A, Minodier L, Bompard P, Charrel R, Falchi A (2019) Molecular investigation of tick-borne pathogens in ixodid ticks infesting domestic animals (cattle and sheep) and small rodents (black rats) of Corsica, France. Ticks Tick Borne Dis 10:606-613. doi:10.1016/j.ttbdis.2019.02.007.
246. Vourc'h G, Abrial D, Bord S, Jacquot M, Masseglia S, Poux V, Pisanu B, Bailly X, Chapuis JL (2016) Mapping human risk of infection with Borrelia burgdorferi sensu lato, the agent of Lyme borreliosis, in a periurban forest in France. Ticks Tick Borne Dis 7:644-652. doi:10.1016/j.ttbdis.2016.02.008.
247. Lejal E, Moutailler S, Simo L, Vayssier-Taussat M, Pollet T (2019) Tick-borne pathogen detection in midgut and salivary glands of adult Ixodes ricinus. Parasit Vectors 12:152. doi:10.1186/s13071-019-3418-7.
248. Akl T, Bourgoin G, Souq ML, Appolinaire J, Poirel MT, Gibert P, Abi Rizk G, Garel M, Zenner L (2019) Detection of tick-borne pathogens in questing Ixodes ricinus in the French Pyrenees and first identification of Rickettsia monacensis in France. Parasite 26:20. doi:10.1051/parasite/2019019.
249. Boyer PH, Barthel C, Mohseni-Zadeh M, Talagrand-Reboul E, Frickert M, Jaulhac B, Boulanger N (2022) Impact of Different Anthropogenic Environments on Ticks and Tick-Associated Pathogens in Alsace, a French Region Highly Endemic for Tick-Borne Diseases. Microorganisms 10. doi:10.3390/microorganisms10020245.
250. Bonnet SI, Paul RE, Bischoff E, Cote M, Le Naour E (2017) First identification of Rickettsia helvetica in questing ticks from a French Northern Brittany Forest. PLoS Negl Trop Dis 11:e0005416. doi:10.1371/journal.pntd.0005416.
251. Pichon B, Mousson L, Figureau C, Rodhain F, Perez-Eid C (1999) Density of deer in relation to the prevalence of Borrelia burgdorferi s.l. in Ixodes ricinus nymphs in Rambouillet forest, France. Exp Appl Acarol 23:267-75. doi:10.1023/a:1006023115617.
252. Lejal E, Marsot M, Chalvet-Monfray K, Cosson JF, Moutailler S, Vayssier-Taussat M, Pollet T (2019) A three-years assessment of Ixodes ricinus-borne pathogens in a French peri-urban forest. Parasit Vectors 12:551. doi:10.1186/s13071-019-3799-7.
253. Jacquot M, Gonnet M, Ferquel E, Abrial D, Claude A, Gasqui P, Choumet V, Charras-Garrido M, Garnier M, Faure B, Sertour N, Dorr N, De Goer J, Vourc'h G, Bailly X (2014) Comparative population genomics of the Borrelia burgdorferi species complex reveals high degree of genetic isolation among species and underscores benefits and constraints to studying intra-specific epidemiological processes. PLoS One 9:e94384. doi:10.1371/journal.pone.0094384.

254. Halos L, Bord S, Cotte V, Gasqui P, Abrial D, Barnouin J, Boulouis HJ, Vayssier-Taussat M, Vourc'h G (2010) Ecological factors characterizing the prevalence of bacterial tick-borne pathogens in Ixodes ricinus ticks in pastures and woodlands. Appl Environ Microbiol 76:4413-20. doi:10.1128/AEM.00610-10.
255. Yssouf A, Flaudrops C, Drali R, Kernif T, Socolovschi C, Berenger JM, Raoult D, Parola P (2013) Matrix-assisted laser desorption ionization-time of flight mass spectrometry for rapid identification of tick vectors. J Clin Microbiol 51:522-8. doi:10.1128/JCM.02665-12.
256. Paul RE, Cote M, Le Naour E, Bonnet SI (2016) Environmental factors influencing tick densities over seven years in a French suburban forest. Parasit Vectors 9:309. doi:10.1186/s13071-016-1591-5.
257. Saint Girons I, Gern L, Gray JS, Guy EC, Korenberg E, Nuttall PA, Rijpkema SG, Schonberg A, Stanek G, Postic D (1998) Identification of Borrelia burgdorferi sensu lato species in Europe. Zentralbl Bakteriol 287:190-5. doi:10.1016/s0934-8840(98)80120-5.
258. Ehrmann S, Ruyts SC, Scherer-Lorenzen M, Bauhus J, Brunet J, Cousins SAO, Deconchat M, Decocq G, De Frenne P, De Smedt P, Diekmann M, Gallet-Moron E, Gartner S, Hansen K, Kolb A, Lenoir J, Lindgren J, Naaf T, Paal T, Panning M, Prinz M, Valdes A, Verheyen K, Wulf M, Liira J (2018) Habitat properties are key drivers of Borrelia burgdorferi (s.l.) prevalence in Ixodes ricinus populations of deciduous forest fragments. Parasit Vectors 11:23. doi:10.1186/s13071-017-2590-x.
259. Gomez-Diaz E, Boulinier T, Sertour N, Cornet M, Ferquel E, McCoy KD (2011) Genetic structure of marine Borrelia garinii and population admixture with the terrestrial cycle of Lyme borreliosis. Environ Microbiol 13:2453-67. doi:10.1111/j.1462-2920.2011.02515.x.
260. Halos L, Mavris M, Vourc'h G, Maillard R, Barnouin J, Boulouis HJ, Vayssier-Taussat M (2006) Broad-range PCR-TTGE for the first-line detection of bacterial pathogen DNA in ticks. Vet Res 37:245-53. doi:10.1051/vetres:2005055.
261. Pichon B, Godfroid E, Hoyois B, Bollen A, Rodhain F, Perez-Eid C (1995) Simultaneous infection of Ixodes ricinus nymphs by two Borrelia burgdorferi sensu lato species: possible implications for clinical manifestations. Emerg Infect Dis 1:89-90. doi:10.3201/eid0103.950304.
262. Alafaci A, Crepin A, Beaubert S, Berjeaud JM, Delafont V, Verdon J (2021) Exploring the Individual Bacterial Microbiota of Questing Ixodes ricinus Nymphs. Microorganisms 9. doi:10.3390/microorganisms9071526.
263. Baranton G, Postic D, Saint Girons I, Boerlin P, Piffaretti JC, Assous M, Grimont PA (1992) Delineation of Borrelia burgdorferi sensu stricto, Borrelia garinii sp. nov., and group VS461 associated with Lyme borreliosis. Int J Syst Bacteriol 42:378-83. doi:10.1099/00207713-42-3-378.
264. Ragagli C, Mannelli A, Ambrogi C, Bisanzio D, Ceballos LA, Grego E, Martello E, Selmi M, Tomassone L (2016) Presence of host-seeking Ixodes ricinus and their infection with Borrelia burgdorferi sensu lato in the Northern Apennines, Italy. Exp Appl Acarol 69:167-78. doi:10.1007/s10493-016-0030-9.
265. Melis S, Batisti Biffignandi G, Olivieri E, Galon C, Vicari N, Prati P, Moutailler S, Sassera D, Castelli M (2024) High-throughput screening of pathogens in Ixodes ricinus removed from hosts in Lombardy, northern Italy. Ticks Tick Borne Dis 15:102285. doi:10.1016/j.ttbdis.2023.102285.

266. Cinco M, Padovan D, Murgia R, Poldini L, Frusteri L, van de Pol I, Verbeek-De Kruif N, Rijpkema S, Maroli M (1998) Rate of infection of Ixodes ricinus ticks with Borrelia burgdorferi sensu stricto, Borrelia garinii, Borrelia afzelii and group VS116 in an endemic focus of Lyme disease in Italy. Eur J Clin Microbiol Infect Dis 17:90-4. doi:10.1007/BF01682162.
267. Beltrame A, Laroche M, Degani M, Perandin F, Bisoffi Z, Raoult D, Parola P (2018) Tick-borne pathogens in removed ticks Veneto, northeastern Italy: A cross-sectional investigation. Travel Med Infect Dis 26:58-61. doi:10.1016/j.tmaid.2018.08.008.
268. Otranto D, Dantas-Torres F, Giannelli A, Latrofa MS, Cascio A, Cazzin S, Ravagnan S, Montarsi F, Zanzani SA, Manfredi MT, Capelli G (2014) Ticks infesting humans in Italy and associated pathogens. Parasit Vectors 7:328. doi:10.1186/1756-3305-7-328.
269. Morganti G, Gavaudan S, Canonico C, Ravagnan S, Olivieri E, Diaferia M, Marenzoni ML, Antognoni MT, Capelli G, Silaghi C, Veronesi F (2017) Molecular Survey on Rickettsia spp., Anaplasma phagocytophilum, Borrelia burgdorferi Sensu Lato, and Babesia spp. in Ixodes ricinus Ticks Infesting Dogs in Central Italy. Vector Borne Zoonotic Dis 17:743-748. doi:10.1089/vbz.2017.2154.
270. Pistone D, Pajoro M, Novakova E, Vicari N, Gaiardelli C, Vigano R, Luzzago C, Montagna M, Lanfranchi P (2017) Ticks and bacterial tick-borne pathogens in Piemonte region, Northwest Italy. Exp Appl Acarol 73:477-491. doi:10.1007/s10493-017-0202-2.
271. Nazzi F, Martinelli E, Del Fabbro S, Bernardinelli I, Milani N, Iob A, Pischiutti P, Campello C, D'Agaro P (2010) Ticks and Lyme borreliosis in an alpine area in northeast Italy. Med Vet Entomol 24:220-6. doi:10.1111/j.1365-2915.2010.00877.x.
272. Rollins RE, Schaper S, Kahlhofer C, Frangoulidis D, Strauss AFT, Cardinale M, Springer A, Strube C, Bakkes DK, Becker NS, Chitimia-Dobler L (2021) Ticks (Acari: Ixodidae) on birds migrating to the island of Ponza, Italy, and the tick-borne pathogens they carry. Ticks Tick Borne Dis 12:101590. doi:10.1016/j.ttbdis.2020.101590.
273. Pintore MD, Ceballos L, Iulini B, Tomassone L, Pautasso A, Corbellini D, Rizzo F, Mandola ML, Bardelli M, Peletto S, Acutis PL, Mannelli A, Casalone C (2015) Detection of Invasive Borrelia burgdorferi Strains in North-Eastern Piedmont, Italy. Zoonoses Public Health 62:365-74. doi:10.1111/zph.12156.
274. Zanet S, Battisti E, Pepe P, Ciuca L, Colombo L, Trisciuoglio A, Ferroglio E, Cringoli G, Rinaldi L, Maurelli MP (2020) Tick-borne pathogens in Ixodidae ticks collected from privately-owned dogs in Italy: a country-wide molecular survey. BMC Vet Res 16:46. doi:10.1186/s12917-020-2263-4.
275. Garcia-Vozmediano A, Giglio G, Ramassa E, Nobili F, Rossi L, Tomassone L (2021) Low Risk Perception about Ticks and Tick-Borne Diseases in an Area Recently Invaded by Ticks in Northwestern Italy. Vet Sci 8. doi:10.3390/vetsci8070131.
276. Audino T, Pautasso A, Bellavia V, Carta V, Ferrari A, Verna F, Grattarola C, Iulini B, Pintore MD, Bardelli M, Cassina G, Tomassone L, Peletto S, Blanda V, Torina A, Caramelli M, Casalone C, Desiato R (2021) Ticks infesting humans and associated pathogens: a cross-sectional study in a 3-year period (2017-2019) in northwest Italy. Parasit Vectors 14:136. doi:10.1186/s13071-021-04603-x.
277. Santino I, Iori A, Nicoletti M, Valletta S, Cimmino C, Scoarughi GL, Santapaola D, Sessa R, Del Piano M (2003) Prevalence of Borrelia Burgdorferi sensu lato genomospecies and of the human granulocytic ehrlichiosis (HGE) agent in Ixodes ricinus ticks collected in the area of Monti Lepini, Italy. Int J Immunopathol Pharmacol 16:105-8. doi:10.1177/039463200301600203.

278. Ciceroni L, Ciarrocchi S, Simeoni J (1998) Antigenic and genomic analysis of a Borrelia burgdorferi sensu stricto strain isolated from Ixodes ricinus ticks in Alto Adige-South Tyrol, Italy. Eur J Epidemiol 14:511-7. doi:10.1023/a:1007432408746.
279. Pajoro M, Pistone D, Varotto Boccazzi I, Mereghetti V, Bandi C, Fabbi M, Scattorin F, Sassera D, Montagna M (2018) Molecular screening for bacterial pathogens in ticks (Ixodes ricinus) collected on migratory birds captured in northern Italy. Folia Parasitol (Praha) 65. doi:10.14411/fp.2018.008.
280. Santino I, del Piano M, Sessa R, Favia G, Iori A (2002) Detection of four Borrelia burgdorferi genospecies and first report of human granulocytic ehrlichiosis agent in Ixodes ricinus ticks collected in central Italy. Epidemiol Infect 129:93-7. doi:10.1017/s0950268802007057.
281. Cinco M, Padovan D, Murgia R, Maroli M, Frusteri L, Heldtander M, Johansson KE, Engvall EO (1997) Coexistence of Ehrlichia phagocytophila and Borrelia burgdorferi sensu lato in Ixodes ricinus ticks from Italy as determined by 16S rRNA gene sequencing. J Clin Microbiol 35:3365-6. doi:10.1128/jcm.35.12.3365-3366.1997.
282. Pecchioli E, Hauffe HC, Tagliapietra V, Bandi C, Genchi C, Rizzoli A (2007) Genospecies of Borrelia burgdorferi sensu lato in Ixodes ricinus ticks from the Autonomous Province of Trento, Italy. Int J Med Microbiol 297:53-9. doi:10.1016/j.ijmm.2006.07.003.
283. Millet I, Ragionieri M, Tomassone L, Trentin C, Mannelli A (2019) Assessment of the Exposure of People to Questing Ticks Carrying Agents of Zoonoses in Aosta Valley, Italy. Vet Sci 6. doi:10.3390/vetsci6010028.
284. Pascucci I, Camma C (2010) Lyme disease and the detection of Borrelia burgdorferi genospecies in Ixodes ricinus ticks from central Italy. Vet Ital 46:173-80, 181-8.
285. Toma L, Mancini F, Di Luca M, Cecere JG, Bianchi R, Khoury C, Quarchioni E, Manzia F, Rezza G, Ciervo A (2014) Detection of microbial agents in ticks collected from migratory birds in central Italy. Vector Borne Zoonotic Dis 14:199-205. doi:10.1089/vbz.2013.1458.
286. Aureli S, Galuppi R, Ostanello F, Foley JE, Bonoli C, Rejmanek D, Rocchi G, Orlandi E, Tampieri MP (2015) Abundance of questing ticks and molecular evidence for pathogens in ticks in three parks of Emilia-Romagna region of Northern Italy. Ann Agric Environ Med 22:459-66. doi:10.5604/12321966.1167714.
287. Garcia-Vozmediano A, Krawczyk AI, Sprong H, Rossi L, Ramassa E, Tomassone L (2020) Ticks climb the mountains: Ixodid tick infestation and infection by tick-borne pathogens in the Western Alps. Ticks Tick Borne Dis 11:101489. doi:10.1016/j.ttbdis.2020.101489.
288. Cacciapuoti B, Ciceroni L, Ciarrocchi S, Khoury C, Simeoni J (1995) Genetic and phenotypic characterization of Borrelia burgdorferi strains isolated from Ixodes ricinus ticks in the Province of Bolzano, Italy. New Microbiol 18:169-81.
289. Ebani VV, Bertelloni F, Turchi B, Filogari D, Cerri D (2015) Molecular survey of tick-borne pathogens in Ixodid ticks collected from hunted wild animals in Tuscany, Italy. Asian Pac J Trop Med 8:714-7. doi:10.1016/j.apjtm.2015.07.033.
290. Favia G, Cancrini G, Carfi A, Grazioli D, Lillini E, Iori A (2001) Molecular identification of Borrelia valaisiana and HGE-like Ehrlichia in Ixodes ricinus ticks sampled in north-eastern Italy: first report in Veneto region. Parassitologia 43:143-6.
291. Corrain R, Drigo M, Fenati M, Menandro ML, Mondin A, Pasotto D, Martini M (2012) Study on ticks and tick-borne zoonoses in public parks in Italy. Zoonoses Public Health 59:468-76. doi:10.1111/j.1863-2378.2012.01490.x.

292. Da Rold G, Ravagnan S, Soppelsa F, Porcellato E, Soppelsa M, Obber F, Citterio CV, Carlin S, Danesi P, Montarsi F, Capelli G (2018) Ticks are more suitable than red foxes for monitoring zoonotic tick-borne pathogens in northeastern Italy. Parasit Vectors 11:137. doi:10.1186/s13071-018-2726-7.
293. Mancini F, Vescio MF, Toma L, Di Luca M, Severini F, Caccio SM, Mariano C, Nicolai G, Laghezza Masci V, Fausto AM, Pezzotti P, Ciervo A (2019) Detection of tick-borne pathogens in ticks collected in the suburban area of Monte Romano, Lazio Region, Central Italy. Ann Ist Super Sanita 55:143-150. doi:10.4415/ANN_19_02_06.
294. Bertolotti L, Tomassone L, Tramuta C, Grego E, Amore G, Ambrogi C, Nebbia P, Mannelli A (2006) Borrelia lusitaniae and spotted fever group rickettsiae in Ixodes ricinus (Acari: Ixodidae) in Tuscany, central Italy. J Med Entomol 43:159-65. doi:10.1603/0022-2585(2006)043[0159:blasfg]2.0.co;2.
295. Cinco M, Padovan D, Murgia R, Heldtander M, Engvall EO (1998) Detection of HGE agent-like Ehrlichia in Ixodes ricinus ticks in northern Italy by PCR. Wien Klin Wochenschr 110:898-900.
296. Tomassone L, Grego E, Auricchio D, Iori A, Giannini F, Rambozzi L (2013) Lyme borreliosis spirochetes and spotted fever group rickettsiae in ixodid ticks from Pianosa island, Tuscany Archipelago, Italy. Vector Borne Zoonotic Dis 13:84-91. doi:10.1089/vbz.2012.1046.
297. Sanogo YO, Parola P, Shpynov S, Camicas JL, Brouqui P, Caruso G, Raoult D (2003) Genetic diversity of bacterial agents detected in ticks removed from asymptomatic patients in northeastern Italy. Ann N Y Acad Sci 990:182-90. doi:10.1111/j.1749-6632.2003.tb07360.x.
298. Bertola M, Montarsi F, Obber F, Da Rold G, Carlin S, Toniolo F, Porcellato E, Falcaro C, Mondardini V, Ormelli S, Ravagnan S (2021) Occurrence and Identification of Ixodes ricinus Borne Pathogens in Northeastern Italy. Pathogens 10. doi:10.3390/pathogens10091181.
299. Cinco M, Banfi E, Trevisan G, Stanek G (1989) Characterization of the first tick isolate of Borrelia burgdorferi from Italy. APMIS 97:381-2.
300. Castro LR, Gabrielli S, Iori A, Cancrini G (2015) Molecular detection of Rickettsia, Borrelia, and Babesia species in Ixodes ricinus sampled in northeastern, central, and insular areas of Italy. Exp Appl Acarol 66:443-52. doi:10.1007/s10493-015-9899-y.
301. Mannelli A, Nebbia P, Tramuta C, Grego E, Tomassone L, Ainardi R, Venturini L, De Meneghi D, Meneguz PG (2005) Borrelia burgdorferi sensu lato infection in larval Ixodes ricinus (Acari: Ixodidae) feeding on blackbirds in northwestern Italy. J Med Entomol 42:168-75. doi:10.1093/jmedent/42.2.168.
302. Cinco M, Padovan D, Murgia R, Frusteri L, Maroli M, van de Pol I, Verbeek-De Kruif N, Rijpkema S, Taggi F (1998) Prevalence of Borrelia burgdorferi infection in Ixodes ricinus in central Italy. Eur J Clin Microbiol Infect Dis 17:134-5. doi:10.1007/BF01682174.
303. Mori E, Pisanu B, Zozzoli R, Solano E, Olivieri E, Sassera D, Montagna M (2018) Arthropods and associated pathogens from native and introduced rodents in Northeastern Italy. Parasitol Res 117:3237-3243. doi:10.1007/s00436-018-6022-4.
304. Sgroi G, Iatta R, Lia RP, Napoli E, Buono F, Bezerra-Santos MA, Veneziano V, Otranto D (2022) Tick exposure and risk of tick-borne pathogens infection in hunters and hunting dogs: a citizen science approach. Transbound Emerg Dis 69:e386-e393. doi:10.1111/tbed.14314.
305. Stefanelli S, Paladini A, Conforti PL, Leoncini F, Vigano S, De Giovannini R, Cinco M (1994) Isolation of Borrelia burgdorferi in Tuscany (Italy). New Microbiol 17:333-6.

306. Diaz-Sanchez S, Hernandez-Jarguin A, Torina A, de Mera IGF, Blanda V, Caracappa S, Gortazar C, de la Fuente J (2019) Characterization of the bacterial microbiota in wild-caught Ixodes ventalloi. Ticks Tick Borne Dis 10:336-343. doi:10.1016/j.ttbdis.2018.11.014.
307. Amore G, Tomassone L, Grego E, Ragagli C, Bertolotti L, Nebbia P, Rosati S, Mannelli A (2007) Borrelia lusitaniae in immature Ixodes ricinus (Acari: Ixodidae) feeding on common wall lizards in Tuscany, central Italy. J Med Entomol 44:303-7. doi:10.1603/0022-2585(2007)44[303:bliiir]2.0.co;2.
308. Cafiso A, Olivieri E, Floriano AM, Chiappa G, Serra V, Sassera D, Bazzocchi C (2021) Investigation of Tick-Borne Pathogens in Ixodes ricinus in a Peri-Urban Park in Lombardy (Italy) Reveals the Presence of Emerging Pathogens. Pathogens 10. doi:10.3390/pathogens10060732.
309. Pistone D, Pajoro M, Fabbi M, Vicari N, Marone P, Genchi C, Novati S, Sassera D, Epis S, Bandi C (2010) Lyme borreliosis, Po River Valley, Italy. Emerg Infect Dis 16:1289-91. doi:10.3201/eid1608.100152.
310. Mancini F, Di Luca M, Toma L, Vescio F, Bianchi R, Khoury C, Marini L, Rezza G, Ciervo A (2014) Prevalence of tick-borne pathogens in an urban park in Rome, Italy. Ann Agric Environ Med 21:723-7. doi:10.5604/12321966.1129922.
311. Grego E, Bertolotti L, Peletto S, Amore G, Tomassone L, Mannelli A (2007) Borrelia lusitaniae OspA gene heterogeneity in Mediterranean basin area. J Mol Evol 65:512-8. doi:10.1007/s00239-007-9029-5.
312. Cerutti F, Modesto P, Rizzo F, Cravero A, Jurman I, Costa S, Giammarino M, Mandola ML, Goria M, Radovic S, Cattonaro F, Acutis PL, Peletto S (2018) The microbiota of hematophagous ectoparasites collected from migratory birds. PLoS One 13:e0202270. doi:10.1371/journal.pone.0202270.
313. Mantelli B, Pecchioli E, Hauffe HC, Rosa R, Rizzoli A (2006) Prevalence of Borrelia burgdorferi s.l. and Anaplasma phagocytophilum in the wood tick Ixodes ricinus in the Province of Trento, Italy. Eur J Clin Microbiol Infect Dis 25:737-9. doi:10.1007/s10096-006-0208-x.
314. Tomassone L, Ceballos LA, Ragagli C, Martello E, De Sousa R, Stella MC, Mannelli A (2017) Importance of Common Wall Lizards in the Transmission Dynamics of Tick-Borne Pathogens in the Northern Apennine Mountains, Italy. Microb Ecol 74:961-968. doi:10.1007/s00248-017-0994-y.
315. Rosa R, Andreo V, Tagliapietra V, Barakova I, Arnoldi D, Hauffe HC, Manica M, Rosso F, Blanarova L, Bona M, Derdakova M, Hamsikova Z, Kazimirova M, Kraljik J, Kocianova E, Mahrikova L, Minichova L, Mosansky L, Slovak M, Stanko M, Spitalska E, Ducheyne E, Neteler M, Hubalek Z, Rudolf I, Venclikova K, Silaghi C, Overzier E, Farkas R, Foldvari G, Hornok S, Takacs N, Rizzoli A (2018) Effect of Climate and Land Use on the Spatio-Temporal Variability of Tick-Borne Bacteria in Europe. Int J Environ Res Public Health 15. doi:10.3390/ijerph15040732.
316. Capelli G, Ravagnan S, Montarsi F, Ciocchetta S, Cazzin S, Porcellato E, Babiker AM, Cassini R, Salviato A, Cattoli G, Otranto D (2012) Occurrence and identification of risk areas of Ixodes ricinus-borne pathogens: a cost-effectiveness analysis in north-eastern Italy. Parasit Vectors 5:61. doi:10.1186/1756-3305-5-61.
317. Piccolin G, Benedetti G, Doglioni C, Lorenzato C, Mancuso S, Papa N, Pitton L, Ramon MC, Zasio C, Bertiato G (2006) A study of the presence of B. burgdorferi, Anaplasma (previously Ehrlichia) phagocytophilum, Rickettsia, and Babesia in Ixodes ricinus collected within the territory of Belluno, Italy. Vector Borne Zoonotic Dis 6:24-31. doi:10.1089/vbz.2006.6.24.
318. Carpi G, Cagnacci F, Wittekindt NE, Zhao F, Qi J, Tomsho LP, Drautz DI, Rizzoli A, Schuster SC (2011) Metagenomic profile of the bacterial communities associated with Ixodes ricinus ticks. PLoS One 6:e25604. doi:10.1371/journal.pone.0025604.

319. Mannelli A, Boggiatto G, Grego E, Cinco M, Murgia R, Stefanelli S, De Meneghi D, Rosati S (2003) Acarological risk of exposure to agents of tick-borne zoonoses in the first recognized Italian focus of Lyme borreliosis. Epidemiol Infect 131:1139-47. doi:10.1017/s0950268803001328.
320. Rizzoli A, Merler S, Furlanello C, Genchi C (2002) Geographical information systems and bootstrap aggregation (bagging) of tree-based classifiers for Lyme disease risk prediction in Trentino, Italian Alps. J Med Entomol 39:485-92. doi:10.1603/0022-2585-39.3.485.
321. Norte AC, Margos G, Becker NS, Albino Ramos J, Nuncio MS, Fingerle V, Araujo PM, Adamik P, Alivizatos H, Barba E, Barrientos R, Cauchard L, Csorgo T, Diakou A, Dingemanse NJ, Doligez B, Dubiec A, Eeva T, Flaisz B, Grim T, Hau M, Heylen D, Hornok S, Kazantzidis S, Kovats D, Krause F, Literak I, Mand R, Mentesana L, Morinay J, Mutanen M, Neto JM, Novakova M, Sanz JJ, Pascoal da Silva L, Sprong H, Tirri IS, Torok J, Trilar T, Tyller Z, Visser ME, Lopes de Carvalho I (2020) Host dispersal shapes the population structure of a tick-borne bacterial pathogen. Mol Ecol 29:485-501. doi:10.1111/mec.15336.
322. Ruzic-Sablijic E, Strle F, Cimperman J (1993) The Ixodes ricinus tick as a vector of Borrelia burgdorferi in Slovenia. Eur J Epidemiol 9:396-400. doi:10.1007/BF00157396.
323. Susnjar J, Cerar Kisek T, Strasek Smrdel K, Ruzic-Sabljic E, Adam K, Ivovic V (2023) Detection, identification and genotyping of Borrelia spp. in ticks of Coastal-Karst and Littoral-Inner Carniola regions in Slovenia. Folia Parasitol (Praha) 70. doi:10.14411/fp.2023.007.
324. Strle F, Cheng Y, Nelson JA, Picken MM, Bouseman JK, Picken RN (1995) Infection rate of Ixodes ricinus ticks with Borrelia afzelii, Borrelia garinii, and Borrelia burgdorferi sensu stricto in Slovenia. Eur J Clin Microbiol Infect Dis 14:994-1001. doi:10.1007/BF01691382.
325. Rijpkema S, Golubic D, Molkenboer M, Verbeek-De Kruif N, Schellekens J (1996) Identification of four genomic groups of Borrelia burgdorferi sensu lato in Ixodes ricinus ticks collected in a Lyme borreliosis endemic region of northern Croatia. Exp Appl Acarol 20:23-30. doi:10.1007/BF00051474.
326. Norte AC, Boyer PH, Castillo-Ramirez S, Chvostac M, Brahami MO, Rollins RE, Woudenberg T, Didyk YM, Derdakova M, Nuncio MS, Carvalho IL, Margos G, Fingerle V (2021) The Population Structure of Borrelia lusitaniae Is Reflected by a Population Division of Its Ixodes Vector. Microorganisms 9. doi:10.3390/microorganisms9050933.
327. Norte AC, Harris DJ, Silveira D, Nunes CS, Nuncio MS, Martinez EG, Gimenez A, de Sousa R, Lopes de Carvalho I, Perera A (2022) Diversity of microorganisms in Hyalomma aegyptium collected from spur-thighed tortoise (Testudo graeca) in North Africa and Anatolia. Transbound Emerg Dis 69:1951-1962. doi:10.1111/tbed.14188.
328. Karasartova D, Gureser AS, Gokce T, Celebi B, Yapar D, Keskin A, Celik S, Ece Y, Erenler AK, Usluca S, Mumcuoglu KY, Taylan-Ozkan A (2018) Bacterial and protozoal pathogens found in ticks collected from humans in Corum province of Turkey. PLoS Negl Trop Dis 12:e0006395. doi:10.1371/journal.pntd.0006395.
329. Guner ES, Hashimoto N, Takada N, Kaneda K, Imai Y, Masuzawa T (2003) First isolation and characterization of Borrelia burgdorferi sensu lato strains from Ixodes ricinus ticks in Turkey. J Med Microbiol 52:807-813. doi:10.1099/jmm.0.05205-0.

330. Sen E, Uchishima Y, Okamoto Y, Fukui T, Kadosaka T, Ohashi N, Masuzawa T (2011) Molecular detection of Anaplasma phagocytophilum and Borrelia burgdorferi in Ixodes ricinus ticks from Istanbul metropolitan area and rural Trakya (Thrace) region of north-western Turkey. Ticks Tick Borne Dis 2:94-8. doi:10.1016/j.ttbdis.2011.03.004.
331. Orkun O, Cakmak A (2019) Molecular identification of tick-borne bacteria in wild animals and their ticks in Central Anatolia, Turkey. Comp Immunol Microbiol Infect Dis 63:58-65. doi:10.1016/j.cimid.2018.12.007.
332. Orkun O, Karaer Z, Cakmak A, Nalbantoglu S (2014) Identification of tick-borne pathogens in ticks feeding on humans in Turkey. PLoS Negl Trop Dis 8:e3067. doi:10.1371/journal.pntd.0003067.
333. Ji Z, Jian M, Yue P, Cao W, Xu X, Zhang Y, Pan Y, Yang J, Chen J, Liu M, Fan Y, Su X, Wen S, Kong J, Li B, Dong Y, Zhou G, Liu A, Bao F (2022) Prevalence of Borrelia burgdorferi in Ixodidae Tick around Asia: A Systematic Review and Meta-Analysis. Pathogens 11. doi:10.3390/pathogens11020143.
334. Orkun O, Cakmak A, Nalbantoglu S, Karaer Z (2020) Turkey tick news: A molecular investigation into the presence of tick-borne pathogens in host-seeking ticks in Anatolia; Initial evidence of putative vectors and pathogens, and footsteps of a secretly rising vector tick, Haemaphysalis parva. Ticks Tick Borne Dis 11:101373. doi:10.1016/j.ttbdis.2020.101373.
335. Polat E, Altinkum SM, Bagdatli Y, Baykara O (2021) The tick fauna in Istanbul, Turkey, from 2013 to 2017 and identification of their pathogens by multiplex PCR: an epidemiological study. Exp Appl Acarol 84:825-834. doi:10.1007/s10493-021-00642-2.
336. Orkun O (2022) Comprehensive screening of tick-borne microorganisms indicates that a great variety of pathogens are circulating between hard ticks (Ixodoidea: Ixodidae) and domestic ruminants in natural foci of Anatolia. Ticks Tick Borne Dis 13:102027. doi:10.1016/j.ttbdis.2022.102027.
337. Zhioua E, Bouattour A, Hu CM, Gharbi M, Aeschliman A, Ginsberg HS, Gern L (1999) Infection of Ixodes ricinus (Acari: Ixodidae) by Borrelia burgdorferi sensu lato in North Africa. J Med Entomol 36:216-8. doi:10.1093/jmedent/36.2.216.
338. Younsi H, Sarih M, Jouda F, Godfroid E, Gern L, Bouattour A, Baranton G, Postic D (2005) Characterization of Borrelia lusitaniae isolates collected in Tunisia and Morocco. J Clin Microbiol 43:1587-93. doi:10.1128/JCM.43.4.1587-1593.2005.
339. Younsi H, Postic D, Baranton G, Bouattour A (2001) High prevalence of Borrelia lusitaniae in Ixodes ricinus ticks in Tunisia. Eur J Epidemiol 17:53-6. doi:10.1023/a:1010928731281.
340. Boucheikhchoukh M, Laroche M, Aouadi A, Dib L, Benakhla A, Raoult D, Parola P (2018) MALDI-TOF MS identification of ticks of domestic and wild animals in Algeria and molecular detection of associated microorganisms. Comp Immunol Microbiol Infect Dis 57:39-49. doi:10.1016/j.cimid.2018.05.002.
341. Benredjem W, Leulmi H, Bitam I, Raoult D, Parola P (2014) Borrelia garinii and Rickettsia monacensis in Ixodes ricinus ticks, Algeria. Emerg Infect Dis 20:1776-7. doi:10.3201/eid2010.140265.
342. Sarih M, Jouda F, Gern L, Postic D (2003) First isolation of Borrelia burgdorferi sensu lato from Ixodes ricinus ticks in Morocco. Vector Borne Zoonotic Dis 3:133-9. doi:10.1089/153036603768395834.

343. Lopez-Cortes L, Lozano de Leon F, Gomez-Mateos JM, Sanchez-Porto A, Obrador C (1989) Tick-borne relapsing fever in intravenous drug abusers. J Infect Dis 159:804.
344. Castilla-Guerra L, Alvarez-Suero J, Del Carmen Fernandez-Moreno M, Fontana ER (2009) Tick-borne relapsing fever: conjunctival haemorrhages. BMJ Case Rep 2009. doi:10.1136/bcr.06.2008.0114.
345. Anda P, Sanchez-Yebra W, del Mar Vitutia M, Perez Pastrana E, Rodriguez I, Miller NS, Backenson PB, Benach JL (1996) A new Borrelia species isolated from patients with relapsing fever in Spain. Lancet 348:162-5. doi:10.1016/s0140-6736(96)02332-x.
346. Toledo A, Anda P, Escudero R, Larsson C, Bergstrom S, Benach JL (2010) Phylogenetic analysis of a virulent Borrelia species isolated from patients with relapsing fever. J Clin Microbiol 48:2484-9. doi:10.1128/JCM.00541-10.
347. Castilla-Guerra L, Marin-Martin J, Colmenero-Camacho MA (2016) Tick-Borne Relapsing Fever, Southern Spain, 2004-2015. Emerg Infect Dis 22:2217-2219. doi:10.3201/eid2212.160870.
348. Dominguez MC, Vergara S, Gomez MC, Roldan ME (2020) Epidemiology of Tick-Borne Relapsing Fever in Endemic Area, Spain. Emerg Infect Dis 26:849-856. doi:10.3201/eid2605.190745.
349. Boyer PH, Koetsveld J, Zilliox L, Sprong H, Talagrand-Reboul E, Hansmann Y, de Martino SJ, Boulanger N, Hovius JW, Jaulhac B (2020) Assessment of Borrelia miyamotoi in febrile patients and ticks in Alsace, an endemic area for Lyme borreliosis in France. Parasit Vectors 13:199. doi:10.1186/s13071-020-04071-9.
350. Franck M, Ghozzi R, Pajaud J, Lawson-Hogban NE, Mas M, Lacout A, Perronne C (2020) Borrelia miyamotoi: 43 Cases Diagnosed in France by Real-Time PCR in Patients With Persistent Polymorphic Signs and Symptoms. Front Med (Lausanne) 7:55. doi:10.3389/fmed.2020.00055.
351. Brouqui P, Stein A, Dupont HT, Gallian P, Badiaga S, Rolain JM, Mege JL, La Scola B, Berbis P, Raoult D (2005) Ectoparasitism and vector-borne diseases in 930 homeless people from Marseilles. Medicine (Baltimore) 84:61-68. doi:10.1097/01.md.0000152373.07500.6e.
352. Ly TDA, Louni M, Hoang VT, Dao TL, Badiaga S, Brouqui P, Tissot-Dupont H, Raoult D, Fournier PE, Gautret P (2020) Epidemiological serosurvey of vector-borne and zoonotic pathogens among homeless people living in shelters in Marseille: cross-sectional one-day surveys (2005-2015). Eur J Clin Microbiol Infect Dis 39:1663-1672. doi:10.1007/s10096-020-03889-6.
353. Malincarne L, Schiaroli E, Ciervo A, Scaglione V, Paciaroni M, Mancini F, Paglia MG, Cardaci S, Pasticci MB, Francisci D, Baldelli F (2019) Meningitis with cranial polyneuritis and cavernous sinus thrombosis by Borrelia crocidurae: First autochthonous case in Europe. Int J Infect Dis 82:30-32. doi:10.1016/j.ijid.2019.02.028.
354. Billiet A, Vanderschueren S, Lagrou K, Pilate T, Fournier PE, Luciani L, Henckaerts L (2022) Tick borne relapsing fever after travelling to a Greek island. J Travel Med 29. doi:10.1093/jtm/taab073.
355. Wood RC, Dixon KC (1945) Tick-borne Relapsing Fever. Br Med J 2:526-8. doi:10.1136/bmj.2.4424.526.
356. Gambles RM, Coghill NF (1948) Relapsing fever in Cyprus. Ann Trop Med Parasitol 42:288-303. doi:10.1080/00034983.1948.11685378.
357. Simon JW (1985) Tick borne relapsing fever imported into the United Kingdom. J R Army Med Corps 131:65-7. doi:10.1136/jramc-131-02-02.

358. Nicholson FD (1919) Tick Fever in Palestine. Br Med J 2:811. doi:10.1136/bmj.2.3077.811.
359. Yagupsky P, Moses S (1985) Neonatal Borrelia species infection (relapsing fever). Am J Dis Child 139:74-6. doi:10.1001/archpedi.1985.02140030076034.
360. Moran-Gilad J, Levine H, Schwartz E, Bartal C, Huerta-Hartal M, Schwaber MJ, Ostfeld I (2013) Postexposure prophylaxis of tick-borne relapsing fever: lessons learned from recent outbreaks in Israel. Vector Borne Zoonotic Dis 13:791-7. doi:10.1089/vbz.2013.1347.
361. McNamara JJ, Kay HH (1988) Relapsing fever (Borrelia) in an adolescent tourist in Israel. J Adolesc Health Care 9:421-3. doi:10.1016/0197-0070(88)90042-3.
362. Koton Y, Bisharat N (2018) Tick-Borne Relapsing Fever with Severe Jarisch-Herxheimer Reaction. Isr Med Assoc J 20:62-63.
363. Hasin T, Davidovitch N, Cohen R, Dagan T, Romem A, Orr N, Klement E, Lubezky N, Kayouf R, Sela T, Keller N, Derazne E, Halperin T, Yavzori M, Grotto I, Cohen D (2006) Postexposure treatment with doxycycline for the prevention of tick-borne relapsing fever. N Engl J Med 355:148-55. doi:10.1056/NEJMoa053884.
364. Fuchs I, Tarabin S, Kafka M (2015) Relapsing Fever: Diagnosis Thanks to a Vigilant Hematology Laboratory. Vector Borne Zoonotic Dis 15:446-8. doi:10.1089/vbz.2014.1764.
365. Assous MV, Wilamowski A, Bercovier H, Marva E (2006) Molecular characterization of tickborne relapsing fever Borrelia, Israel. Emerg Infect Dis 12:1740-3. doi:10.3201/eid1211.060715.
366. Safdie G, Farrah IY, Yahia R, Marva E, Wilamowski A, Sawalha SS, Wald N, Schmiedel J, Moter A, Gobel UB, Bercovier H, Abdeen Z, Assous MV, Fishman Y (2010) Molecular characterization of Borrelia persica, the agent of tick borne relapsing fever in Israel and the Palestinian Authority. PLoS One 5:e14105. doi:10.1371/journal.pone.0014105.
367. Hashavya S, Gross I, Gross M, Hurvitz N, Weiser G, Temper V, Megged O (2020) Tickborne Relapsing Fever, Jerusalem, Israel, 2004-2018. Emerg Infect Dis 26:2420-2423. doi:10.3201/eid2610.181988.
368. Halperin T, Orr N, Cohen R, Hasin T, Davidovitch N, Klement E, Kayouf R, Baneth G, Cohen D, Yavzori M (2006) Detection of relapsing fever in human blood samples from Israel using PCR targeting the glycerophosphodiester phosphodiesterase (GlpQ) gene. Acta Trop 98:189-95. doi:10.1016/j.actatropica.2006.04.004.
369. Yossepowitch O, Gottesman T, Schwartz-Harari O, Soroksky A, Dan M (2012) Aseptic meningitis and adult respiratory distress syndrome caused by Borrelia persica. Infection 40:695-7. doi:10.1007/s15010-012-0296-8.
370. Wengrower D, Knobler H, Gillis S, Chajek-Shaul T (1984) Myocarditis in tick-borne relapsing fever. J Infect Dis 149:1033. doi:10.1093/infdis/149.6.1033.
371. Sidi G, Davidovitch N, Balicer RD, Anis E, Grotto I, Schwartz E (2005) Tickborne relapsing fever in Israel. Emerg Infect Dis 11:1784-6. doi:10.3201/eid1111.050521.

372. Shaked Y, Maier MK, Samra Y (1986) Relapsing fever and salmonella bacteraemia simultaneously affecting a healthy young man. J Infect 13:308-9. doi:10.1016/s0163-4453(86)91718-4.
373. Eisenberg S, Gunders AE, Cohen AM (1968) Tick-borne relapsing fever in the Judean hills, including a case with massive haematuria. Trans R Soc Trop Med Hyg 62:679-81. doi:10.1016/0035-9203(68)90119-3.
374. Helmy N (2000) Seasonal abundance of Ornithodoros (O.) savignyi and prevalence of infection with Borrelia spirochetes in Egypt. J Egypt Soc Parasitol 30:607-19.
375. Dewar HA, Walmsley R (1945) Relapsing fever with nephritis and subarachnoid haemorrhage. Lancet 2:630. doi:10.1016/s0140-6736(45)90763-x.
376. Coghill NF, Lawrence J, Ballantine ID (1947) Relapsing fever in Cyrenaica. Br Med J 1:637-40. doi:10.1136/bmj.1.4505.637.
377. Fotso Fotso A, Angelakis E, Mouffok N, Drancourt M, Raoult D (2015) Blood-Borne Candidatus Borrelia algerica in a Patient with Prolonged Fever in Oran, Algeria. Am J Trop Med Hyg 93:1070-3. doi:10.4269/ajtmh.15-0124.
378. Kahouli S, Naoui H, Uwingabiye J, Reggad A, Ennibi K, Bouchrik M, Lmimouni BE (2018) Relapsing fever in a Moroccan man. Med Sante Trop 28:141-143. doi:10.1684/mst.2018.0792.
379. Diatta G, Souidi Y, Granjon L, Arnathau C, Durand P, Chauvancy G, Mane Y, Sarih M, Belghyti D, Renaud F, Trape JF (2012) Epidemiology of tick-borne borreliosis in Morocco. PLoS Negl Trop Dis 6:e1810. doi:10.1371/journal.pntd.0001810.
380. Leen I, Bruynseels P, Mukadi BK, van Oort M, van den Akker M (2017) A 13-year old girl with pancytopenia at the presentation of a Borrelia hispanica infection: a case report and review of the literature. J Med Case Rep 11:51. doi:10.1186/s13256-017-1225-3.
381. Sarih M, Garnier M, Boudebouch N, Bouattour A, Rihani A, Hassar M, Gern L, Postic D, Cornet M (2009) Borrelia hispanica relapsing fever, Morocco. Emerg Infect Dis 15:1626-9. doi:10.3201/eid1510.090403.
382. Margos G, Pantchev N, Globokar M, Lopez J, Rodon J, Hernandez L, Herold H, Salas N, Civit A, Fingerle V (2020) First Cases of Natural Infections with Borrelia hispanica in Two Dogs and a Cat from Europe. Microorganisms 8. doi:10.3390/microorganisms8081251.
383. Fabbi M, Sambri V, Marangoni A, Magnino S, Solari Basano F, Cevenini R, Genchi C (1995) Borrelia in pigeons: no serological evidence of Borrelia burgdorferi infection. Zentralbl Veterinarmed B 42:503-7. doi:10.1111/j.1439-0450.1995.tb00741.x.
384. Celebi B, Yeni DK, Yilmaz Y, Matur F, Babur C, Oktem MA, Sozen M, Karatas A, Raoult D, Mediannikov O, Fournier PE (2023) Borrelia miyamotoi in wild rodents from four different regions of Turkey. Ticks Tick Borne Dis 14:102143. doi:10.1016/j.ttbdis.2023.102143.
385. Kleinerman G, Eshed T, Nachum-Biala Y, King R, Baneth G (2021) Transmission of the Human Relapsing Fever Spirochete Borrelia persica by the Argasid Tick Ornithodoros tholozani Involves Blood Meals from Wildlife Animal Reservoirs and Mainly Transstadial Transfer. Appl Environ Microbiol 87. doi:10.1128/AEM.03117-20.
386. Kleinerman G, King R, Nachum-Biala Y, Baneth G (2018) Borrelia persica infection in rock hyraxes. Ticks Tick Borne Dis 9:382-388. doi:10.1016/j.ttbdis.2017.12.003.

387. Salant H, Nachum-Biala Y, Zivotofsky D, Tzur TE, Baneth G (2024) Babesia negevi infection in dogs and response to treatment. Ticks Tick Borne Dis 15:102282. doi:10.1016/j.ttbdis.2023.102282.
388. Baneth G, Nachum-Biala Y, Halperin T, Hershko Y, Kleinerman G, Anug Y, Abdeen Z, Lavy E, Aroch I, Straubinger RK (2016) Borrelia persica infection in dogs and cats: clinical manifestations, clinicopathological findings and genetic characterization. Parasit Vectors 9:244. doi:10.1186/s13071-016-1530-5.
389. Schwarzer S, Margos G, Overzier E, Fingerle V, Baneth G, Straubinger RK (2015) Borrelia persica: In vitro cultivation and characterization via conventional PCR and multilocus sequence analysis of two strains isolated from a cat and ticks from Israel. Ticks Tick Borne Dis 6:751-7. doi:10.1016/j.ttbdis.2015.06.012.
390. Baneth G, Dvorkin A, Ben-Shitrit B, Kleinerman G, Salant H, Straubinger RK, Nachum-Biala Y (2022) Infection and seroprevalence of Borrelia persica in domestic cats and dogs in Israel. Parasit Vectors 15:102. doi:10.1186/s13071-022-05223-9.
391. Shwartz D, Nachum-Biala Y, Oren S, Aharoni K, Edery N, Moss L, King R, Lapid R, Straubinger RK, Baneth G (2023) Borrelia persica infection in wild carnivores in Israel: molecular characterization and new potential reservoirs. Parasit Vectors 16:337. doi:10.1186/s13071-023-05953-4.
392. Abdullah H, Elbayoumy MK, Allam AM, Ashry HM, Abdel-Shafy S (2021) Molecular epidemiology of certain vector-borne bacterial microorganisms in domestic animals and their ectoparasites in Egypt. Trop Anim Health Prod 53:484. doi:10.1007/s11250-021-02911-z.
393. Abdullah H, Amanzougaghene N, Dahmana H, Louni M, Raoult D, Mediannikov O (2021) Multiple vector-borne pathogens of domestic animals in Egypt. PLoS Negl Trop Dis 15:e0009767. doi:10.1371/journal.pntd.0009767.
394. Fichet-Calvet E, Jomaa I, Ben Ismail R, Ashford RW (2000) Patterns of infection of haemoparasites in the fat sand rat, Psammomys obesus, in Tunisia, and effect on the host. Ann Trop Med Parasitol 94:55-68. doi:10.1080/00034980057617.
395. Sadeddine R, Diarra AZ, Laroche M, Mediannikov O, Righi S, Benakhla A, Dahmana H, Raoult D, Parola P (2020) Molecular identification of protozoal and bacterial organisms in domestic animals and their infesting ticks from north-eastern Algeria. Ticks Tick Borne Dis 11:101330. doi:10.1016/j.ttbdis.2019.101330.
396. Aouadi A, Leulmi H, Boucheikhchoukh M, Benakhla A, Raoult D, Parola P (2017) Molecular evidence of tick-borne hemoprotozoan-parasites (Theileria ovis and Babesia ovis) and bacteria in ticks and blood from small ruminants in Northern Algeria. Comp Immunol Microbiol Infect Dis 50:34-39. doi:10.1016/j.cimid.2016.11.008.
397. Trape JF, Diatta G, Arnathau C, Bitam I, Sarih M, Belghyti D, Bouattour A, Elguero E, Vial L, Mane Y, Balde C, Prugnolle F, Chauvancy G, Mahe G, Granjon L, Duplantier JM, Durand P, Renaud F (2013) The epidemiology and geographic distribution of relapsing fever borreliosis in West and North Africa, with a review of the Ornithodoros erraticus complex (Acari: Ixodida). PLoS One 8:e78473. doi:10.1371/journal.pone.0078473.
398. Diatta G, Duplantier JM, Granjon L, Ba K, Chauvancy G, Ndiaye M, Trape JF (2015) Borrelia infection in small mammals in West Africa and its relationship with tick occurrence inside burrows. Acta Trop 152:131-140. doi:10.1016/j.actatropica.2015.08.016.

399. Sanz-Aguilar A, Payo-Payo A, Rotger A, Yousfi L, Moutailler S, Beck C, Dumarest M, Igual JM, Miranda MA, Vinas Torres M, Picorelli V, Gamble A, Boulinier T (2020) Infestation of small seabirds by Ornithodoros maritimus ticks: Effects on chick body condition, reproduction and associated infectious agents. Ticks Tick Borne Dis 11:101281. doi:10.1016/j.ttbdis.2019.101281.
400. Socolovschi C, Kernif T, Raoult D, Parola P (2012) Borrelia, Rickettsia, and Ehrlichia species in bat ticks, France, 2010. Emerg Infect Dis 18:1966-75. doi:10.3201/eid1812.111237.
401. Ravagnan S, Tomassone L, Montarsi F, Krawczyk AI, Mastrorilli E, Sprong H, Milani A, Rossi L, Capelli G (2018) First detection of Borrelia miyamotoi in Ixodes ricinus ticks from northern Italy. Parasit Vectors 11:130. doi:10.1186/s13071-018-2713-z.
402. Sakakibara K, Sen E, Sato K, Kawabata H, Ohashi N, Masuzawa T (2016) Detection and Characterization of the Emerging Relapsing Fever Pathogen, Borrelia miyamotoi, from the Ixodes ricinus Tick in the Rural Trakya (Thrace) Region of Northwestern Turkey. Vector Borne Zoonotic Dis 16:797-799. doi:10.1089/vbz.2016.2012.
403. Abdullah H, Aboelsoued D, Farag TK, Abdel-Shafy S, Abdel Megeed KN, Parola P, Raoult D, Mediannikov O (2022) Molecular characterization of some equine vector-borne diseases and associated arthropods in Egypt. Acta Trop 227:106274. doi:10.1016/j.actatropica.2021.106274.
404. Khalil GM, Helmy N, Hoogstraal H, el-Said A (1984) Seasonal dynamics of Ornithodoros (Pavlovskyella) erraticus (Acari: Ixodoidea: Argasidae) and the spirochete Borrelia crocidurae in Egypt. J Med Entomol 21:536-9. doi:10.1093/jmedent/21.5.536.
405. Balti G, Galon C, Derghal M, Souguir H, Guerbouj S, Rhim A, Chemkhi J, Guizani I, Bouattour A, Moutailler S, M'Ghirbi Y (2021) Atelerix algirus, the North African Hedgehog: Suitable Wild Host for Infected Ticks and Fleas and Reservoir of Vector-Borne Pathogens in Tunisia. Pathogens 10. doi:10.3390/pathogens10080953.
406. Bouattour A, Garnier M, M'Ghirbi Y, Sarih M, Gern L, Ferquel E, Postic D, Cornet M (2010) Borrelia crocidurae infection of Ornithodoros erraticus (Lucas, 1849) ticks in Tunisia. Vector Borne Zoonotic Dis 10:825-30. doi:10.1089/vbz.2009.0151.
407. Boularias G, Azzag N, Galon C, Simo L, Boulouis HJ, Moutailler S (2021) High-Throughput Microfluidic Real-Time PCR for the Detection of Multiple Microorganisms in Ixodid Cattle Ticks in Northeast Algeria. Pathogens 10. doi:10.3390/pathogens10030362.
408. Lafri I, El Hamzaoui B, Bitam I, Leulmi H, Lalout R, Mediannikov O, Chergui M, Karakellah M, Raoult D, Parola P (2017) Detection of relapsing fever Borrelia spp., Bartonella spp. and Anaplasmataceae bacteria in argasid ticks in Algeria. PLoS Negl Trop Dis 11:e0006064. doi:10.1371/journal.pntd.0006064.
409. Ouchene N, Nebbak A, Ouchene-Khelifi NA, Dahmani A, Zeroual F, Khelef D, Bitam I, Benakhla A, Parola P (2020) Molecular detection of avian spirochete Borrelia anserina in Argas persicus ticks in Algeria. Comp Immunol Microbiol Infect Dis 68:101408. doi:10.1016/j.cimid.2019.101408.
410. Elbir H, FotsoFotso A, Diatta G, Trape JF, Arnathau C, Renaud F, Durand P (2015) Ubiquitous bacteria Borrelia crocidurae in Western African ticks Ornithodoros sonrai. Parasit Vectors 8:477. doi:10.1186/s13071-015-1089-6.
411. Souidi Y, Boudebouch N, Ezikouri S, Belghyti D, Trape JF, Sarih M (2014) Borrelia crocidurae in Ornithodoros ticks from northwestern Morocco: a range extension in relation to climatic change? J Vector Ecol 39:316-20. doi:10.1111/jvec.12106.

412. Goutier S, Ferquel E, Pinel C, Bosseray A, Hoen B, Couetdic G, Bourahoui A, Lapostolle C, Pelloux H, Garnier M, Sertour N, Pelloux I, Pavese P, Cornet M (2013) Borrelia crocidurae meningoencephalitis, West Africa. Emerg Infect Dis 19:301-4. doi:10.3201/eid1902.121325.
413. Gras E, Bailly E, Le Brun C, Lemaignen A, Lanotte P (2019) Borrelia crocidurae tick-borne relapsing fever upon return from Senegal. Med Mal Infect 49:624-625. doi:10.1016/j.medmal.2019.05.005.
414. Guiheneuf E, Desjardins N, Guiheneuf R (2018) It is not always malaria: diagnosis of Borrelia recurrent fever on blood smear. Ann Biol Clin (Paris) 76:118-119. doi:10.1684/abc.2017.1320.
415. Million M, Cazorla C, Doudier B, La Scola B, Parola P, Drancourt M, Brouqui P (2009) Molecular identification of Borrelia crocidurae in a patient returning from Senegal. BMJ Case Rep 2009. doi:10.1136/bcr.06.2008.0298.
416. Wyplosz B, Mihaila-Amrouche L, Baixench MT, Bigel ML, Berardi-Grassias L, Fontaine C, Hornstein M, Izri A, Baranton G, Postic D (2005) Imported tickborne relapsing fever, France. Emerg Infect Dis 11:1801-3. doi:10.3201/eid1111.050616.
417. Colin de Verdiere N, Hamane S, Assous MV, Sertour N, Ferquel E, Cornet M (2011) Tickborne relapsing fever caused by Borrelia persica, Uzbekistan and Tajikistan. Emerg Infect Dis 17:1325-7. doi:10.3201/eid1707.101894.
418. Antinori S, Mediannikov O, Corbellino M, Raoult D (2016) Louse-borne relapsing fever among East African refugees in Europe. Travel Med Infect Dis 14:110-4. doi:10.1016/j.tmaid.2016.01.004.
419. Colomba C, Scarlata F, Di Carlo P, Giammanco A, Fasciana T, Trizzino M, Cascio A (2016) Fourth case of louse-borne relapsing fever in Young Migrant, Sicily, Italy, December 2015. Mini Review Article. Public Health 139:22-26. doi:10.1016/j.puhe.2016.05.019.
420. Ciervo A, Mancini F, di Bernardo F, Giammanco A, Vitale G, Dones P, Fasciana T, Quartaro P, Mazzola G, Rezza G (2016) Louseborne Relapsing Fever in Young Migrants, Sicily, Italy, July-September 2015. Emerg Infect Dis 22:152-3. doi:10.3201/eid2201.151580.
421. Zammarchi L, Antonelli A, Bartolini L, Pecile P, Trotta M, Rogasi PG, Santini MG, Dilaghi B, Grifoni S, Rossolini GM, Bartoloni A (2016) Louse-Borne Relapsing Fever with Meningeal Involvement in an Immigrant from Somalia to Italy, October 2015. Vector Borne Zoonotic Dis 16:352-5. doi:10.1089/vbz.2015.1928.
422. Antinori S, Tonello C, Edouard S, Parravicini C, Gastaldi D, Grande R, Milazzo L, Ricaboni D, Fenollar F, Raoult D, Corbellino M, Mediannikov O (2017) Diagnosis of Louse-Borne Relapsing Fever despite Negative Microscopy in Two Asylum Seekers from Eastern Africa. Am J Trop Med Hyg 97:1669-1672. doi:10.4269/ajtmh.17-0320.
423. Cutuli SL, De Pascale G, Spanu T, Dell'Anna AM, Bocci MG, Pallavicini F, Mancini F, Ciervo A, Antonelli M (2017) Lice, rodents, and many hopes: a rare disease in a young refugee. Crit Care 21:81. doi:10.1186/s13054-017-1666-5.
424. Grecchi C, Zanotti P, Pontarelli A, Chiari E, Tomasoni LR, Gulletta M, Barbui A, Caligaris S, Matteelli A, Castelli F (2017) Louse-borne relapsing fever in a refugee from Mali. Infection 45:373-376. doi:10.1007/s15010-017-0987-2.

425. Lucchini A, Lipani F, Costa C, Scarvaglieri M, Balbiano R, Carosella S, Calcagno A, Audagnotto S, Barbui AM, Brossa S, Ghisetti V, Dal Conte I, Caramello P, Di Perri G (2016) Louseborne Relapsing Fever among East African Refugees, Italy, 2015. Emerg Infect Dis 22:298-301. doi:10.3201/eid2202.151768.
426. Antinori S, Mediannikov O, Corbellino M, Grande R, Parravicini C, Bestetti G, Longhi E, Ricaboni D, Ehounoud CB, Fenollar F, Raoult D, Rimoldi SG (2016) Louse-Borne Relapsing Fever (Borrelia recurrentis) in a Somali Refugee Arriving in Italy: A Re-emerging Infection in Europe? PLoS Negl Trop Dis 10:e0004522. doi:10.1371/journal.pntd.0004522.
427. Tordini G, Giaccherini R, Corbisiero R, Zanelli G (2006) Relapsing fever in a traveller from Senegal: determination of Borrelia species using molecular methods. Trans R Soc Trop Med Hyg 100:992-4. doi:10.1016/j.trstmh.2005.11.002.
428. Nitzan O, Blum A, Marva E, Katz A, Tzadok BS, Nachum-Biala Y, Baneth G, Peretz A (2017) Case Report: Infectious Diseases in Pilgrims Visiting the Holy Land. Am J Trop Med Hyg 97:611-614. doi:10.4269/ajtmh.17-0097.
429. Hoch M, Wieser A, Loscher T, Margos G, Purner F, Zuhl J, Seilmaier M, Balzer L, Guggemos W, Rack-Hoch A, von Both U, Hauptvogel K, Schonberger K, Hautmann W, Sing A, Fingerle V (2015) Louse-borne relapsing fever (Borrelia recurrentis) diagnosed in 15 refugees from northeast Africa: epidemiology and preventive control measures, Bavaria, Germany, July to October 2015. Euro Surveill 20. doi:10.2807/1560-7917.ES.2015.20.42.30046.
